# Divergent outcomes of anti-PD-L1 treatment coupled with host-intrinsic differences in TCR repertoire and distinct T cell activation states in responding versus non-responding tumors

**DOI:** 10.3389/fimmu.2022.992630

**Published:** 2022-10-18

**Authors:** Jessy John, Rachel A. Woolaver, Vince Popolizio, Samantha M. Y. Chen, Huaibin Ge, Alexandra L. Krinsky, Monika Vashisht, Yonatan Kramer, Zhangguo Chen, Jing H. Wang

**Affiliations:** ^1^ University of Pittsburgh Medical Center (UPMC) Hillman Cancer Center, Division of Hematology and Oncology, Department of Medicine, University of Pittsburgh School of Medicine, Pittsburgh, PA, United States; ^2^ Department of Immunology and Microbiology, University of Colorado Anschutz Medical Campus, School of Medicine, Aurora, CO, United States; ^3^ Department of Immunology, University of Pittsburgh School of Medicine, Pittsburgh, PA, United States

**Keywords:** immunological heterogeneity, head and neck squamous cell carcinoma, single cell-T cell receptor sequencing, individualized anti-tumor immune responses, TCR repertoire

## Abstract

Differential responses to immune checkpoint inhibitors (ICI) may be attributed to tumor-intrinsic factors or environmental cues; however, these mechanisms cannot fully explain the variable ICI responses in different individuals. Here, we investigate the potential contribution of immunological heterogeneity with a focus on differences in T-cell receptor (TCR) repertoire to ICI responses, which has not been defined previously. To reveal additional factors underlying heterogeneous responses to ICI, we employed a squamous cell carcinoma (SCC) mouse model in which tumor-bearing recipients unambiguously diverged into responders (R) or non-responders (NR) upon anti-PD-L1 treatment. Treatment efficacy absolutely required CD8 T-cells and correlated positively with effector functions of CD8 tumor-infiltrating lymphocytes (TILs). We showed that TCR repertoires exhibited a similar magnitude of clonal expansion in R *vs*. NR CD8 TILs. However, the top expanded TCR clonotypes appeared to be mutually exclusive between R and NR CD8 TILs, which also occurred in a recipient-specific manner, demonstrating preferential expansion of distinct TCR clonotypes against the same SCC tumor. Unexpectedly, R *vs*. NR CD8 TILs reached all activation clusters and did not exhibit substantial global differences in transcriptomes. By linking single-cell transcriptomic data with unique TCR clonotypes, CD8 TILs harboring top TCR clonotypes were found to occupy distinct activation clusters and upregulate genes favoring anti-tumor immunity to different extents in R *vs*. NR. We conclude that stochastic differences in CD8 TIL TCR repertoire and distinct activation states of top TCR clonotypes may contribute to differential anti-PD-L1 responses. Our study suggests that host-intrinsic immunological heterogeneity may offer a new explanation for differential ICI responses in different individuals, which could impact on strategies for personalized cancer immunotherapy.

## Introduction

A fundamental question in cancer immunology is why the outcome of anti-tumor immune responses is so heterogeneous and highly variable in different individuals (1). For instance, it is well-known that some cancer patients responded to immune checkpoint inhibitors (ICI), while others were completely unresponsive (2, 3). However, the underlying mechanisms that result in such heterogeneous anti-tumor immune responses remain poorly understood. Elucidating such mechanisms would greatly facilitate the development of more effective personalized cancer immunotherapy. In this regard, prior studies often focused on tumor-intrinsic factors, such as different oncogenic drivers or tumor mutational burden (TMB) as well as environmental factors (e.g., host microbiome) (4–8), which may contribute to heterogeneous anti-tumor immune responses. Nonetheless, a potential contribution of immunological heterogeneity remains poorly defined. For example, it is unknown whether intrinsic differences in the T cell receptor (TCR) repertoire of individuals can influence the outcome of ICI therapy by affecting the frequency and/or variety of CD8 tumor-infiltrating lymphocytes (TILs) in responders versus (*vs*.) non-responders.

Head and neck cancer (HNC) is the sixth most common type of cancer, and 90% of HNC manifest as head and neck squamous cell carcinoma (HNSCC) (9, 10). HNSCC patient samples display highly variable phenotypes regarding TMB and tumor infiltration of T cells and other immune cells (11–14). ICIs including antibodies against programmed death 1 (PD1) or PD ligand 1 (PD-L1) were investigated in treating HNSCCs; however, treatment efficacy varies substantially in different patients and the response rate remains relatively low (15–22). Differential responses to ICI treatment may be partially attributed to the immunological heterogeneity in individual HNSCC patients, indicated by a highly variable level of T cell infiltration before treatment (23, 24).

CD8 T cells can kill tumor cells, exhibit a strong correlation with patient survival and ICI response, and thus have been extensively studied in the context of anti-tumor immunity (25–27). Several reviews have comprehensively summarized recent studies using single-cell approach to analyze CD8 TILs in cancer patients (28–30). Nevertheless, it is very difficult to identify common principles governing anti-tumor immune responses due to the substantial differences between patients and their tumors. In mouse tumor models, tumor antigen-specific CD8 T cell responses were often studied using immunodominant antigens such as AH1 and PMEL (31, 32) or model antigens such as ovalbumin (OVA) that can be introduced into tumors, and their corresponding transgenic T cells such as 1D4 and Pmel-1 (33, 34) or OT-I T cells (35). However, these models are not suitable to address whether and how the diverse composition of polyclonal T cells shapes the outcome of ICI treatment. Effective anti-tumor immune responses are normally mediated by polyclonal T cells that may recognize distinct tumor antigens with various affinities. Hence, the efficacy of ICI treatment may also require polyclonal CD8 T cell-mediated anti-tumor immunity; yet we lack a well-controlled model system to delineate dynamic changes in TCR repertoire and transcriptome of CD8 TILs at a single-cell resolution, with such changes correlating to differential ICI responses.

Most conventional T cells are αβ T cells whose TCRs consist of a α chain and a β chain that are encoded by *TRA* and *TRB*, respectively, and linked by disulfide bonds. Both TCRα and TCRβ chains are generated by a somatic DNA recombination process, called V(D)J recombination (36, 37), which occurs in a random and stochastic manner in different individuals. TCRs can be clustered by distinct “clonotypes” composed of unique TCRα and TCRβ chains containing specific V(D)J gene segments and complementarity-determining region 3 (CDR3). CDR3 covers the highly divergent junction of V(D)J recombination and serves as a barcode for TCR specificity. To study the formation and diversity of the human TCR repertoire, humanized mouse models were generated by implanting immunodeficient mice with human hematopoietic stem cells (HSCs) and human thymus from the same or different donors (38). Although these humanized mice have identical HSCs, thymi, genetic background and environment, human TCR repertoires are formed stochastically and are totally divergent (38). These data indicate that each individual, including identical twins, has an almost completely different TCR repertoire. However, it remains poorly understood how stochastically generated TCR repertoire and its selection in the tumor microenvironment (TME) can together influence the outcome of ICI treatment.

To elucidate the underlying mechanisms of heterogeneous anti-tumor immune responses, we previously employed a Kras^G12D^Smad4^-/-^ SCC cell line, termed A223, that has been characterized earlier (39–41). When we transplanted A223 tumors into genetically identical wild-type (WT) C57BL/6 (B6) recipient mice, a small fraction of recipients spontaneously eradicated their tumors without intervention (Regressors) while the majority of recipients succumbed to tumor progression (Progressors) (42). Such heterogeneous anti-tumor responses were dependent on CD8 T cells. We found that the top expanded TCR clonotypes of CD8 TILs were almost mutually exclusive between Regressors and Progressors (42). Overall, A223 model demonstrates a detectable level of heterogeneity during spontaneous anti-tumor immune responses, although it remains unknown whether and how ICI treatment affects CD8 TIL TCR repertoires in A223 model.

In the current study, we employed A223 model to elucidate the underlying mechanisms of differential ICI responses. Upon anti-PD-L1 treatment, tumor-bearing recipient mice diverged into responders, slow-progressors, or non-responders. We performed single-cell RNA-sequencing (scRNA-seq) and single-cell TCR V(D)J sequencing for CD8 TILs and splenic CD8 T cells from responders and non-responders. CD8 TILs underwent clonal expansion similarly regardless of responder or non-responder state. In contrast, the top expanded TCR clonotypes differ remarkably between responders and non-responders. Furthermore, many top TCR clonotypes were detected only in one recipient, indicating a highly individualized anti-tumor immune response. By coupling deep transcriptomic analysis with unique TCR clonotypes, we found that CD8 TILs with top expanded TCR clonotypes occupied distinct activation clusters in responders *vs*. non-responders. Our data reveal that stochastic differences in TIL TCR repertoire and distinct activation states of top TCR clonotypes may contribute to differential anti-PD-L1 responses.

## Material and methods

### *In vivo* mouse work and tumor injection

A223 tumor line was described previously (39). Tumor cells were injected into wild-type (WT) C57BL/6 (B6) (Stock no. 000664) or B6.129S2-Cd8a^tm1Mak^/J (CD8^-/-^) mice (Stock no. 002665) (Jackson Laboratories). Both male and female mice (6-8 weeks) were used for the study. When tumor size reached 2 cm in any dimension or other humane end points were met, mice were euthanized in accordance with institutional guidelines. All mice were maintained under specific pathogen-free conditions in the vivarium facility of University of Colorado Anschutz Medical Campus (Aurora, CO) or in the UPMC Hillman Cancer Center Animal Facility (Pittsburgh, PA). Animal work was approved by the Institutional Animal Care and Use Committee of University of Colorado Anschutz Medical Campus (AMC) (Aurora, CO) and University of Pittsburgh (Pittsburgh, PA).

A223 cells were cultured in complete DMEM media supplemented with 10% fetal bovine serum (FBS), 20mM HEPES buffer, 1×antibiotic-antimycotic at 37°C CO_2_ incubator (5%) until 90% confluent. For tumor injection, A223 cells were washed with phosphate buffered saline (PBS), trypsinized (0.01% Trypsin-EDTA, Fisher Scientific) and washed sequentially with complete DMEM media or PBS. 100,000 A223 cells were suspended in PBS and 50% Matrigel Basement Membrane Matrix (Corning) to a final volume of 100µL and injected subcutaneously into one flank of each mouse or into one cheek per mouse. Tumor length and width were measured with calipers, and tumor volume was calculated as (length×width^2^)/2.

### Anti-PD-L1 treatment and assessment of treatment effects

When tumor size reached ~250-350mm^3^, tumor-bearing mice were treated with anti-PD-L1 (200µg/mouse/dose, clone 10F.9G2, BioXCell, Catalog# BE0101) by intraperitoneal (i.p.) injection diluted in PBS for 3 times (2-day interval) or PBS only as vehicle control. To assess treatment effects, relative change in tumor volume (RCTV) was calculated as the change in tumor volume (TV) from the start of treatment (TV_0_) to the TV at day n (the endpoint of control group) (TV_n_) divided by TV_0_ (RCTV=[TV_n_−TV_0_]/TV_0_). Based on the RCTV, anti-PD-L1 treated recipients were divided into responders (RCTV<0), slow progressors (0<RCTV ≤ 1.5) and non-responders (RCTV>1.5). For example, if tumor-bearing mice were treated on day 12 (the start of treatment), day 14, and day 16 with anti-PD-L1, and tumors were collected and analyzed on day 20, the RCTV would be calculated as follows: RCTV= (TV_day20_−TV_day12_)/TV_day12_.

### Flow cytometry

Spleens were mechanically dissociated into single cell suspensions, and red blood cells (RBC) were lysed using RBC lysing buffer (Sigma Aldrich). Tumors were minced with razor blades and dissociated with 50µg/ml Liberase DL (Sigma-Aldrich) in plain DMEM for 30 min at 37°C. Digested tumors were mashed through 70µm filters, washed with 1×PBS (2% FBS), and cells were RBC lysed and filtered with cell strainers to prepare single cell suspensions. Single-cell suspensions were immediately stained with flow cytometry antibodies or used for *ex vivo* stimulation followed by antibody staining. For *ex vivo* stimulation, cells were cultured in 12-well plate (2−5×10^6^ cells/sample/well) for 4 hours at 37°C in the presence of phorbol 12-myristate 13-acetate (PMA) (40nM) and Ionomycin (650nM) (LC Laboratories), and Brefeldin A (BFA) Solution (1×) (BD Biosciences, Catalog# 347688) in DMEM complete media with β-mercaptoethanol (100µM). Stimulated cells were harvested, washed, and stained as follows. Dead cells were excluded by LIVE/DEAD (1:1000) fixable Aqua Dead Cell Stain (Invitrogen) followed by surface staining, fixation/permeabilization, and intracellular or intranuclear staining. TruStain FcX™ (anti-mouse CD16/32) antibody (BioLegend) and Brilliant Stain Buffer Plus (BD Horizon) were added into each flow panel mixture according to manufacturer’s instructions. For intracellular staining of IFN-γ and TNF-α, cells were fixed and permeabilized with the BD Fixation/Permeabilization kit. For intranuclear staining of T-bet, Nur77, Ki67, and EOMES, cells were fixed and permeabilized with the BD Mouse FoxP3 Buffer Set. Antibodies used for flow cytometry were listed in **Supplemental Table 8**. Data were acquired on BD LSRFortessa X-20 cytometer and analyzed using FlowJo™ software V10 (FLOWJO).

### Coupled single cell RNA-seq and single-cell TCR V(D)J sequencing

Tumors and spleens from 4 responder (R) and 4 non-responder (NR) mice were harvested and single-cell suspensions were prepared as described above. Single-cell suspensions of tumor and spleen from the first cohort (R1 and NR1) were stained with Live/Dead Green and antibodies against CD45, TCRβ, CD3, CD8, CD4 or just Live/Dead Green, and sorted using the MoFlo XDP100 for alive CD8 T cells (gated on Live, CD45^+^, TCRβ^+^/CD3^+^, CD8^+^CD4^−^ cells). Single-cell suspensions from the second, third and fourth cohort (R2-R4 and NR2-NR4) were subjected to EasySep™ Mouse CD8a Positive Selection Kit II (StemCell Technologies, Catalog#18953) according to manufacturer’s instructions to purify CD8 T cells. Detailed information of all sequenced samples was in **Supplemental Table 1**. Sorted or purified samples were submitted to the Genomics and Microarray Core (University of Colorado AMC) or Genomics Research Core (University of Pittsburgh) for single cell capture and library preparation. Cells were loaded into 10×Genomics Chromium Next GEM Chip K (Catalog#1000286) for the 5’ captures. Single-cell gene expression libraries were prepared using Chromium Next GEM Single Cell 5’ Kit v2, (Catalogue#1000265) according to the manufacturer’s instructions. 5’ libraries were split in half, one for RNA-seq and another half was enriched for TCR sequencing using the Chromium Single Cell Mouse TCR Amplification Kit (Catalogue#1000254). Samples were sequenced on the Illumina NovaSeq 6000 platform (University of Colorado AMC Genomics and Microarray Core or UPMC Genome Center) for an estimated read depth of 50,000 reads per cell (5’ expression), or 5,000 reads per cell (TCR VDJ). RNA-seq reads were mapped to mm10 (mouse reference genome) using 10×Genomics CellRanger (version 4.0.0) count pipeline and VDJ sequencing reads were mapped to the GRCm38 reference dataset using CellRanger VDJ (version 4.0.0).

### Single-cell TCR VDJ analysis

Eight TIL (RTIL1-4 and NRTIL1-4) and eight spleen samples (RSP1-4 and NRSP1-4) were sequenced for TCR VDJ region (**Supplemental Table 1**). Filtered_contig_annotations output files from the 10×Genomics CellRanger VDJ pipeline were used for further VDJ analysis using R (version 4.1.0) or for analysis using immunarch (version 0.6.7) package (43) of R. Cells were filtered in R as follows: firstly, including cells with only full length, productive, high-confidence V and J segments and secondly, including cells containing only 1 TCRβ chain, and only 1 or 2 TCRα chains (due to lack of allelic exclusion in TCRα locus). CD8 T cells from each sample were grouped into clones by identical nucleotide sequences of the TCRα and TCRβ CDR3 chains, while cells with the same a.a. sequence for the TCRα and TCRβ CDR3 chains were grouped into the same clonotype. Clonotypes were quantified to calculate the percent in each sample (% of a given clonotype = # of cells with that clonotype/# of total cells in the sample) and clonotypes above 1% in any of the 8 TIL samples were used for analysis of “top clonotypes” in further VDJ analyses. Clonotypes above 0.65% in R TIL samples (RTIL1-4) and clonotypes above 1% in NR TIL samples (NRTIL1-4) were used for analysis of “top clonotypes” in single-cell gene expression analyses to include equal numbers of clonotypes from R and NR.

### GLIPH TCR analysis

TCR specificity groups were analyzed using GLIPH (Grouping of Lymphocyte Interactions by Paratope Hotspots) algorithm (44) that employed a human reference database. GLIPH analysis was performed on 8 TIL samples as described previously (42). Mouse reference database was constructed from the list of unique CDR3β sequences in all 16 of our VDJ samples (8 TILs and 8 spleens) combined with the supplementary data from previous studies (42, 45), totaling 151,775 unique mouse CDR3β sequences. CDR3β sequences from each of our 8 TIL samples (RTIL1-4 and NRTIL1-4) were input into GLIPH using the constructed mouse reference database. Network plots were created as described previously (42), where one plot included all members of each group. Each node represents a TCRβ CDR3 sequence in the group, and each line represents a global (thick line) or local (thin line) similarity to another CDR3 sequence. Node size for each plot was calculated as 100 * [(% in RTIL1 + % in RTIL2 + % in RTIL3 +% in RTIL4)/4 + (% in NRTIL1 + % in NRTIL2 + % in NRTIL3 + % in NRTIL4)/4]. Node color (“Relative Ratio”) was calculated as [(% in NRTIL1 + % in NRTIL2 + % in NRTIL3 + % in NRTIL4)/4]/[(% in RTIL1 + % in RTIL2 + % in RTIL3 +% in RTIL4)/4 + (% in NRTIL1 + % in NRTIL2 + % in NRTIL3 + % in NRTIL4)/4], where blue nodes are sequences only in R samples and red nodes are sequences only in NR samples. Values in both NR and R were graded from red (1.0) to purple to blue (0.0) according to the ratio. Network plots were generated using the networkD3 package.

### Single-cell gene expression analysis

The single-cell gene expression analysis was performed on 8 TIL samples (RTIL1-4, NRTIL1-4) and 8 spleen samples (RSP1-4, NRSP1-4) using Seurat version 4.0.2 (46). From each sample, low quality cells (cells with <500 features detected or >10% mitochondrial RNA content) and the presumed doublets (outlier cells in the scatter plot between the number of genes detected in each cell (nFeature_RNA) and the total number of molecules per cell (nCount_RNA)) were removed from further analysis. TCR clonotype information (CDR3α and CDR3β a.a. sequences) was added as metadata for each cell. The 16 samples were processed using the following functions: NormalizeData, FindVariableFeatures, FindIntegrationAnchors and IntegrateData, CellCycleScoring, ScaleData as described previously (42). Dimensionality reduction was performed by running principal component analysis with RunPCA function and integrated variable features were then used to cluster and visualize all cells by UMAP analysis with RunUMAP, FindNeighbors, and FindClusters functions on the first 30 principal components. CD8 T cell clusters were defined based on the cluster’s overall expression of *Cd3e, Cd8, Cd3d*, and the lack of *Cd4* and/or *Foxp3* and the cells in the CD8 T cell clusters were extracted for further analysis. FindMarkers function was used to identify the differentially expressed genes in each cluster and individual clusters were then named based on the highly expressed genes in each cluster. Seurat’s FindConservedMarkers function was used to determine differential gene expression (DEG) between R and NR, controlling for cohort (Cohort 1: R1 and NR1; Cohort 2: R2 and NR2; Cohort 3: R3 and NR3, Cohort 4: R4 and NR4). DEG was calculated for cells in one cohort at a time, and then results were consolidated by taking the most conservative p-value and average ln(fold change) among cohorts. Fold changes were calculated as e^ln(fold change) if ln(fold change)>0, and -1/e^ln(fold change) if ln(fold change)<0.

### Statistical analysis

Data were presented as mean ± SEM. Statistical significance was calculated with unpaired t test or one-way ANOVA followed by Tukey’s multiple comparison test or two-way ANOVA followed by Sidak’s multiple comparison test or Fisher’s Exact test. Analysis was performed using GraphPad Prism version 9.3.1 for Windows (GraphPad Software).

## Results

### Anti-PD-L1 treatment led to heterogeneous responses in A223 tumor-bearing recipients

**Figure 1:**
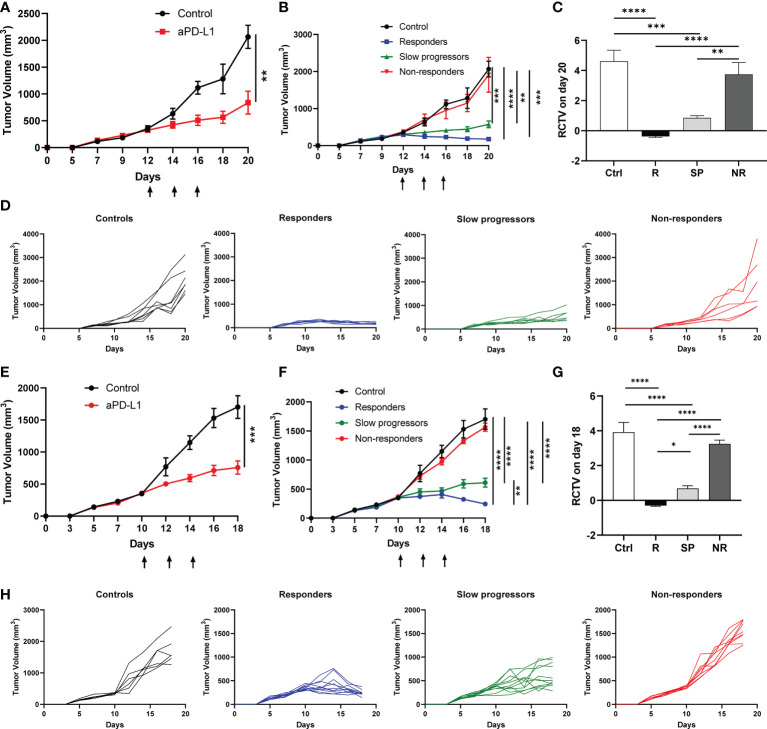
Differential responses to anti-PD-L1 treatment in A223 tumor-bearing mice. **(A–D)** A223 tumor cells (1×10^5^) were injected s.c. into one flank of WT B6 mice (n=30, 90% take rate). Tumor-bearing mice were randomized and treated with control or anti-PD-L1 Ab at day 12 (arrows indicate treatment days) after tumor injection. Tumor growth was monitored for 20 days. **(A)** Overall tumor growth curves of control (n=7) and anti-PD-L1-treated (n=20) mice. **(B)** Tumor growth curves of control, R, SP and NR groups. According to RCTV on day 20, anti-PD-L1 treated recipients diverged into R (n=7, RCTV<0), SP (n=7, 0<RCTV ≤ 1.5) and NR (n=6, RCTV>1.5). **(C)** RCTV of control and different treatment groups (R, SP and NR) calculated as (TV_day20_-TV_day12_)/TV_day12_. **(D)** Individual tumor growth curves of control, R, SP, and NR groups. **(E–H)** A223 tumor cells (1×10^5^) were injected into the buccal region (cheek) of WT B6 mice (n=41, 90% take rate). Tumor-bearing mice were randomized and treated with control or anti-PD-L1 Ab at day 10 (arrows indicate treatment days) after tumor injection. Tumor growth was monitored for 18 days. **(E)** Overall tumor growth curves of control (n=6) and anti-PD-L1-treated mice (n=31). **(F)** Tumor growth curves of control, R, SP and NR groups. According to RCTV on day 18, anti-PD-L1 treated recipients diverged into R (n=11, RCTV<0), SP (n=11, 0<RCTV ≤ 1.5) and NR (n=9, RCTV>1.5). **(G)** RCTV of control and different treatment groups (R, SP and NR) calculated as (TV_day18_-TV_day10_)/TV_day10_. **(H)** Individual tumor growth curves of control, R, SP, and NR groups. Data were presented as mean ± SEM. Statistical significance was calculated with unpaired t test or one-way ANOVA followed by Tukey’s multiple comparisons test (*P<0.05; **P<0.01; ***P<0.001; ****P<0.0001).

To elucidate the mechanisms underlying heterogeneous ICI responses, we took advantage of a Kras^G12D^Smad4^-/-^ SCC cell line that was previously characterized (39–41), termed A223. We first transplanted A223 tumors into genetically identical WT B6 recipient mice at the flank. Consistent with our previous studies, tumors initially grew in 100% recipients and continued to grow aggressively in most recipients, whereas about 10% of recipients spontaneously eliminated tumors (data not shown) that were excluded from subsequent studies. When tumor size reached ~250-350mm^3^, tumor-bearing mice were randomized into two groups: control *vs*. anti-PD-L1, treated with PBS or anti-PD-L1 monoclonal antibody (mAb), respectively. Anti-PD-L1 treatment significantly inhibited tumor growth (**Figure 1A**). Intriguingly, anti-PD-L1 treated recipients diverged into three groups: responders (R), slow progressors (SP) and non-responders (NR) (**Figure 1B**), defined based on relative change in tumor volume (RCTV) at the end of experiments (**Figure 1C**) (see details in Method). Tumor growth was significantly inhibited in R compared to control, while NR failed to respond to anti-PD-L1 completely (**Figures 1B–D**). Consistent results were obtained in different treatment cohorts (**Supplemental Figures 1A–D, E–H**).

We next tested whether tumors injected at different anatomical location would elicit different responses to anti-PD-L1 treatment. A223 tumors were orthotopically transplanted into the cheek region of WT B6 recipients. Tumor-bearing recipients were randomized and treated as described above. Consistently, we observed that tumor-bearing mice still diverged into R, SP and NR groups (**Figures 1E–H**). Taken together, our data showed that anti-PD-L1 treatment led to highly heterogeneous responses in genetically identical WT B6 tumor-bearing recipients regardless of tumor anatomical location.

### Efficacy of anti-PD-L1 treatment depends on CD8 T cells

**Figure 2:**
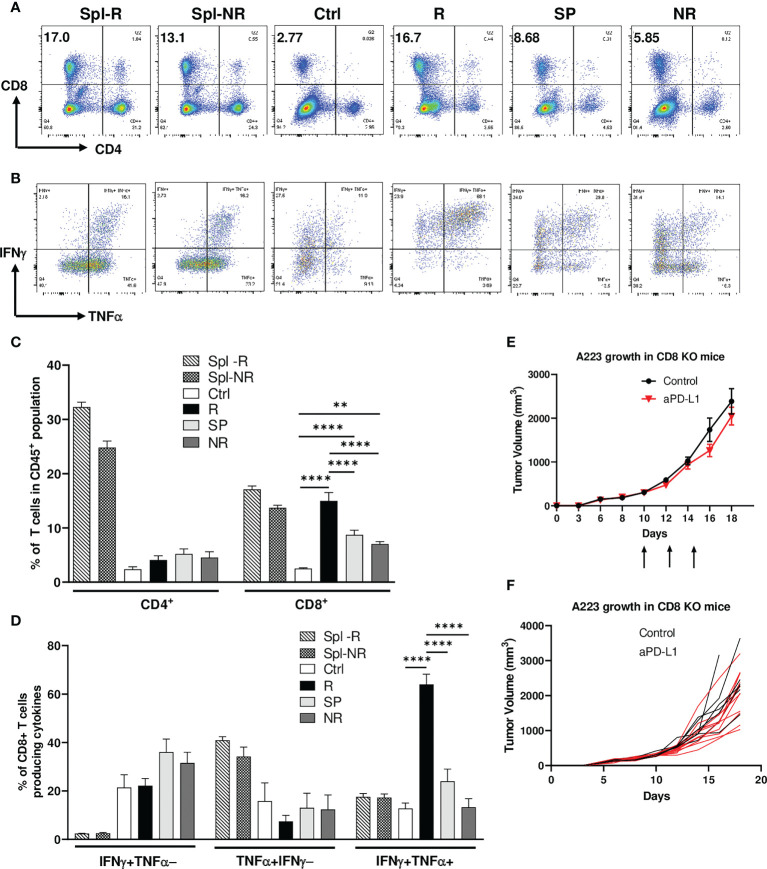
Efficacy of anti-PD-L1 treatment depends on CD8 T cells. Tumor-bearing recipients were treated as indicated on day 14, 16 and 18 after tumor inoculation. Spleens and tumors were harvested on day 21 after inoculation. Cells were stained and analyzed by flow cytometry. **(A, C)** Correlation of CD8 TIL percentage with anti-PD-L1 responses. Representative flow plots **(A)** and percentages **(C)** of CD4 and CD8 T cells within CD45^+^ population of splenic samples from responder (R) (Spl-R, n=5) or non-responder (NR) mice (Spl-NR, n=6), or tumor samples from control (Ctrl, n=9), R (n=5), slow progressor (SP) (n=6) and NR (n=6) mice. **(B, D)** Correlation of IFN-γ/TNF-α-double producing CD8 TILs with anti-PD-L1 responses. Representative flow plots **(B)** and quantification of the percentages **(D)** of stimulated CD8 T cells producing IFNγ alone (IFNγ^+^TNFα^−^, top left quadrant in panel B), TNFα alone (TNFα^+^IFNγ^−^, bottom right quadrant in panel B), or both cytokines (IFNγ^+^TNFα^+^, top right quadrant in panel B) in Spl-R (n=5), Spl-NR (n=6), Ctrl (n=9), R (n=5), SP (n=6) and NR (n=6). Data are from a single experiment which are representative of 3 independently repeated cohort experiments. **(E, F)** Treatment efficacy depends on CD8 T cells. A223 tumors (1×10^5^ cells) were injected s.c. into one flank of CD8^-/-^ mice. When tumor size reached ~300mm^3^, recipients were treated with control (n=7) or anti-PD-L1 (n=11). Tumor growth was monitored for 18 days. Overall **(E)** or individual **(F)** tumor growth curves of control and anti-PD-L1 treated recipients. Data were presented as mean ± SEM. Statistical significance was calculated with one-way ANOVA followed by Tukey’s multiple comparisons test (**, *P*<0.01; ****, *P*<0.0001).

To delineate the mechanisms of heterogeneous responses to anti-PD-L1, we performed flow cytometry analysis on the TILs of control, R, SP and NR groups as well as the spleens of R and NR. We found that the percentage of CD4 TILs within CD45^+^ population did not differ significantly in control, R, SP and NR groups (**Figures 2A, C**). However, the percentage of CD8 TILs was significantly higher in R compared to control, SP and NR groups (**Figures 2A, C**); furthermore, it was also significantly higher in SP or NR compared to control group (**Figures 2A, C**). These data suggest that more CD8 TILs infiltrated the tumors in anti-PD-L1 treated recipients than controls, albeit to a different extent in R, SP and NR groups.

To examine the effector functions of CD8 TILs, we stimulated them *ex vivo* for 4 hrs and performed intracellular cytokine staining. We found that CD8 TILs in R group produced more IFN-γ and TNF-α simultaneously, so-called IFNγ^+^TNFα^+^ double producers, than those in all other groups (**Figures 2B, D**). In contrast, the percent of IFN-γ^+^TNFα^−^ or TNF-α^+^IFNγ^−^ population, namely single producers, in CD8 TILs did not differ significantly in control, R, SP and NR groups (**Figures 2B, D**). When we combined both single and double producers, the percentage of IFNγ^+^ (IFNγ^+^TNFα^−^ plus IFNγ^+^TNFα^+^) or TNFα^+^ (TNFα^+^IFNγ^−^ plus IFNγ^+^TNFα^+^) population in CD8 TILs was significantly higher in R than NR (**Supplemental Figures 2A, B**), consistent with a critical role of IFNγ in the context of ICI treatment (47, 48). These data show that CD8 T cell activation may play a critical role in mediating anti-PD-L1 response. To further test this notion, we injected A223 tumors into CD8^-/-^ mice and found that these mice completely failed to respond to anti-PD-L1 (**Figures 2E, F**), demonstrating that CD8 T cells were required for the efficacy of anti-PD-L1 treatment.

Next, we examined the expression level of different checkpoint molecules on CD8 TILs in different groups as well as in splenic controls of R and NR groups. Our data showed that splenic CD8 T cells did not express PD-1, LAG-3, or TIM-3 (**Supplemental Figures 2C–G**). In contrast, CD8 TILs expressed a high level of PD-1 and LAG-3 but not TIM-3 (**Supplemental Figures 2C–E**); furthermore, CD8 TILs co-expressed PD-1 and LAG-3 in all groups including control, R, SP and NR (**Supplemental Figure 2F**). Anti-PD-L1 treatment did not affect the expression level of any checkpoints (**Supplemental Figure 2G**). These data suggest that CD8 TILs were highly activated in control and anti-PD-L1 treated groups, whereas anti-PD-L1 treatment had no effects on the checkpoint expression of CD8 TILs.

### Top TCR clonotypes appear to be mutually exclusive in R *vs*. NR CD8 TILs

**Figure 3:**
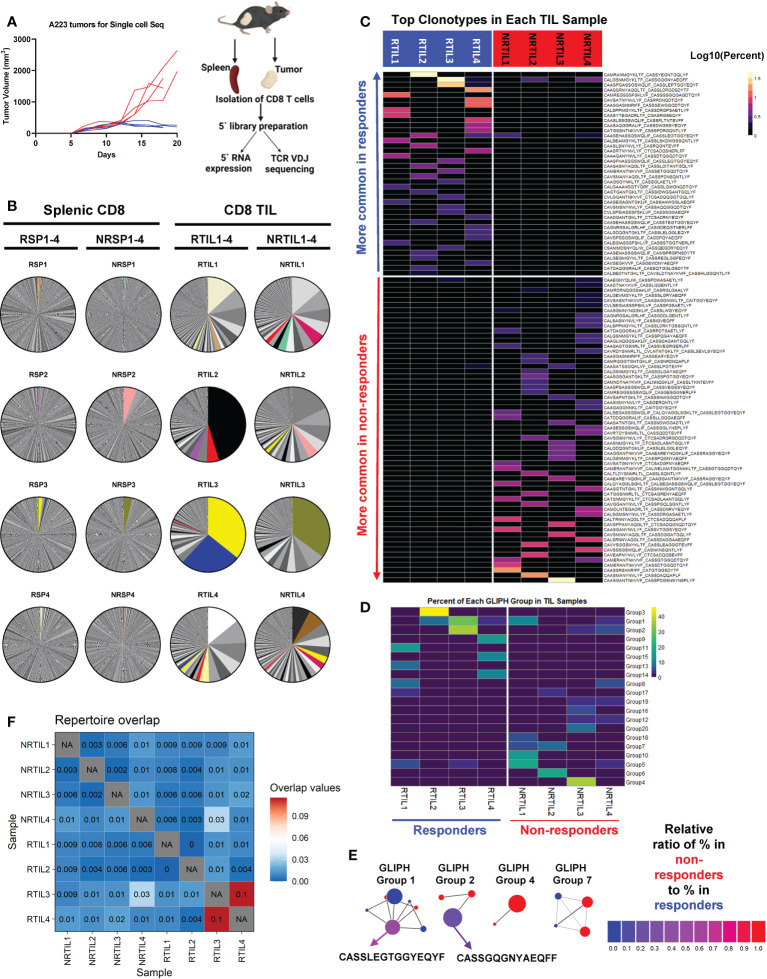
Top TCR clonotypes appear to be mutually exclusive in responder (R) *vs*. non-responder (NR) CD8 TILs. **(A)** Left panel: Tumor growth curves of R (RCTV<0, blue, n=4) and NR (RCTV>1.5, red, n=4) mice. Right panel: experimental scheme for single-cell sequencing. Tumors and spleens were harvested on day 20 for cohort 1 and 2 or day 18 for cohort 3 and 4 after tumor inoculation. CD8 T cells were isolated and subjected for coupled scRNA-seq and single-cell TCR V(D)J sequencing (both CD8 TIL and splenic CD8 T cells) using 10×Genomics platform. **(B)** Distribution of TCR clonotypes (defined as paired a.a. sequences of CDR3α and CDR3β regions) in R (RTIL1-4) and NR (NRTIL1-4) CD8 TILs as well as R (RSP1-4) and NR (NRSP1-4) splenic CD8 T cells. In each sample, a single pie slice represents the percent of cells harboring the same TCR clonotype in the entire sample. Clonotypes shared between samples are colored. **(C)** Heatmap of top clonotypes (>1% of a given sample) sorted by average abundance in R *vs*. average abundance in NR. Clonotypes are colored according to the log10 percent in each sample. **(D)** Heatmap of top 20 GLIPH groups identified in 8 TIL samples (RTIL1-4, NRTIL1-4) based on TCRβ CDR3 sequences. Groups are ordered based on their average percent in R samples (RTIL1-4) *vs*. average percent in NR samples (NRTIL1-4). **(E)** Network plots of GLIPH groups (Groups-1, 2, 4, 7). Each node means a TCRβ CDR3 sequence, and each line means a global (thick line) or local (thin line) similarity to another CDR3 sequence within the group. Node sizes represent overall abundance in samples and nodes are colored based on the relative ratio between their percent in NR (red) samples *vs*. in R samples (blue). Relative ratio = (average % in NR)/(average % in R + average % in NR). The shared clonotype’s node within Group 1 or 2 was colored purple and labeled with its corresponding TCRβ CDR3 a.a. sequence. **(F)** TCR repertoire overlap in R and NR TIL samples. The overlap coefficient was calculated in a pairwise manner between each sample using repOverlap function in immunarch package and the resulting matrix is plotted.

Since CD8 T cells were essential for treatment efficacy and individual mice harbor a stochastically generated TCR repertoire *via* random V(D)J recombination, we next examined whether the differences in the TCR repertoire of CD8 TILs may correlate to the heterogeneous responses to anti-PD-L1 in different tumor-bearing mice. To delineate whether and how CD8 T cells differ between R and NR, we sequenced CD8 T cells from 4 responder (R1-R4) and 4 non-responder mice (NR1-NR4) (**Figure 3A**, left) employing coupled single-cell TCR V(D)J sequencing and scRNA-seq to link the unique TCR clonotypes with corresponding transcriptomes of individual T cells (**Figure 3A**, right). We isolated and sequenced splenic CD8 T cells from 4 R and 4 NR mice, designated as RSP1-RSP4 *vs*. NRSP1-NRSP4, as well as CD8 TILs, designated as RTIL1-RTIL4 *vs*. NRTIL1-NRTIL4 (see details in **Supplemental Table 1**).

While splenic CD8 T cells showed little clonal expansion, all 8 CD8 TIL samples demonstrated clonal expansion regardless of R or NR state (**Figure 3B**). We showed the top 10 TCR clonotypes (including VDJ usage and CDR3 sequences of TCRα and TCRβ) and their abundance in each sample (**Supplemental Table 2**). Of note, there were few TCR clonotypes shared between samples, denoted by colored pie slices (**Figure 3B**), although most of them were only found in up to two samples in high abundance (**Figure 3B**, **Supplemental Table 3**). Overall, we observed a similar level of clonal expansion in CD8 TILs of both R and NR samples, consistent with increased CD8 TILs in both R and NR compared to control samples (**Figure 2C**).

To better delineate the relative abundance of all TCR clones in the entire repertoire, we employed repClonality function of immunarch package to analyze our single-cell TCR sequencing data. We found that the vast majority of TCR clonotypes in splenic CD8 T cells showed very low relative abundance, meaning that these clones did not undergo clonal expansion (**Supplemental Figure 3A**), although some TCR clones in NRSP2 spleen sample showed a modest level of clonal expansion (**Supplemental Figure 3A**), consistent with our own analysis (**Figure 3B**). In contrast, TCR clonotypes in CD8 TIL samples had a very high level of relative abundance (**Supplemental Figure 3A**), demonstrating clonal expansion regardless of R or NR state.

Despite the few shared clonotypes between samples, the top TCR clonotypes (abundance >1% of a given sample) appeared mutually exclusive between R and NR TIL samples (**Figure 3C**). Moreover, a vast majority of these top TCR clonotypes were only identified in one mouse, and ~8% of these clonotypes were present in more than one mouse (**Supplemental Figure 3B**). To determine whether top clonotypes in R group are also observed in NR group at a similar frequency and vice versa, we performed a Fisher’s Exact test and found that R top clonotypes were much more frequently observed in R than in NR (*P*<0.0001), and vice versa, thereby confirming the mutual exclusivity of R *vs*. NR clonotypes (**Supplemental Figure 3C**). Consistently, when we analyzed TCRα or TCRβ CDR3 sequences separately, the top TCRα and TCRβ CDR3 sequences appeared mutually exclusive between R *vs*. NR TILs, with only a few exceptions (**Supplemental Figures 3D–E**). Hence, our results reveal a highly individualized anti-tumor immune response upon anti-PD-L1 treatment in both R and NR that also exhibited mutual exclusivity of TCR clonotypes.

Although the top expanded TCR clonotypes did not have identical CDR3 amino acid (a.a.) sequences, it remains possible that TCR clonotypes may share similarity within R or NR group or between the two groups. To better explain why CD8 TILs in R inhibited tumor growth more effectively, we hypothesized that R TILs might have more TCR clonotypes shared in certain strong, anti-tumor specificity groups. To test our hypothesis, we employed the GLIPH algorithm (44) to define whether the TCR clonotypes of the TIL samples were sharing specificities (for antigen binding), despite not having identical CDR3 a.a. sequences. GLIPH analysis identified the top 20 groups of TCRβ CDR3 sequences in 8 TIL samples based on overall abundance (**Supplemental Table 4**). However, none of the 20 groups suggested a common specificity to either R or NR, because most of them were largely mutually exclusive in individual mice (**Figures 3D, E**; **Supplemental Figure 3F**; **Supplemental Table 4**). The only groups shared between R and NR are Group 1, 2, 5, 8 and 17, whose census CDR3β a.a. sequences were shown (**Supplemental Figure 3G**). Altogether, these data are consistent with the notion that a highly individualized anti-tumor immune response develops in different R or NR hosts.

### Repertoire differences in splenic CD8 T cells and CD8 TILs detected by single-cell TCR-seq

Given that the top expanded TCR clonotypes were almost mutually exclusive between R and NR TIL, we next asked whether the entire sequenced TCR repertoires overlap between any samples. Our data showed that CD8 TILs and splenic CD8 T cells from the same mouse showed a relatively high level of overlapping TCR clonotypes (**Supplemental Figure 3H**); however, the samples from different mice exhibited an extremely low level of overlap (**Supplemental Figure 3H**). We also quantified the similarity in TIL TCR repertoires within R and NR groups and found little overlap between any samples, except for RTIL3 and RTIL4 as these two samples contained low abundant overlapping TCR clones (**Figure 3F**; **Supplemental Table 3**).

To obtain a higher-level overview of whether TCR determinants are associated with anti-PD-L1 response, we assessed the usage of germline Vα-Jα or Vβ-Jβ gene segments in individual mice that might provide a broader view of how TCR repertoires vary between individual mice and between R *vs*. NR group. For each of the 16 samples sequenced for TCR, we included a heatmap for the usage of Vα-Jα gene segments (**Supplemental Figures 3I–L**) or Vβ-Jβ gene segments (**Supplemental Figures 3M, N**). Splenic samples appeared to display a more even distribution of different V or J gene segments (**Supplemental Figures 3K, L, N**), whereas TIL samples clearly showed a preferential usage of certain V or J gene segments (**Supplemental Figures 3I, J, M**), consistent with the expansion of distinct TCR clonotypes in TILs. However, we did not identify any preferential usage of certain gene segments that were shared in R or NR TILs (**Supplemental Figures 3I, J, M**). We performed K-means clustering based on TCRβ V gene usage in all 16 samples sequenced and found that spleen samples tend to cluster together, whereas TIL samples did not separate into different clusters according to R *vs*. NR state (**Supplemental Figure 3O**). These data suggest that the usage of Vα-Jα or Vβ-Jβ gene segments in TIL samples appears to be distinct in individual mice and does not correlate to R or NR state.

### Both R and NR CD8 TILs were activated and found to be in all activation clusters

**Figure 4:**
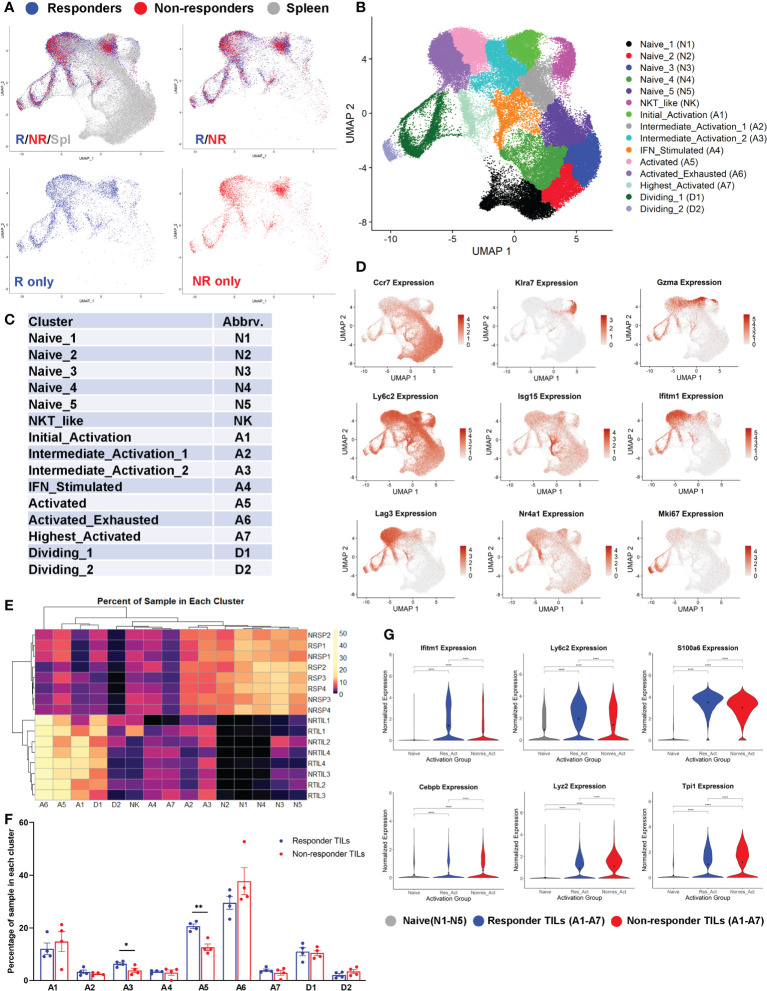
Both responder (R) and non-responder (NR) CD8 TILs were activated. **(A)** UMAP plots of different samples shown as superimposed for spleen (gray), R TILs (blue) and NR TILs (red) or superimposed for R TILs (blue) and NR TILs (red) or separately for R TILs (blue) and NR TILs (red). Gene expression data of total 74260 cells from all 16 samples (RSP1-4, NRSP1-4, RTIL1-4, NRTIL1-4) were analyzed using Seurat version 4.0.2 and clustered using UMAP (see details in **Supplemental Table 1**). **(B)** Cells from 16 different samples were clustered using UMAP and 15 different clusters were labeled based on gene expression. **(C)** Cluster abbreviations are listed to be referenced in other plots. **(D)** UMAPs showing the normalized expression of 9 genes representative of different clusters (gray=little to no expression; red=high expression). **(E)** Heatmap showing the percent of each sample residing in each cluster of the UMAP. Samples and cluster names were ordered by unsupervised clustering. **(F)** Graphical summary of the percent of R or NR CD8 TILs residing in each cluster of A1-A7 and D1-D2. Data were presented as mean ± SEM. Statistical significance was calculated with unpaired t test (*, *P*<0.05; **, *P*<0.01). **(G)** Violin plots showing the normalized expression of representative genes upregulated in R or NR activated TILs (residing in one of the 7 activated clusters: A1-A7) compared to naïve T cells (residing in N1-N5 clusters). Naïve; naïve clusters in all samples, Res_Act; activated clusters in R TIL samples, Nonres_Act; activated clusters in NR TIL samples. Black dot indicates the mean of each group. Different groups were compared using one-way ANOVA followed by Tukey’s multiple comparisons test (****, *P*<0.0001).

We performed 5’ scRNA-seq on eight CD8 TIL samples including 4 R (RTIL1-RTIL4) *vs*. 4 NR (NRTIL1-NRTIL4) and eight splenic CD8 T cell samples including 4R (RSP1-RSP4) *vs*. 4 NR (NRSP1-NRSP4) (see details in **Supplemental Table 1**). RNA expression data from all 16 samples (in total 74260 cells) were analyzed using Seurat version 4.0.2, and samples were plotted into one UMAP, shown superimposed for spleen, R-TIL and NR-TIL, or superimposed or separately for R-TIL and NR-TIL (**Figure 4A**). Cells were grouped by unsupervised clustering (**Figure 4B**) and differentially expressed genes in each cluster were identified by FindMarkers function in Seurat and 15 different clusters were defined based on the expression of representative genes (**Figures 4C, D**; **Supplemental Figures 4A–C**). For instance, *Isg15* is expressed highest in A4, *Nr4a1*, *Nr4a3*, *Xcl1*, *Ccl4* and *Ifng* in A7, and *Mki67* in D1 and D2 clusters (**Figure 4D**; **Supplemental Figure 4A**). While spleen cells predominated in naïve clusters (N1-N5) of the UMAP, both R and NR CD8 TILs mainly occupied the activation clusters (**Figures 4A, B**), suggesting that not only R but also NR TILs managed to reach different activation states. Consistently, R and NR TILs upregulated nearly all of the same genes when compared with naïve T cells, albeit to different extents (**Supplemental Table 5**).

We examined the percent of each sample distributed in individual clusters and found that the percent of CD8 T cells in cluster A3 or A5 was significantly higher in R than NR TIL samples (**Figures 4E, F**), whereas splenic samples were more prevalently distributed in N1-N5 naïve clusters (**Figure 4E**). When compared with naïve CD8 T cells, we observed only a few genes more significantly upregulated in R TILs (e.g., *Ifitm1*) and a few others in NR TILs (e.g., *Lyz2*) (**Figure 4G**; **Supplemental Table 5**). When samples were separated by sequencing cohort, we found that these genes were still differentially expressed between R and NR TILs within each cohort (**Supplemental Figure 4D**). We conclude that both R and NR TILs were activated and found to be in all activation clusters, although R TILs more frequently distributed to A3 and A5 clusters.

### Top TCR clonotypes of CD8 TILs differentially occupy activation clusters in R *vs*. NR

**Figure 5:**
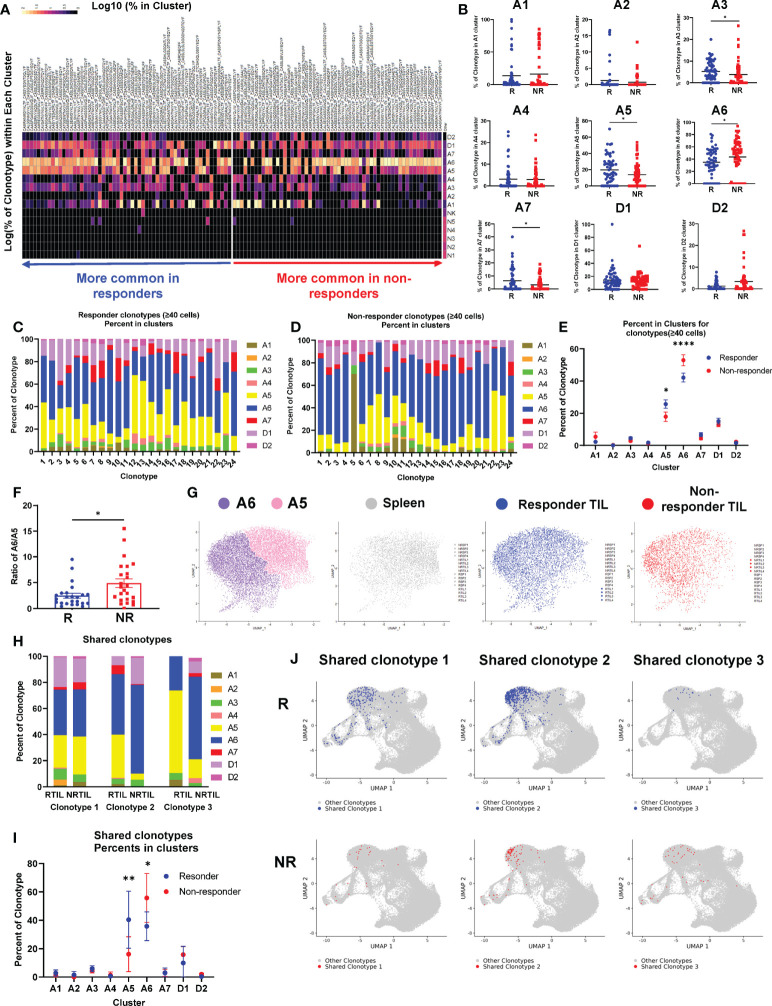
Top TCR clonotypes of CD8 TILs differentially occupy activation clusters in responders (R) *vs*. non-responders (NR). **(A)** Heatmap of the log10 percent of top TCR clonotypes (n=58 per group, >0.65% in R TIL or >1% in NR TIL samples) residing in each cluster of the UMAP. **(B)** Quantification of percent of R (blue) or NR (red) top TCR clonotypes residing in activated clusters (A1-A7) and dividing clusters (D1-D2) of the UMAP. Each dot represents a clonotype and the black line indicates the mean. Groups were compared using unpaired t-test with Mann-Whitney U test correction for non-parametric data (*, *P*<0.05). **(C, D)** All top TCR clonotypes from R **(C)** or NR **(D)** containing at least 40 cells are shown as bar graphs with the percent of clonotype in each cluster (A1-A7, D1-D2) broken down. **(E)** Quantification of percent of R clonotypes (blue) in **(C)** and NR clonotypes (red) in **(D)** in each cluster of the UMAP. Data were presented as mean ± SEM. Statistical significance was calculated with two-way ANOVA followed by Sidak’s multiple comparisons test (*, *P*<0.05; ****, *P*<0.0001). **(F)** Ratio of the percent in A6 *vs*. the percent in A5 (% in A6/% in A5) for a given TCR clonotype in R (blue) or NR (red). Each dot represents a clonotype. Data were presented as mean ± SEM. Statistical significance was calculated with unpaired t test (*, *P*<0.05). **(G)** Cells in A5 and A6 clusters were extracted and plotted into one UMAP showing A5 (pink) and A6 (purple) clusters from all 16 samples, or from R TILs (blue), NR TILs (red) or splenic CD8 T cells (gray), respectively. **(H–J)** Three TCR clonotypes shared between R and NR CD8 TILs were identified that contained at least 20 cells in a given group (**Supplemental Table 3**). **(H)** The percent of each of the 3 shared clonotypes in each cluster (A1-A7, D1-D2) in R *vs*. NR TIL samples. **(I)** Quantification of the percent of 3 shared clonotypes in each cluster (A1-A7, D1-D2) in R *vs*. NR CD8 TIL samples. Data were presented as mean ± SEM. Statistical significance was calculated with two-way ANOVA followed by Sidak’s multiple comparisons test (*P<0.05; **P<0.01). **(J)** UMAP of the CD8 T cells harboring the shared TCR clonotypes. Cells with a given TCR clonotype are shown for R TILs (blue) or NR TILs (red), over cells with all other TCR clonotypes from all samples (gray).

Next, we tested whether the CD8 TILs with top TCR clonotypes would be differentially activated between R and NR. Based on identical TCRα and TCRβ CDR3 a.a. sequences, CD8 T cells were grouped into TCR clonotypes, then clonotypes were sorted according to abundance in R *vs*. abundance in NR. We compared the top TIL TCR clonotypes to “Other” (defined as clonotypes <1% of a splenic sample) for the percentage of cells identified in each cluster within a given clonotype. R top TIL TCR clonotypes were significantly more prevalent in clusters A3, A5, and A7, whereas NR ones were significantly more prevalent in cluster A6 (**Figures 5A, B**). To account for individual mouse variation, we analyzed cluster distribution by comparing TCR clonotypes within each cohort of R *vs*. NR. Differences were evaluated using two-way ANOVA for Progression group (R *vs*. NR) and for Cohort. Variations by Progression group were statistically significant as indicated on the right of each plot for cluster A3 and A5 (*, *P*<0.05), A6 (**, *P*<0.01) and A7 (***, *P*<0.001), while the differences in each cluster appeared to vary by cohort (**Supplemental Figure 5A**).

To further corroborate our observation, we examined all R and NR clonotypes that contained at least 40 cells for their distribution in different clusters (**Figures 5C, D**). The percent of both R and NR clonotypes was highest in A6 cluster meaning that most of cells distributed into this cluster; furthermore, the percentage of NR clonotypes was significantly higher in cluster A6 than R ones (**Figure 5E**). In contrast, the percent of R clonotypes was higher in cluster A5 than NR ones (**Figure 5E**). The ratio of cluster A6 to A5 is much higher for NR clonotypes than R ones (**Figure 5F**), suggesting that a given NR CD8 TIL is more likely to be present in A6 cluster. To better delineate the differences between cluster A5 and A6, we pooled all sequenced CD8 T cells including spleen and TIL samples that were plotted into one UMAP or shown separately for Spleen, R TIL or NR TIL (**Figure 5G**). Because no salient difference was observed between R and NR spleen (**Supplemental Figure 5B**), splenic samples were pooled and presented together (**Figure 5G**, gray). Splenic CD8 T cells or NR TILs more frequently occupied A5 or A6 cluster, respectively. In contrast, R TILs distributed to both A5 and A6 clusters comparably (**Figure 5G**, blue).

We then asked whether the same TCR clonotype would behave differently in the TME of R *vs*. NR. Although very few TCR clonotypes were shared between R and NR TIL samples, we were able to identify three shared TCR clonotypes that contained at least 20 cells in a given group (**Supplemental Table 3**). We calculated the percent of cells in all clusters for each of three shared clonotypes and found that the same TCR clonotype behaved differently in R *vs*. NR TIL samples (**Figure 5H**). Statistical analysis of the pooled data from three shared clonotypes showed that, CD8 T cells, despite having the same TCR clonotypes, enriched significantly more in cluster A5 when found in R than in NR (**Figures 5I, J**). In contrast, CD8 T cells with the same TCR clonotypes distributed to cluster A6 more frequently when found in NR than in R (**Figures 5I, J**). Taken together, we conclude that NR CD8 TILs were skewed to A6 cluster, whereas R CD8 TILs appeared to occupy A6 and A5 cluster comparably.

### TILs with top TCR clonotypes exhibited differentially activated genes in R *vs*. NR

**Figure 6:**
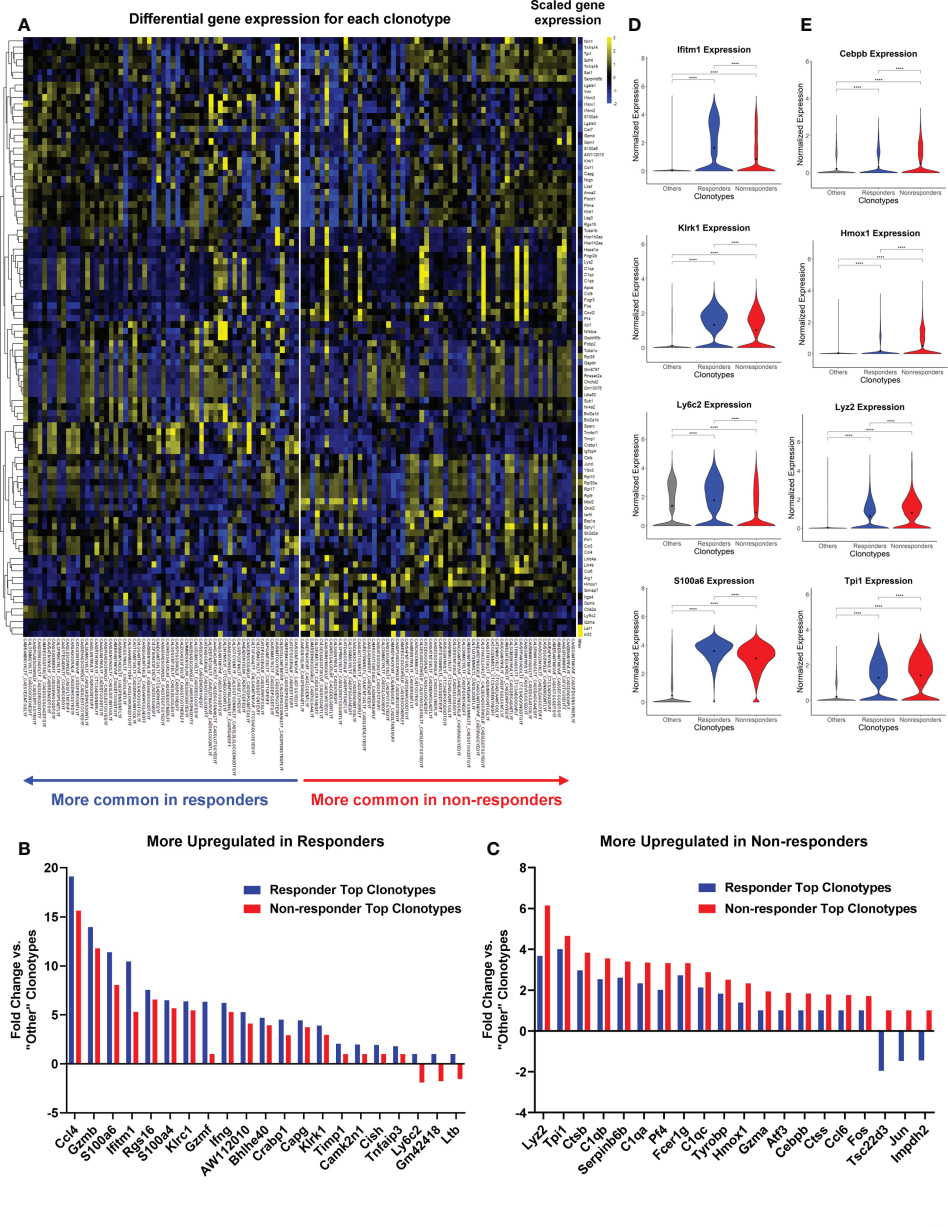
Top TCR clonotypes of TILs exhibited differentially activated genes in responders (R) *vs*. non-responders (NR). All cells from top TCR clonotypes (>0.65% in R TIL and >1% in NR TIL, n=58 per group) were compared against all cells with clonotypes <1% of all spleen samples (“Other”) using Seurat’s FindMarkers function. **(A)** Heatmap showing the gene expression where values are the average of scaled expression for all cells with a given clonotype. Genes were filtered for those differentially expressed in R and NR clonotypes (≥0.4 difference between R fold change and NR fold change). Color intensity is scaled by row. **(B, C)** Fold changes of representative genes more upregulated in R **(B)** or NR top clonotypes **(C)** *vs*. “Other” clonotypes (≥0.6 difference between R fold change and NR fold change). See **Supplemental Table 6** for the list of genes with fold changes and *P*-value. **(D, E)** Violin plots showing the expression of representative genes upregulated in R **(D)** or NR **(E)** top clonotypes compared to “Other” clonotypes. Black dot indicates the mean of each group. Different groups were compared using one-way ANOVA followed by Tukey’s multiple comparisons test (****P<0.0001).

To further uncover the differences between R and NR CD8 TILs, we focused on the TILs harboring top TCR clonotypes and performed two different DEG comparisons: (I) R top TIL clonotypes (>0.65% of a R TIL sample) *vs*. “Other” clonotypes (<1% of a splenic sample), and (II) NR top TIL clonotypes (>1% of a NR TIL sample) *vs*. “Other” clonotypes. The heatmap showed the differential scaled gene expression of each top TCR clonotype of TIL samples *vs*. “Other” clonotypes, with clusters of differentially expressed genes in R *vs*. NR (**Figure 6A**). All of the differentially expressed genes for each top TCR clonotype were listed in **Supplemental File 1**. The most differentially expressed genes with their averaged fold changes and the most conservative p values calculated from each cohort were listed for comparison I and II (**Supplemental Table 6**).

We showed the fold changes of some representative genes that were either upregulated in R or NR (**Figures 6B, C**). R top clonotypes expressed higher levels of genes related to cytotoxic or effector functions of activated T cells, including *Ccl4*, *Gzmb*, and *Ifng* or genes involved in memory T cell function, including *Ly6c2* (49) (**Figure 6B**; **Supplemental Table 6**). Conversely, NR top clonotypes expressed much more transcription factors, *Fos and Jun*, than R ones (**Figure 6C**; **Supplemental Table 6**). NR top clonotypes also expressed higher levels of genes involved in immunosuppressive processes such as *Tsc22d3* that was shown to facilitate the generation of peripherally induced Tregs (50). Violin plots of normalized expression of representative genes in all cells consistently showed differing expression between R *vs*. NR top clonotypes (**Figures 6D, E**). We also separated cells by cohort and found that these genes were still differentially expressed between R and NR top clonotypes (**Supplemental Figures 5C, D**).

To directly compare top TCR clonotypes in R *vs*. NR, we performed two additional DEG comparisons: (III) R top clonotypes *vs*. NR top clonotypes, and (IV) NR top clonotypes *vs*. R top clonotypes. The data from comparison III and IV including averaged fold changes and the most conservative p values calculated from each cohort were listed in **Supplemental Table 7**. Taken together, NR top clonotypes, while expressing many of the same genes as their R counterparts, appear to be limited in the magnitude of activation and become exhausted. In contrast, R top clonotypes appear to achieve a higher magnitude of activation and acquire stronger cytotoxic ability.

### Validation of single-cell sequencing data

**Figure 7:**
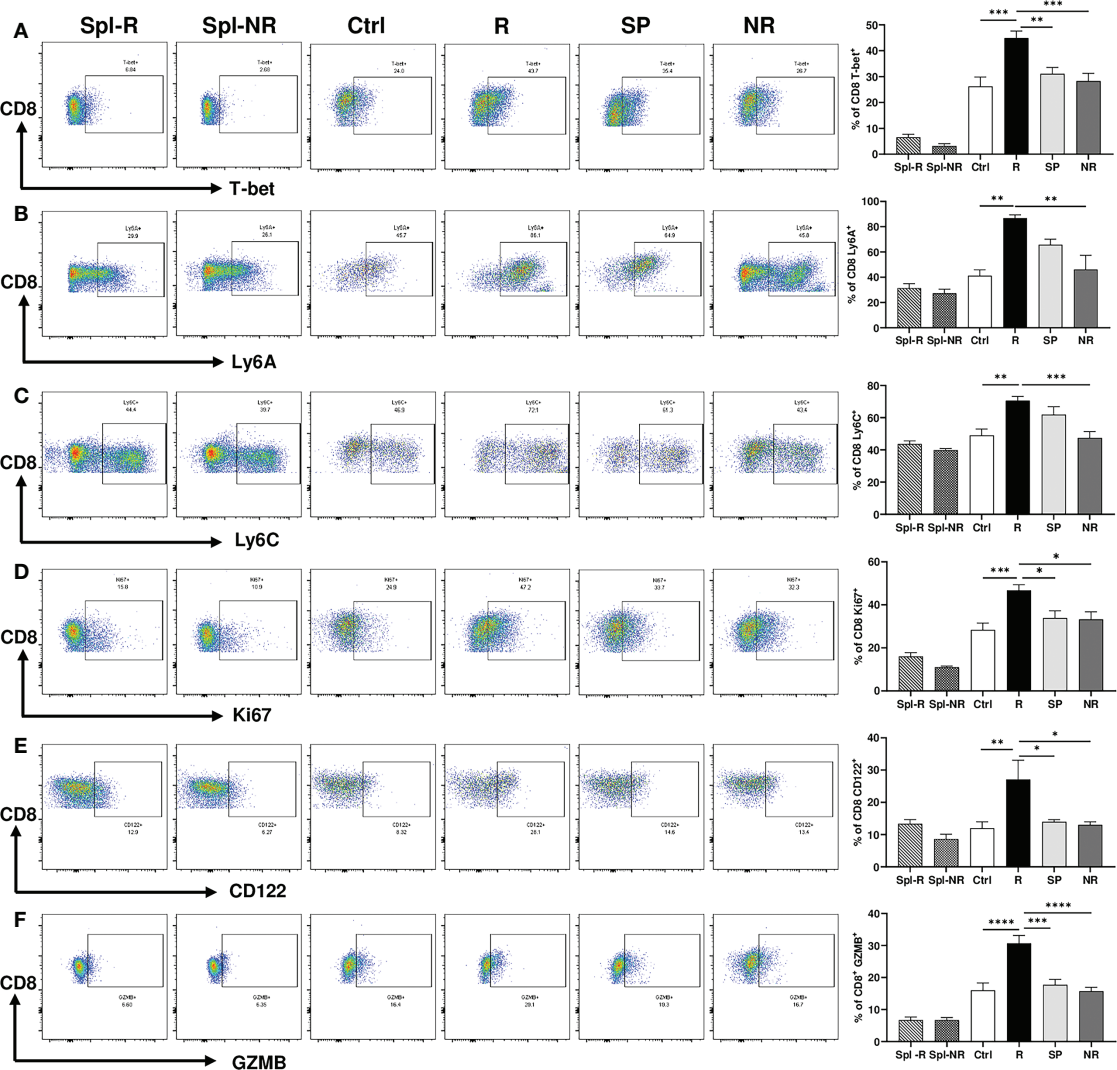
Activation markers differentially expressed between responder (R) and non-responder (NR) CD8 TILs. **(A–F)** Representative flow plots and percentages of CD8 T cells expressing T-bet **(A)**, Ly6A **(B)**, Ly6C **(C)**, Ki67 **(D)**, CD122 **(E)**, GZMB **(F)** in spleens from R (Spl-R) or NR (Spl-NR) mice, or in tumors from control (Ctrl) or anti-PD-L1 treated recipients including R, slow progressors (SP), and NR. Data were presented as mean ± SEM (Spl-R: n=10; Spl-NR: n=12; Ctrl: n=7; R: n=10; SP: n=8; and NR: n=12). Statistical significance was calculated with one-way ANOVA followed by Tukey’s multiple comparisons test (*P<0.05; **P<0.01; ***P<0.001; ****P<0.0001).

**Figure 8:**
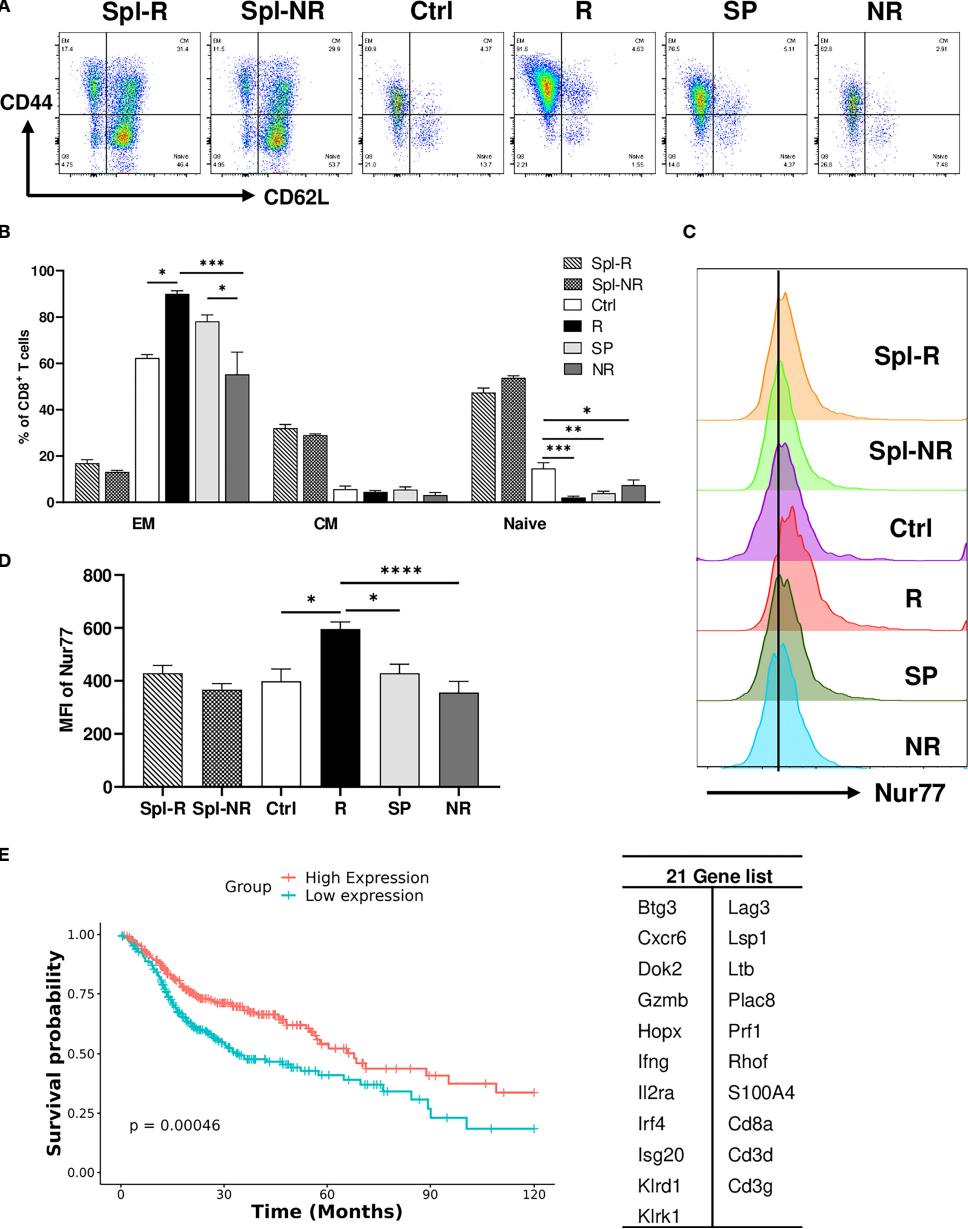
Validation of single-cell sequencing data. **(A, B)** Representative flow plots **(A)** and percentages **(B)** of different memory CD8 T cell populations in Spl-R, Spl-NR, Ctrl, R, SP and NR groups. EM: effector memory (CD44^+^CD62L^−^), CM: central memory (CD44^+^CD62L^+^) and naïve (CD44^−^CD62L^+^). **(C, D)** Representative flow plots **(C)** and mean fluorescence intensity (MFI) **(D)** of Nur77 (Nr4a1) expression in CD8 T cells from Spl-R, Spl-NR, Ctrl, R, SP, and NR groups. Data were presented as mean ± SEM (Spl-R: n=10; Spl-NR: n=12; Ctrl: n=7; R: n=10; SP: n=8; and NR: n=12). Statistical significance was calculated with one-way ANOVA followed by Tukey’s multiple comparisons test (*P<0.05; **P<0.01; ***P<0.001; ****P<0.0001). **(E)** Genes more upregulated in responder top TCR clonotypes (n=21, right) were used to score HNSCC patients (n=502). Patient data are derived from HNSCC TCGA Pan-Cancer Atlas RNA sequencing data (RNA_Seq_v2_mRNA_median_all_sample_Zscores) (cBioPortal). Scores for the 21 genes were calculated as the Mean of Zscore transformed data of mRNA expression of genes, and then patients were grouped into high-expression (if score > the median score) or low-expression (if score ≤ the median score) and analyzed for survival.

To validate our scRNA-seq data, we performed flow cytometry experiments to examine CD8 T cell markers of activation and memory. We found that R CD8 TILs expressed more T-bet, Ly6A, Ly6C, Ki67, CD122, and GZMB than control or NR CD8 TILs (**Figures 7A–F**). We also examined other markers including KLRG1, CD127, CD69, CD25, Eomes, NKG2D, CD278, CD244, and CD49d, whose expression level did not differ significantly between R *vs*. NR CD8 TILs (data not shown). To assess the different populations of memory CD8 T cells, we performed flow cytometry to evaluate CD44 and CD62L expression. The percentage of effector memory CD8 T cells (CD44^+^CD62L^−^) was significantly higher in R CD8 TILs than either control or NR CD8 TILs (**Figures 8A, B**), whereas the percentage of naïve CD8 T cells (CD44^−^CD62L^+^) was significantly higher in control CD8 TILs than anti-PD-L1 treated groups including R, SP and NR (**Figures 8A, B**). Flow cytometry analysis also showed that the expression of Nur77 (a.k.a. NR4A1) was significantly higher in R CD8 TILs than control, SP or NR group (**Figures 8C, D**). Overall, our flow cytometry analysis validated our findings of scRNA-seq that R CD8 TILs appear to be more activated.

To further validate our findings in human HNSCC patients, we employed the HNSCC patient survival and RNA-sequencing data from the TCGA Pan-Cancer Atlas (cBioPortal) to test if the genes more upregulated in responder top clonotypes can predict better survival in HNSCC patients. We scored each patient for the expression of 21 genes and grouped patients into high expression *vs*. low expression group. We found that patients with high expression of the 21-Gene-Signature exhibited significantly better survival (*P*<0.001) compared to patients with low expression (**Figure 8E**). These data suggest that the genes more upregulated in responder top clonotypes may serve as predictive markers for better survival in HNSCC patients.

## Discussion

We employed a unique HNSCC mouse model to study the underlying factors of differential responses to anti-PD-L1 treatment. We showed that (1): tumor-bearing recipients diverged into R, SP, or NR upon anti-PD-L1 treatment (2); Responses to anti-PD-L1 absolutely required CD8 T-cells and correlated positively with effector polyfunctionality of CD8 TILs (3); A similar extent of clonal expansion was observed in the TCR repertoires of CD8 TILs regardless of R *vs*. NR status (4); The top expanded TCR clonotypes were almost mutually exclusive between R and NR, demonstrating preferential expansion of distinct TCR clonotypes in R *vs*. NR. Furthermore, majority of top TCR clonotypes were detected in only one recipient, indicating a highly individualized anti-tumor immune response against the same tumor cell line (5); R and NR CD8 TILs did not differ greatly in transcriptional activation except that R or NR CD8 TILs more frequently occupied distinct activation clusters (6); individual markers were identified to correlate with R or NR status in CD8 TILs with top expanded TCR clonotypes. We conclude that stochastic differences in TIL TCR repertoire and distinct activation states of top TCR clonotypes might contribute to differential anti-PD-L1 responses.

ICI responses in cancer patients are highly heterogeneous. Prior studies often focus on tumor-intrinsic mechanisms associated with heterogeneous anti-tumor responses (4, 51). Nevertheless, with confounding variables investigated together (e.g., different oncogenic mutations), many aspects of the tumor and TME may vary; it becomes difficult to dissect contributions of the immune system. A223 tumor may serve as a useful model in which we could largely minimize the effects of host genetic background and tumor-intrinsic factors, potentially allowing us to discover host immune-intrinsic factors that govern heterogeneous ICI responses in different individuals.

In line with prior studies (52–56), we found that the percentage of CD8 T cells was significantly increased in R compared to control and CD8 T cells were required for therapeutic effects in this model. The percentage of CD8 T cells was also significantly increased in SP and NR, albeit to a lesser extent than R, suggesting that CD8 T cells also infiltrated tumors in SP and NR. Nevertheless, CD8 TILs were much more activated in R *vs*. NR and SP given the much higher percentage of IFN-γ^+^TNF-α^+^ double producers in R (**Figure 2B, D**). Ideally, we should sequence the IFN-γ^+^TNF-α^+^ double producers for their TCR clonotypes and transcriptomes; however, the procedure of intracellular cytokine staining is not compatible with 10×Genomics single-cell sequencing platform. Hence, future studies are needed to optimize the experimental approaches to further investigate distinct effector populations (e.g., single *vs*. double producers).

To better understand the differences between CD8 TILs in R *vs*. NR, we performed single-cell TCR-seq with 10×Genomics platform to delineate TCR clonotype differences. Unexpectedly, we found that CD8 TILs underwent a similar level of TCR clonal expansion in R and NR, which suggest that CD8 TILs were activated to expand in both R and NR. Notably, we discovered that the top expanded TCR clonotypes were almost mutually exclusive between R and NR CD8 TILs. These data demonstrate that, although CD8 TIL TCR clonotypes exhibited a similar degree of clonal expansion, completely different TCR clonotypes were expanded in R *vs*. NR. This observation indicates that R and NR CD8 TILs might mount drastically different responses by employing distinct TCRs against the same A223 tumor cell line. While clonal expansion implicates that these top TCR clonotypes are likely tumor-specific, bystander T cells could also undergo clonal expansion in TME. To define antigen specificities of these TCR clonotypes, we will need to isolate individual TCR sequences and test their specificities against tumor cells or tumor antigens either *in vitro* by co-culturing T cells and tumor cells or *in vivo* using transplanted tumor model, which warrants future studies. Results from such studies may help to explain why the TCR clonal expansion is observed in both responders and non-responders. It would also require an antigen-specific system and substantial future studies to address whether top TCR clonotypes within the R group result in the successful ICI responses and provide advantageous recognition of tumor antigens over top TCR clonotypes within the NR group. In line with our observation, prior studies showed that many different TCR clonotypes can react to the same MHC/peptide antigens (57, 58). Hence, we suggest that stochastic differences in TIL TCR repertoire may be one of the several factors that might underlie differential responses to ICI treatment. Of course, this notion does not exclude the contribution of tumor-intrinsic factors, including TMB, tumor immunogenicity, PD-L1 expression or others, to differential ICI responses (7, 8, 11, 59–61); nevertheless, our study may offer a new perspective to test whether stochastic differences in TCR repertoire contribute to variable ICI responses in different individuals.

We also performed single-cell RNA-seq coupled with single-cell TCR-seq so that we could examine transcriptional differences in different TCR clonotypes. Unexpectedly, we found that both R and NR CD8 TILs reached all activation states regardless of response status. When analyzed as a whole, R and NR CD8 TILs did not show significant transcriptional differences except that R CD8 TILs more frequently distributed to cluster A3 and A5. When transcriptomes coupled with TCR clonotypes, our results suggest two scenarios for successful anti-tumor immune responses in R: (1) top expanded TIL TCR clonotypes have a higher chance to reach the highest activation cluster A7 in R, which expressed the highest level of *Ifng*, *Nr4a1*, *Nr4a3*, *Ccl4* and *Xcl1*; (2) top expanded TIL TCR clonotypes appeared to occupy both A6 and A5 clusters in R but were significantly skewed to A6 cluster in NR. To better understand this observation, we compared the genes differentially expressed between A5 *vs*. A6 cluster (**Supplemental File 2**). Of note, both A5 and A6 clusters contained activated T cells that expressed much higher levels of T cell activation markers such as *Klrc1*, *Klrk1*, *Nkg7*, *Icos*, and *Pdcd1*, compared to naive clusters. Activated CD8 T cells in A6 cluster expressed a higher level of checkpoint (e.g., *Lag3*, *Havcr2*, *Ctla4*) or effector molecules (*Gzmd*, *Gzme*, *Tnfrsf9*, *Prf1*, *Tnfrsf4*). In contrast, activated CD8 T cells in A5 cluster expressed a higher level of *Hspa1a* and *Hspa1b*, which encode the two major stress-induced Hsp70 family members also called Hsp72. Prior studies showed that Hsp70 reduced T cell proliferation and T cell responses when stimulated with DCs (62), consistent with a lower expression level of effector or checkpoint molecules in A5 cluster. Moreover, both A5 and A6 clusters downregulated *Klf2* expression, with an even lower level of *Klf2* in A6 cluster. KLF2 has been shown to restrain T cell functions such as cytokine production (63). TCR engagement reduced *Klf2* transcription, the higher the affinity of TCR ligand is, the more reduction of *Klf2* occurs (64). Altogether, these studies collectively suggest that activated CD8 T cells in A5 cluster may express certain genes that limit their capacity or restrain their activation, which apparently benefit anti-tumor immunity. In line with this idea, prior study showed that *Hspa1a* gene was upregulated after anti-PD1 treatment in responder CD8 T cells in basal cell carcinoma patients (65).

The shared clonotype 1 was identified in R and NR TIL samples with a much higher frequency in R than NR. These data suggest that the presence of a single tumor-reactive TCR clonotype is insufficient to mediate therapeutic effects of anti-PD-L1, and a combination of multiple clonotypes may be needed for efficacy. We further hypothesize that different recipients harbor intrinsic differences in their TCR repertoires that likely affect the chance of mounting an effective ICI response (1). For instance, differences in TCR repertoire could alter the frequency of tumor-reactive clones or the optimal composition of such clones mediating ICI responses. In this regard, our studies show that the A223 model might be a very useful tool for studying differential ICI responses, due to its unique and inherent ability to elicit heterogeneous anti-tumor immunity. Our analysis of the shared TCR clonotypes suggests that a T cell’s TCR does not dictate the activation states it can reach. For example, the shared TCR clonotype 2 occupied both A5 and A6 clusters in R but was predominantly confined to A6 cluster in NR, suggesting that the TCR alone was not sufficient to determine activation state since the cells with the same TCR clonotype differ greatly in R *vs*. NR. We postulate that, instead of TCR affinity alone driving differentiation into various activation states, the TME and other signals cooperate with TCR-based signaling to shape T cell differentiation into different activation states.

## Data availability statement

The data presented in the study are deposited in NCBI's Gene Expression Omnibus (GEO) and are accessible through GEO Series accession number GSE214348 (https://www.ncbi.nlm.nih.gov/geo/query/acc.cgi?acc=GSE214348).

## Ethics statement

This study was reviewed and animal work was approved by the Institutional Animal Care and Use Committee of University of Colorado Anschutz Medical Campus (AMC) (Aurora, CO) and University of Pittsburgh (Pittsburgh, PA).

## Author contributions

Conceptualization, JW. Formal analysis, JJ, RW and HG. Investigation, JJ, VP, SC, AK, MV, YK. Methodology, RW. Supervision, ZC. Writing, JW and JJ. All authors contributed to the article and approved the submitted version.

## Funding

This work was supported by UPMC Hillman Cancer Center startup fund to JW, and a THI pilot grant from University of Colorado Cancer Center to JW, NIH R01-DE027329, R01-DE028420 and R01DE031947 to JW. RW was supported by a NIH F31 fellowship (F31DE027854). SC was supported by a NIH T32 fellowship (T32CA174648).

## Acknowledgments

Single-cell sequencing work was performed by the Genomics and Microarray Core at the University of Colorado partially supported by NCI P30CA046934 and Genomics Research Core at the University of Pittsburgh. This study utilized the Department of Immunology and Microbiology Flow Cytometry Shared Resource Laboratory (University of Colorado AMC), and the Hillman Cancer Center Flow Cytometry Core Facility partially supported by NCI P30CA047904 for flow cytometry analysis. This research was supported in part by the University of Pittsburgh Center for Research Computing through the resources provided. We apologize to those whose work was not cited due to length restrictions.

## Conflict of interest

The authors declare that the research was conducted in the absence of any commercial or financial relationships that could be construed as a potential conflict of interest.

## Publisher’s note

All claims expressed in this article are solely those of the authors and do not necessarily represent those of their affiliated organizations, or those of the publisher, the editors and the reviewers. Any product that may be evaluated in this article, or claim that may be made by its manufacturer, is not guaranteed or endorsed by the publisher.

## Supplementary material

Supplementary Figure 1 Differential responses to anti-PD-L1 treatment in A223 tumor-bearing mice.

Supplementary Figure 2 The percentage of single producers of IFNγ and TNFα in CD8 TILs and the expression of checkpoint molecules on CD8 T cells.

Supplementary Figure 3 Top TCR clonotypes are mutually exclusive in responder (R) *vs*. non-responder (NR) CD8 TILs.

Supplementary Figure 4 Both responder (R) and non-responder (NR) CD8 TILs were activated.

Supplementary Figure 5 Top TCR clonotypes of CD8 TILs differentially occupy activation clusters in responder (R) TILs *vs*. non-responder (NR) TILs.

Supplemental File 1 data (clonotypes and gene names).

Supplemental File 2 A5 and A6 cluster comparison.

Supplemental Table 1 16 samples sequenced by Single-cell TCR and Single-cell RNA sequencing.

Supplemental Table 2 Detailed clonotype information for top 10 TCR clones in each sample.

Supplemental Table 3 TCR clonotypes shared between different TIL samples.

Supplemental Table 4 TCRβ CDR3 sequences of top 10 groups by GLIPH clustering.

Supplemental Table 5 Differential gene expression between responder TILs and naïve T cells or between non-responder TILs and naïve T cells.

Supplemental Table 6 Differential gene expression between either cells with Responder top clonotypes or cells with Non-responder top clonotypes, compared to Other clonotypes in spleens.

Supplemental Table 7 Differential gene expression between cells with Responder top TCR clonotypes and cells with Non-responder top TCR clonotypes.

Supplemental Table 8 Flow Cytometry Antibodies used in the study.

## Abbreviations

B6, C57BL/6; DEGs, Differentially Expressed Genes; FBS, Fetal Bovine Serum; GZB, Granzyme B; HNCs, Head and Neck Cancers; HNSCCs, Head and Neck Squamous Cell Carcinomas; HPV, Human Papillomavirus; ICIs, Immune Checkpoint Inhibitors; mAb, Monoclonal Antibody; PD1, Programmed Death 1; PD-L1, Programmed Death Ligand 1; RBC, Red Blood Cells; RCTV, Relative Change in Tumor Volume; RNASeq, RNA Sequencing; TME, Tumor Microenvironment; TILs, Tumor Infiltrating Lymphocytes; UMAP, Uniform Manifold Approximation and Projection; WT, Wild-Type.
