# Supplementary material for: Divergent outcomes of anti-PD-L1 treatment coupled with host-intrinsic differences in TCR repertoire and distinct T cell activation states in responding versus non-responding tumors
Source: Front Immunol. 2022 Oct 18;13:992630. doi: 10.3389/fimmu.2022.992630 (PMC9624473; doi:10.3389/fimmu.2022.992630)
Supplement: Supplementary Figure 1 — Differential responses to anti-PD-L1 treatment in A223 tumor-bearing mice. [file Presentation_1.pdf]

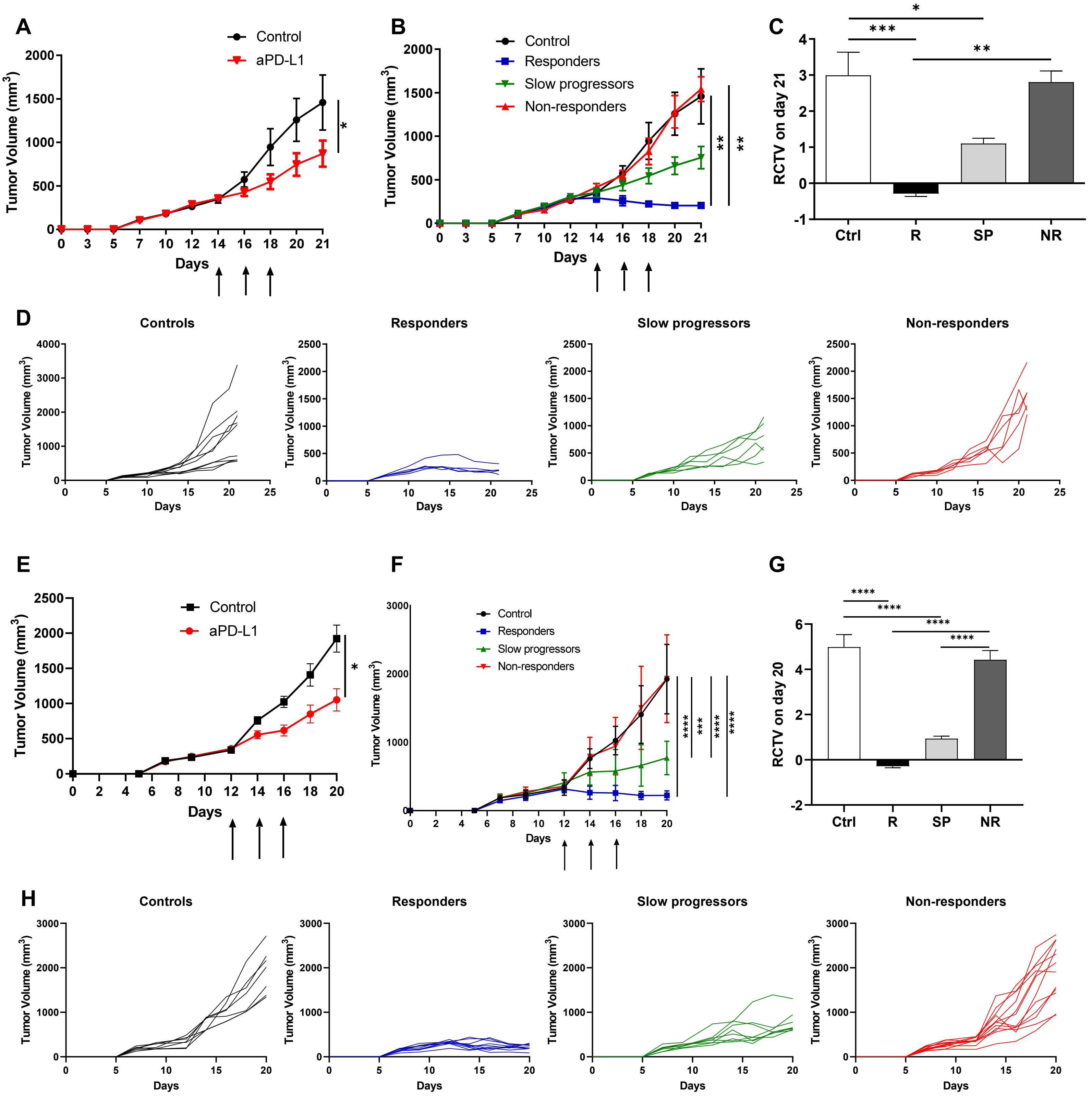

Supplemental Figure 1A-H

Supplemental Figure 1. Differential responses to anti-PD-L1 treatment in A223 tumor-bearing mice. (A-D) A223 tumor cells (1×10^5) were injected s.c. into one flank of WT B6 mice (n=30, 90% take rate). When the tumor size reached ~250-350mm^3, recipients were treated with control (n=9) or anti-PD-L1 mAb (n=17) for 3 times (2-day intervals, arrows indicate the days of treatment). Tumor growth was monitored for 21 days. **(A)** Overall tumor growth curves of control (n=9) and anti-PD-L1-treated (n=17) mice. **(B)** Tumor growth curves of control, R, SP and NR groups. According to relative change in tumor volume (RCTV) on day 21, anti-PD-L1 treated recipients diverged into R (n=5, RCTV<0), SP (n=6, 0<RCTV≤1.5) and NR (n=6, RCTV>1.5). **(C)** RCTV of control and different treatment groups (R, SP and NR). RCTV is calculated as the change in tumor volume (TV) from the start of treatment (day 14) to the endpoint date of the control group (day 21) divided by TV at day 14 $(TV_{day21}-TV_{day14})/TV_{day14}$. **(D)** Individual tumor growth curves of controls, R, SP, and NR groups. **(E-H)** A223 tumor cells (1×10^5) were injected s.c. into one flank of WT B6 mice (n=40, 90% take rate). Tumor-bearing recipients were treated with control (n=7) or anti-PD-L1 antibodies (n=30) as described above (arrows indicate the days of treatment). Tumor growth was monitored for 20 days. **(E)** Overall tumor growth curves of control (n=7) and anti-PD-L1-treated mice (n=30). **(F)** Tumor growth curves of control, R, SP and NR groups. According to RCTV on day 20, anti-PD-L1 treated recipients diverged into R (n=10, RCTV<0), SP (n=8, 0<RCTV≤1.5) and NR (n=12, RCTV>1.5). **(G)** RCTV of control and different treatment groups (R, SP and NR). RCTV is calculated as described above $(TV_{day20}-TV_{day12})/TV_{day12})$. **(H)** Individual tumor growth curves of controls, R, SP, and NR groups. Data were presented as mean ± SEM. Statistical significance was calculated with unpaired t test or one-way ANOVA followed by Tukey's multiple comparisons test (*, $P<0.05$; **, $P<0.01$; ***, $P<0.001$; ****, $P<0.0001$).

A

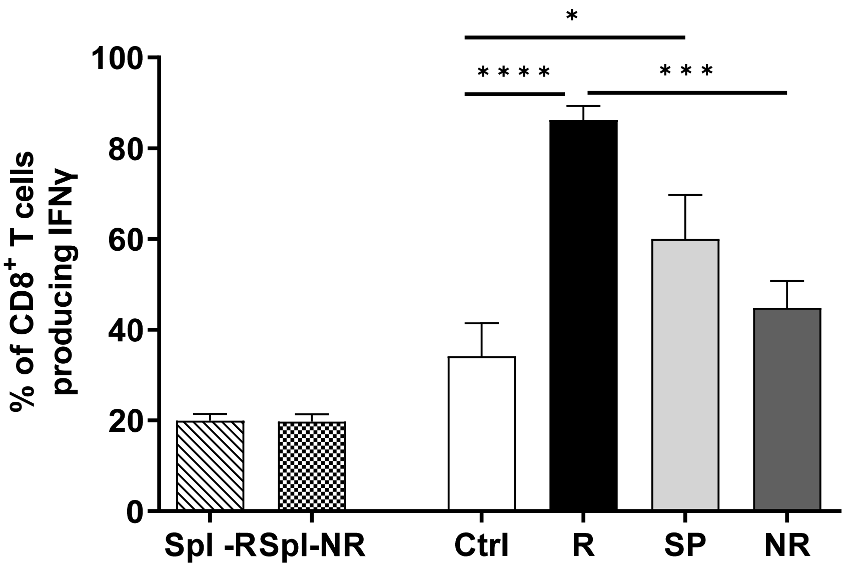

B

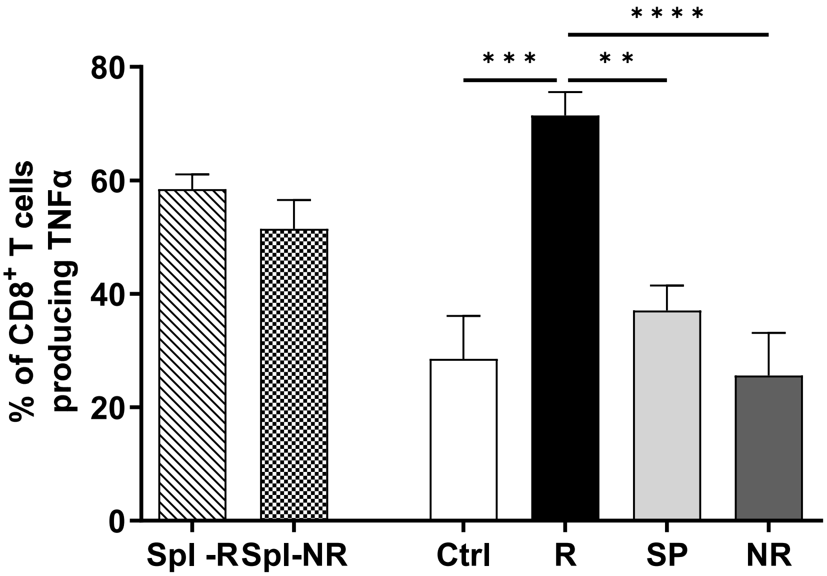

Supplemental Figure 2A,B

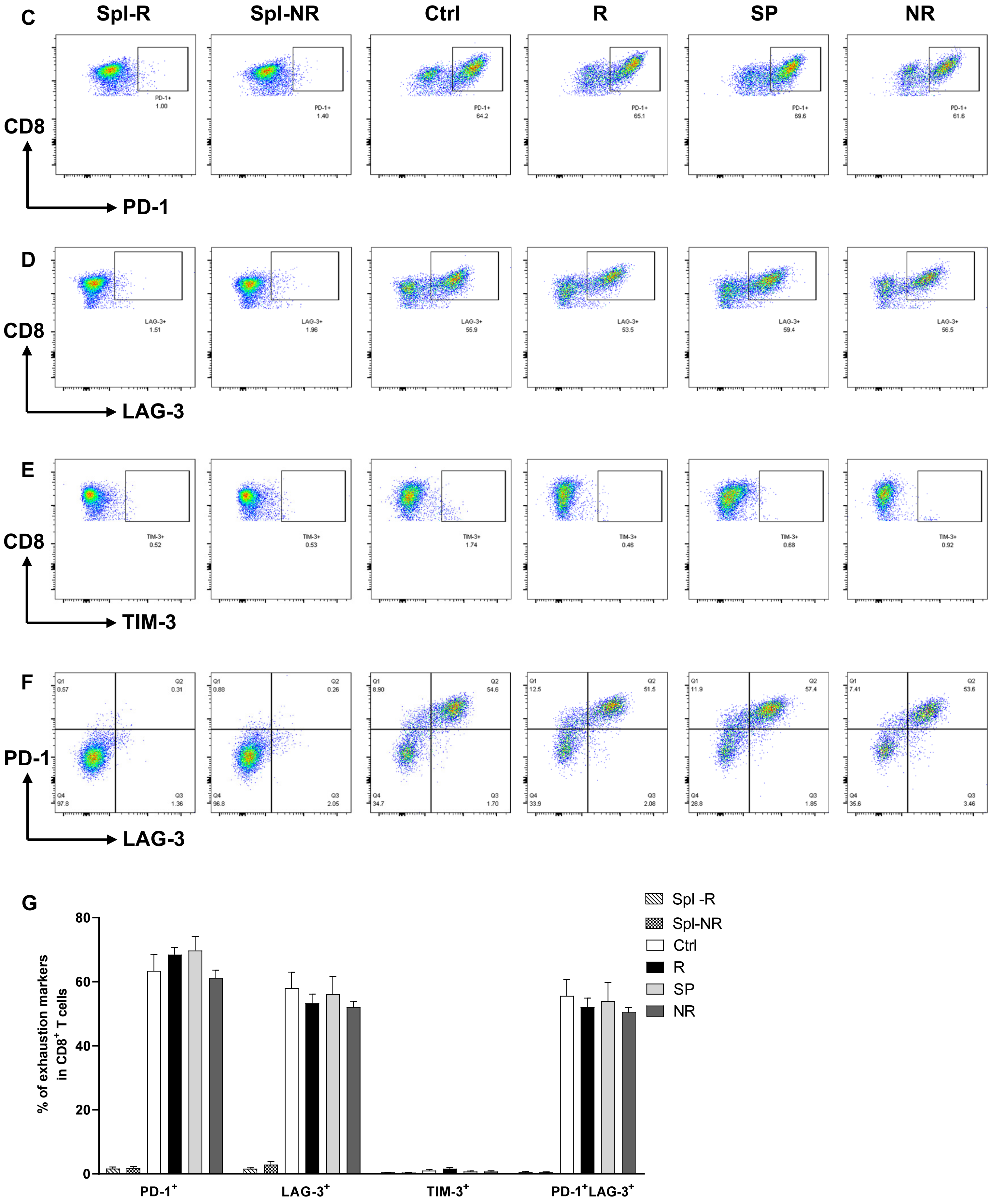

Supplemental Figure 2C-G

Supplemental Figure 2. Anti-PD-L1 treatment efficacy depends on CD8 T cells and did not affect the expression of checkpoint molecules on CD8 T cells. (A-B) Correlation of IFN-γ/TNF-α producing CD8 TILs with anti-PD-L1 responses. Data shown in the Figure 2D was analyzed to quantify the percentage of stimulated CD8 T cells producing IFNγ (IFNγ^+TNFα^- plus IFNγ^+TNFα^+) **(A)** or TNFα (TNFα^+IFNγ^- plus TNFα^+IFNγ^+) **(B)**. **(C-G)**Tumors and spleens were harvested from tumor-bearing recipients on the endpoint date of the control group and single-cell suspensions were analyzed by flow cytometry. Representative flow plots for expression of **(C)** programmed cell death protein 1 (PD-1), **(D)** lymphocyte-activation gene 3 (LAG-3), **(E)** T cell immunoglobulin and mucin domain 3 (TIM-3) and **(F)** co-expression of PD-1 and LAG-3 on CD8 T cells of spleen from R mice (Spl-R, n=7), spleen from NR mice (Spl-NR, n=6), control (Ctrl, n=7) and anti-PD-L1 treated recipients including R (n=7), SP (n=7) and NR (n=6). **(G)** Quantification of the percentage of CD8 T cells expressing checkpoint molecules in different groups. Data were presented as mean ± SEM. Representative data were shown from one cohort among three independently repeated cohorts (Figure 1A-H, Supplemental Figure 1A-D). Statistical significance was calculated with one-way ANOVA followed by Tukey's multiple comparisons test (*, P<0.05; **, P<0.01; ***, P<0.001; ****, P<0.0001).

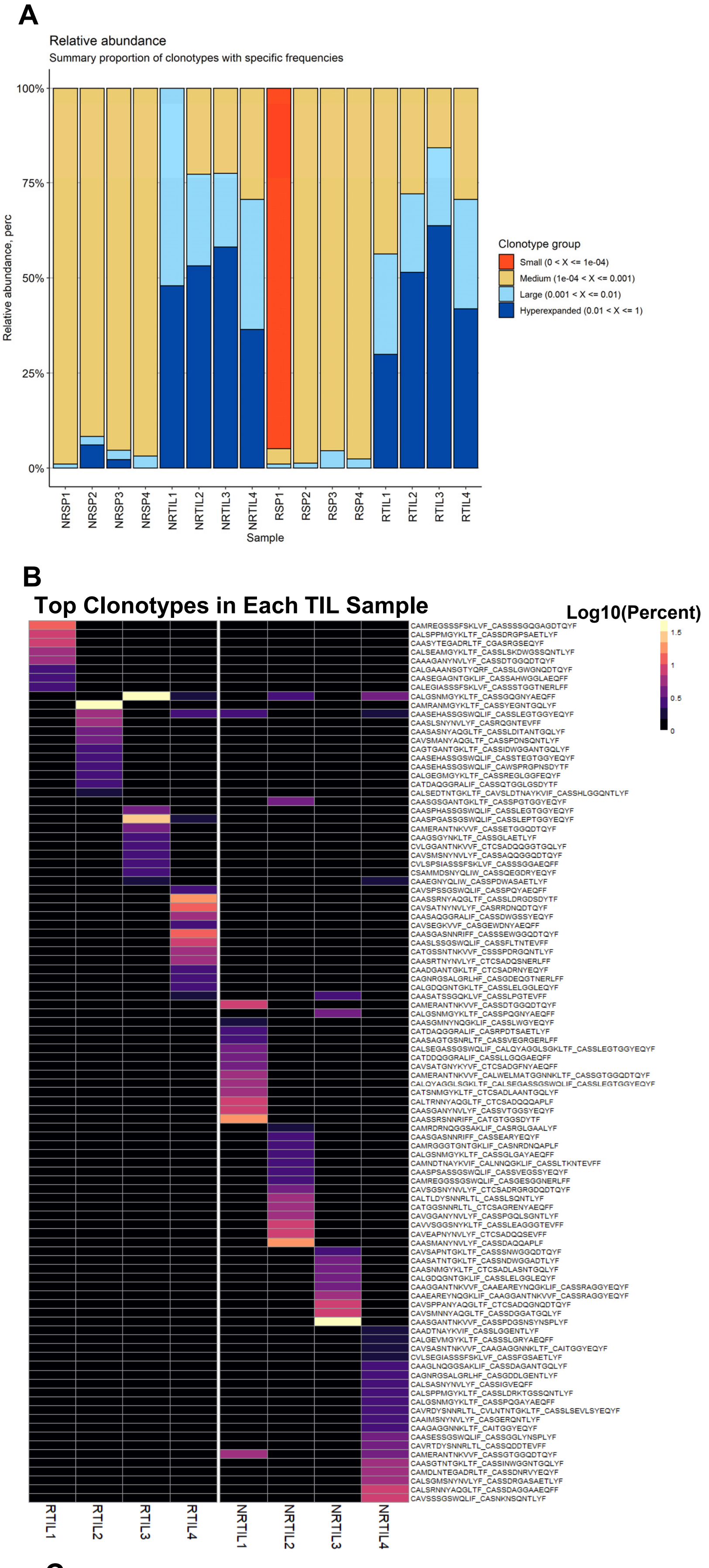

C

	In Responders	In Non-responders
Responder clonotypes	38	2
Non-responder clonotypes	5	55

****

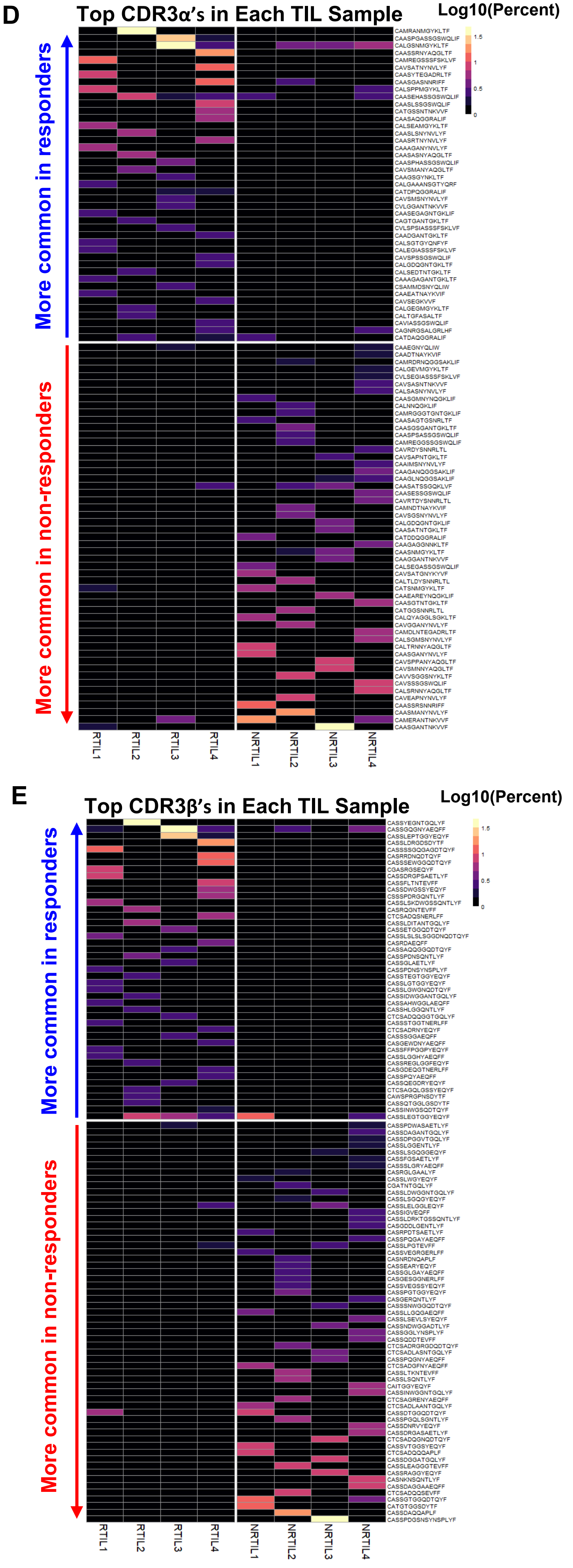

Supplemental Figure 3A-E

F

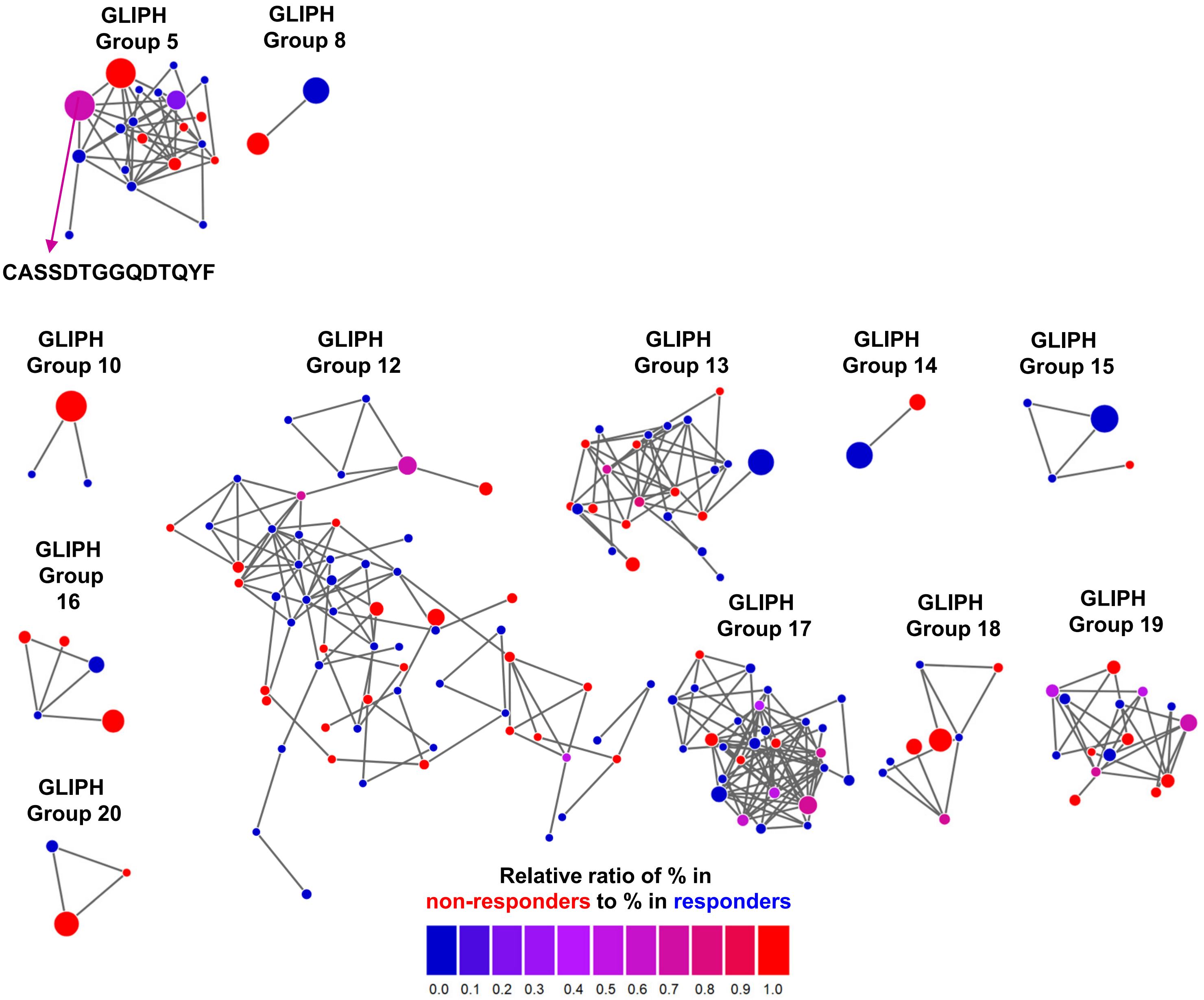

G

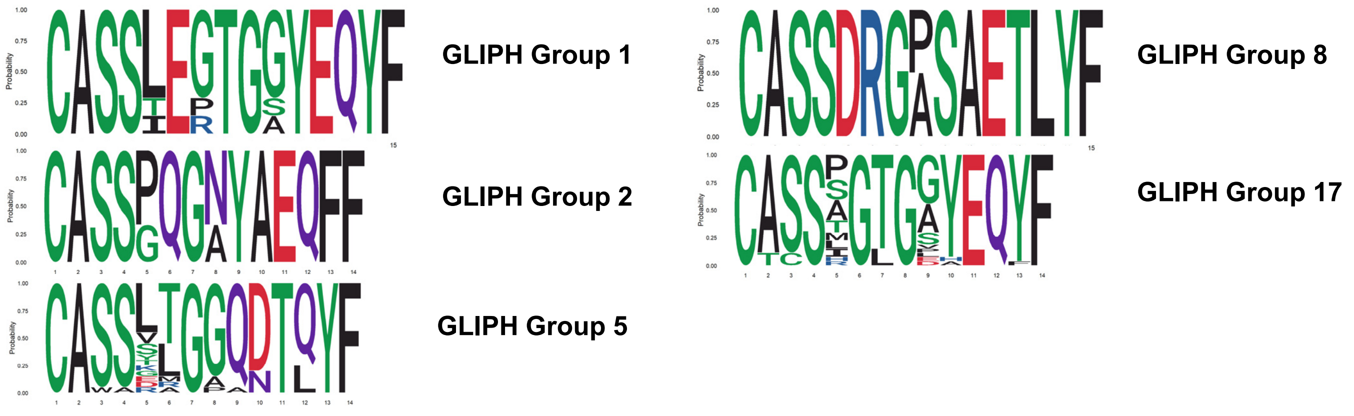

Supplemental Figure 3F-G

H

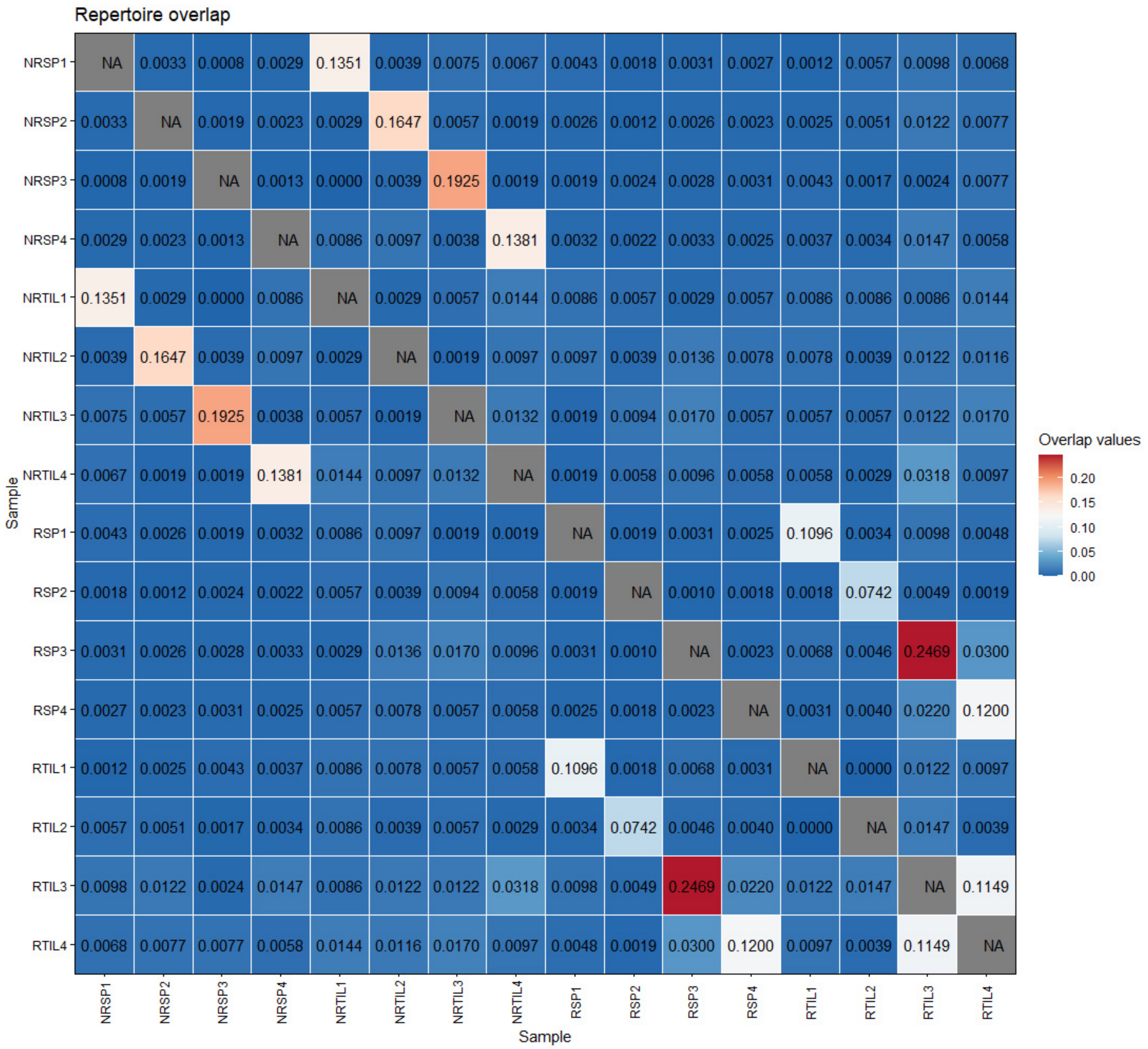

Supplemental Figure 3H

I

Responder TIL (TCRα V-J usage)

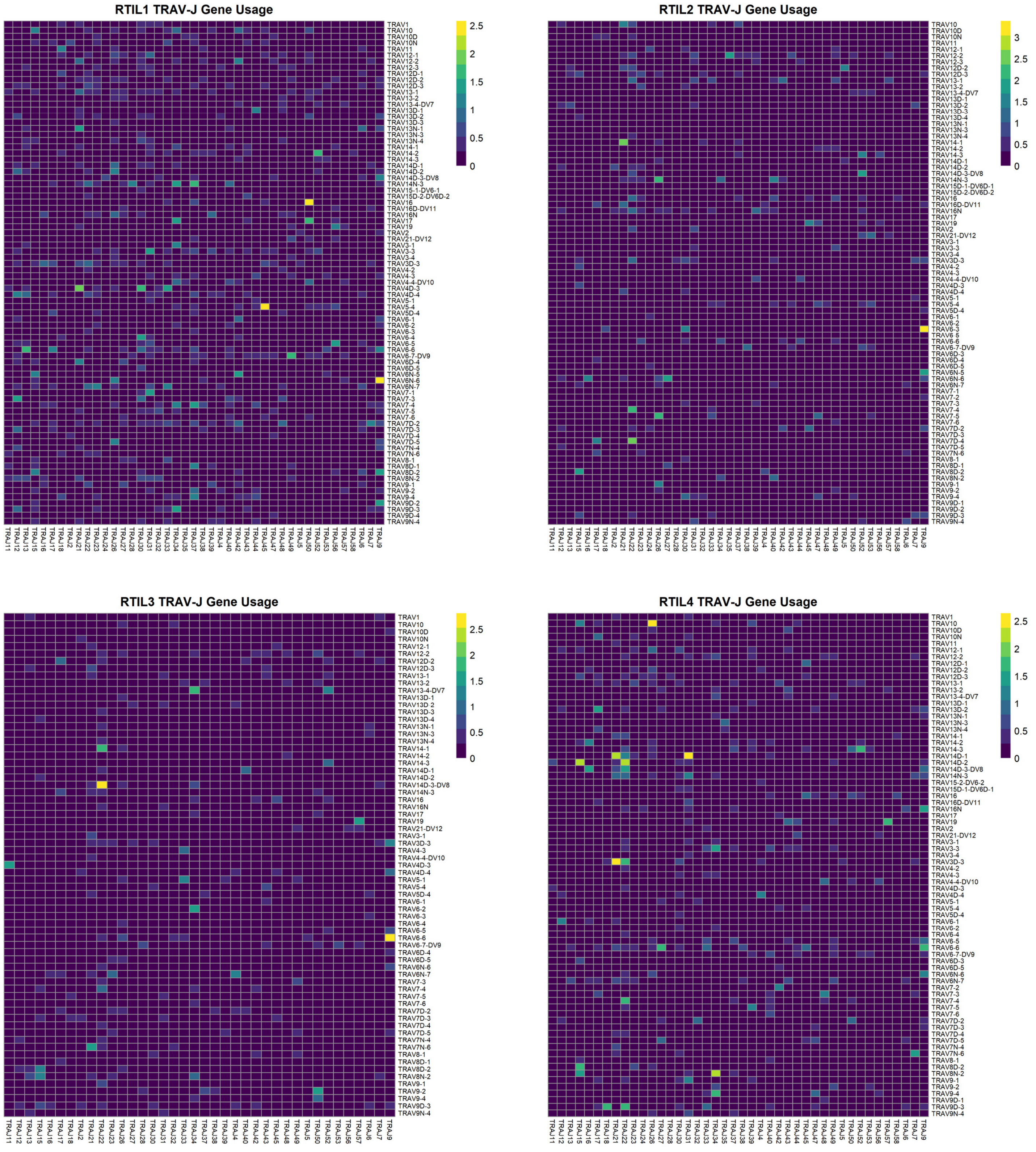

Supplemental Figure 3I

J

Non-responder TIL (TCRα V-J usage)

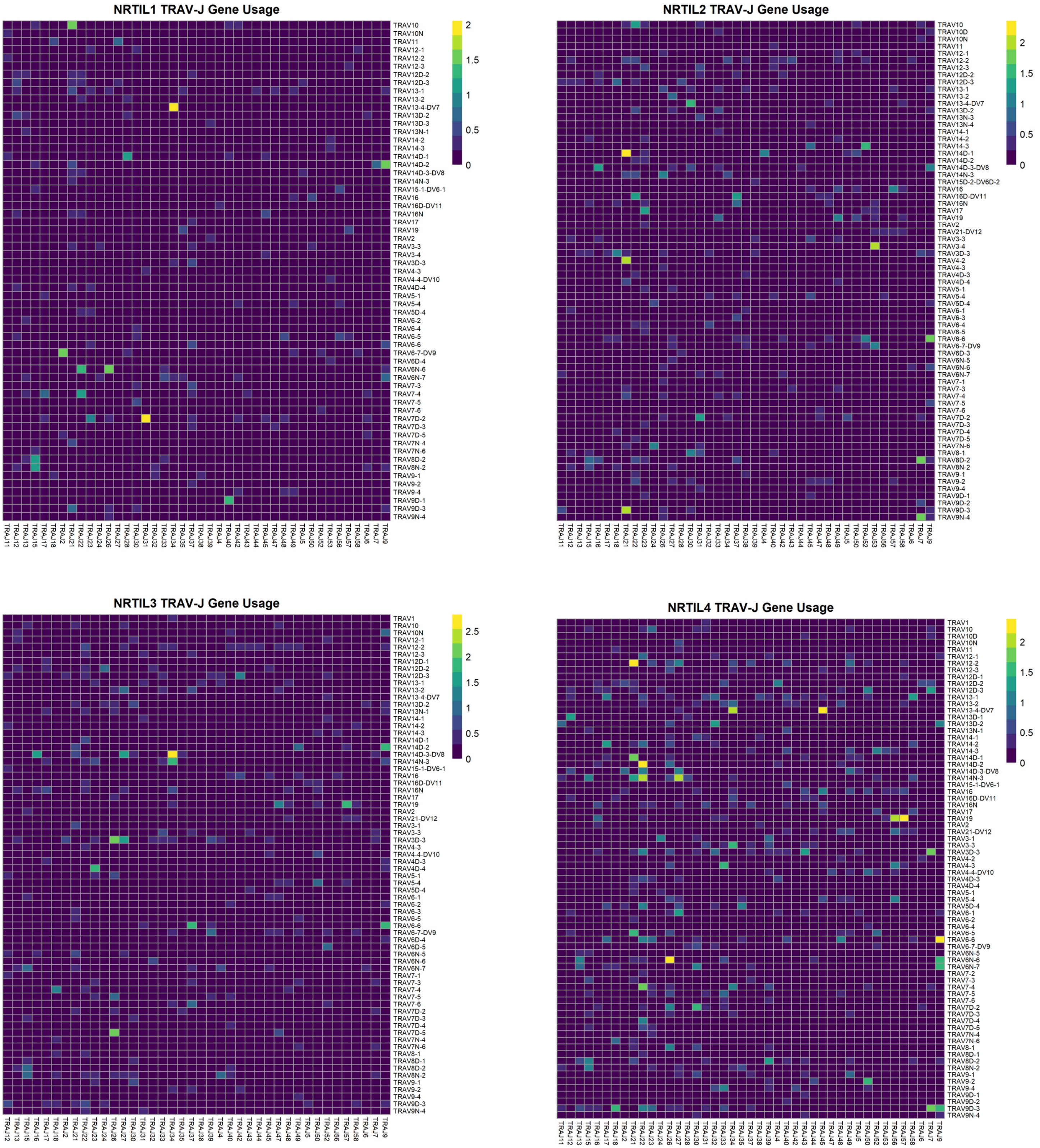

Supplemental Figure 3J

K

Responder Spleen (TCRα V-J usage)

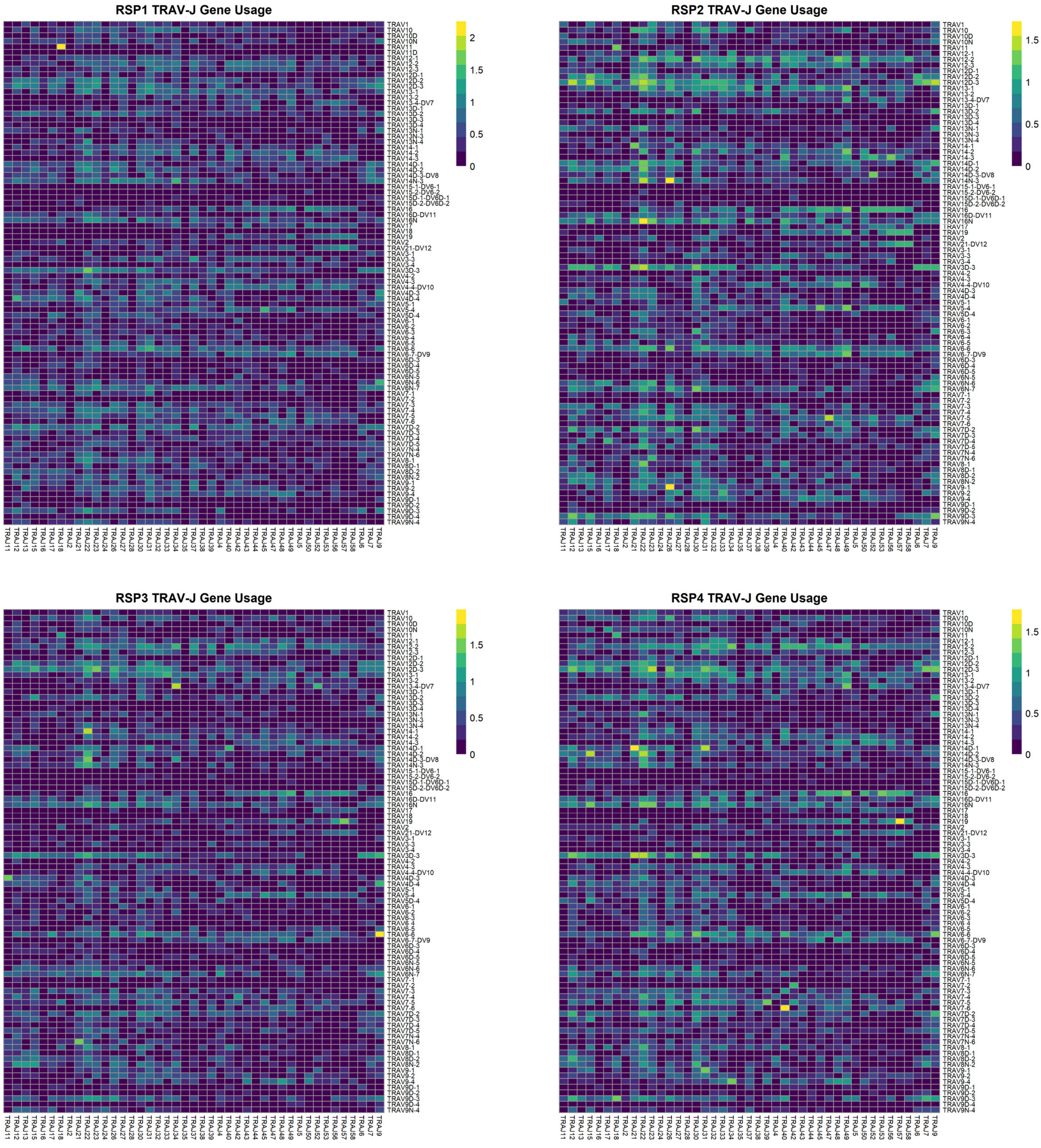

Supplemental Figure 3K

L

Non-responder Spleen (TCRα V-J usage)

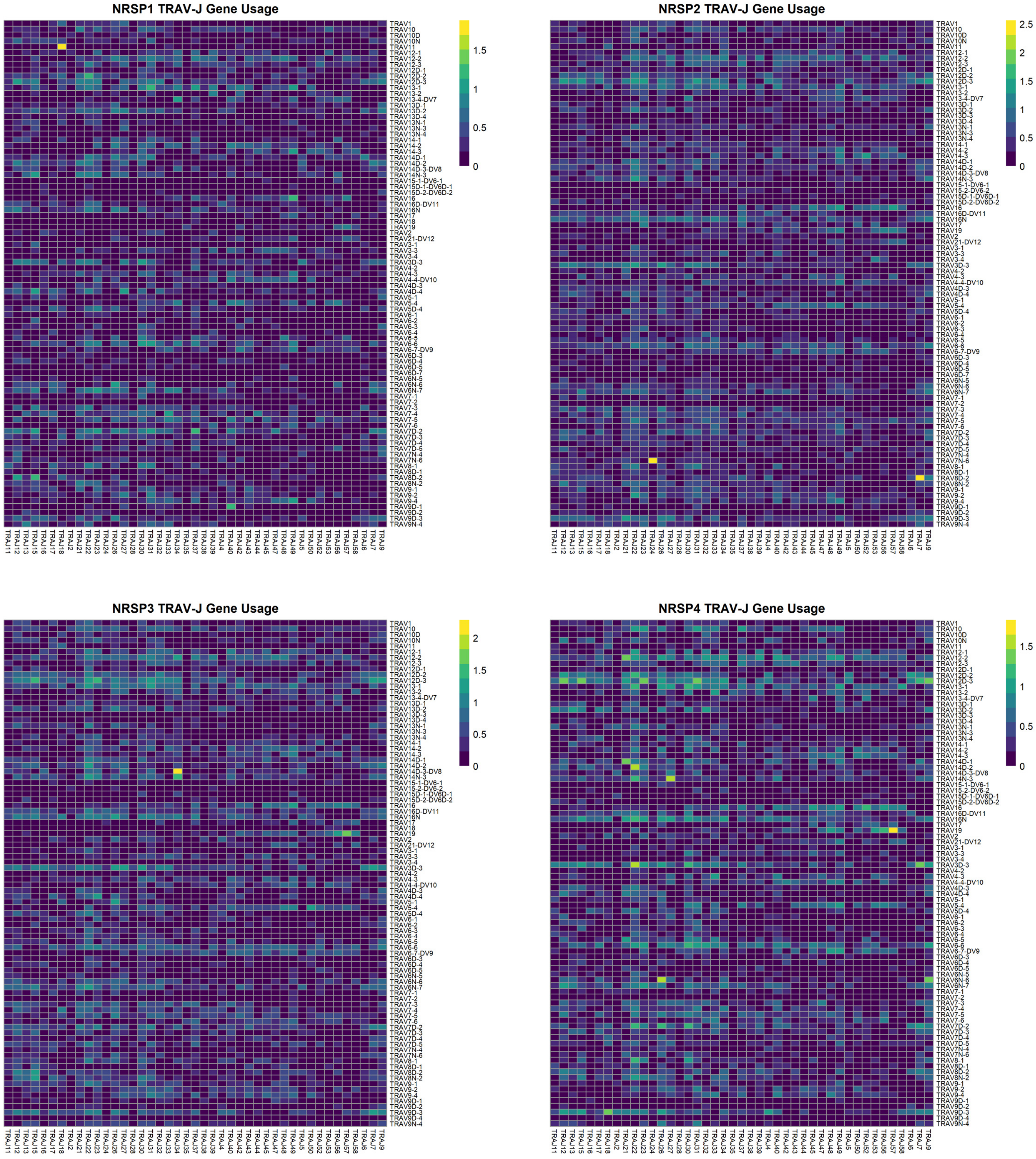

Supplemental Figure 3L

M

Responder and Non-Responder TIL (TCRβ V-J usage)

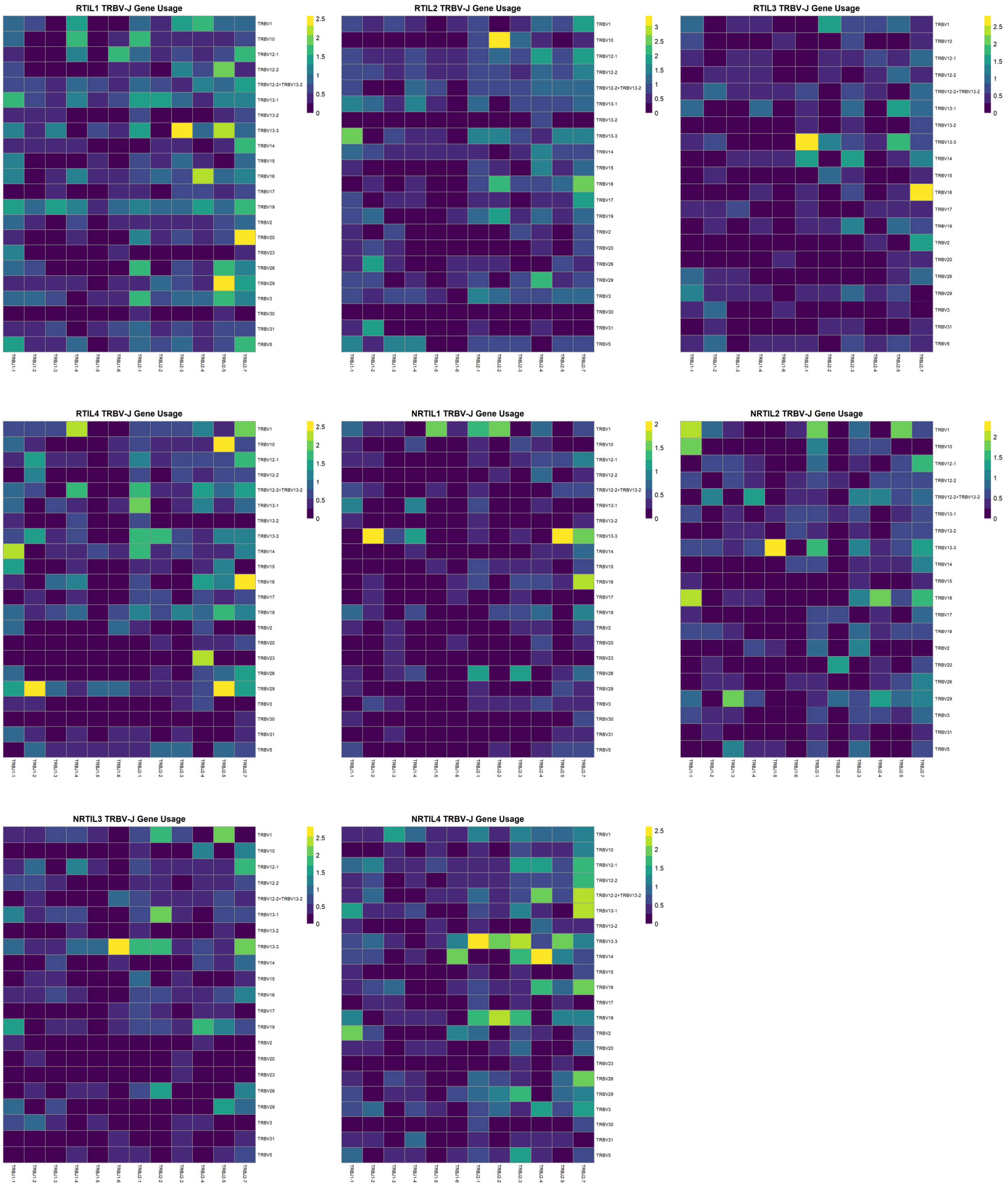

Supplemental Figure 3M

N **Responder and Non-Responder Spleen (TCRβ V-J usage)**

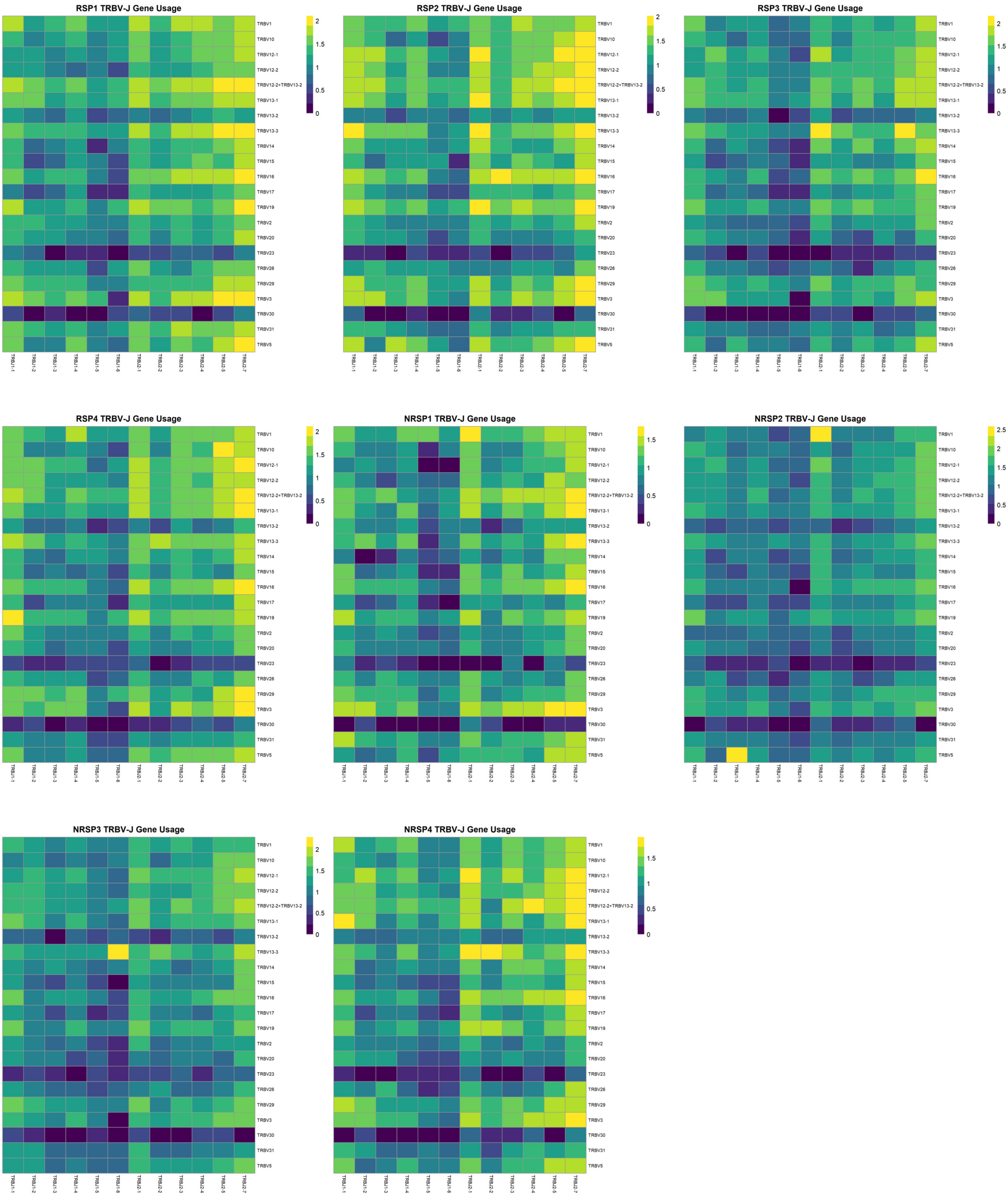

Supplemental Figure 3N

O

K-means clustering based on TRBV gene usage

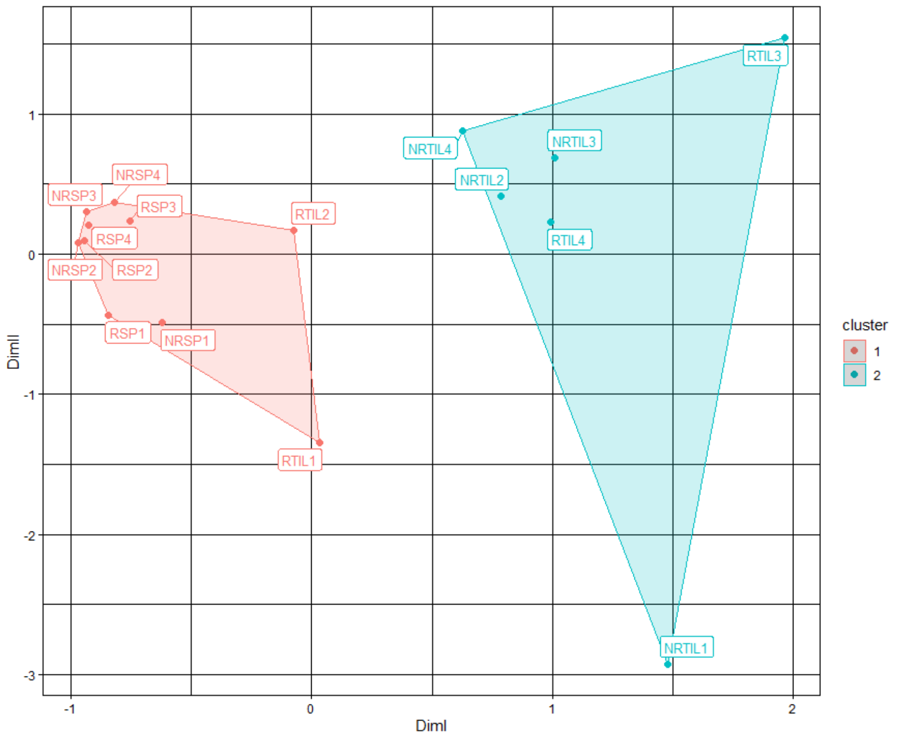

Supplemental Figure 3O

Supplemental Figure 3: Top TCRαβ clonotypes are mutually exclusive in responder (R) vs. non-responder (NR) CD8 TILs. (A) Relative abundance of TCR clonotypes in 16 sequenced samples including NR splenic CD8 (NRSP1-4), NR CD8 TIL (NRTIL1-4), R splenic CD8 (RSP1-4) and R CD8 TIL (RTIL1-4). The relative abundance of individual TCRβ clonotypes was calculated using repClonality function in immunarch package and TCR clonotypes were grouped accordingly as small, medium, large, and hyperexpanded clones. **(B)** Heatmap of top TCRαβ clonotypes (defined as >1% in any given sequenced TIL sample). The R top TCR clonotypes were sorted by descending abundance (%) in RTIL1, RTIL2, RTIL3, and RTIL4, sequentially. The NR top TCR clonotypes were sorted by ascending abundance in NRTIL1, NRTIL2, NRTIL3, and NRTIL4, sequentially. Cells with a given clonotype are colored according to the log10(percent) in each sample. **(C)** Statistical analysis for the mutual exclusivity of R vs. NR top TCR clonotypes. TCR clonotypes were grouped as R (n=40) or NR (n=60) clonotypes by their overall abundance in either group (See Figure 3C for R vs. NR clonotypes). 38 R clonotypes were observed only in R, whereas 2 R clonotypes were also observed in NR. 55 NR clonotypes were observed in NR only, whereas 5 NR clonotypes were also observed in R. Statistical significance was calculated by Fisher's Exact test to evaluate whether the clonotypes were exclusive to R or NR or shared (****, $P<0.0001$). **(D-E)** Heatmap of top TCRα **(D)** or TCRβ **(E)** CDR3 sequences (CDR3α or CDR3β) (>1% in any given sample) sorted by average abundance in R TILs vs. in NR TILs. **(F)** Network plots of GLIPH groups 5, 8, 10, 12, 13, 14, 15, 16, 17, 18, 19, 20. Each node represents a TCRβ CDR3 sequence in the group, and each line represents a global (thick line) or local (thin line) similarity to another CDR3 sequence. Node sizes represent overall abundance in samples and nodes are colored based on the relative ratio between their percent in NR (red) samples vs. their percent in R samples (blue) where purple is a sequence shared between R and NR samples. Relative ratio is calculated as (average % in NR) / (average % in R + average % in NR). **(G)** Consensus a.a. sequence for TCRβ CDR3 sequences in specificity groups shared between R and NR (Group 1, 2, 5, 8, 17) were plotted using ggseqlogo. **(H)** TCR repertoire overlap in different samples. The overlap coefficient was calculated in a pairwise manner between each sample using repOverlap function in immunarch package and the resulting matrix is plotted. **(I-L)** Heatmaps of the combinations of TCRα V-J gene usage in R TIL samples (RTIL1-4) **(I)**, NR TIL samples (NRTIL1-4) **(J)**, R spleen samples (RSP1-4) **(K)**, and NR spleen samples (NRSP1-4) **(L)**. **(M-N)** Heatmaps of the combinations of TCRβ V-J gene usage in R or NR TIL samples (RTIL1-4, NRTIL1-4) **(M)**, in R or NR spleen samples (RSP1-4, NRSP1-4) **(N)**. Cells with unique V-J combinations were colored according to log10(number of cells). **(O)** K-means clustering of 16 sequenced samples based on TCRβ V gene usage using geneUsageAnalysis function in immunarch package.

A

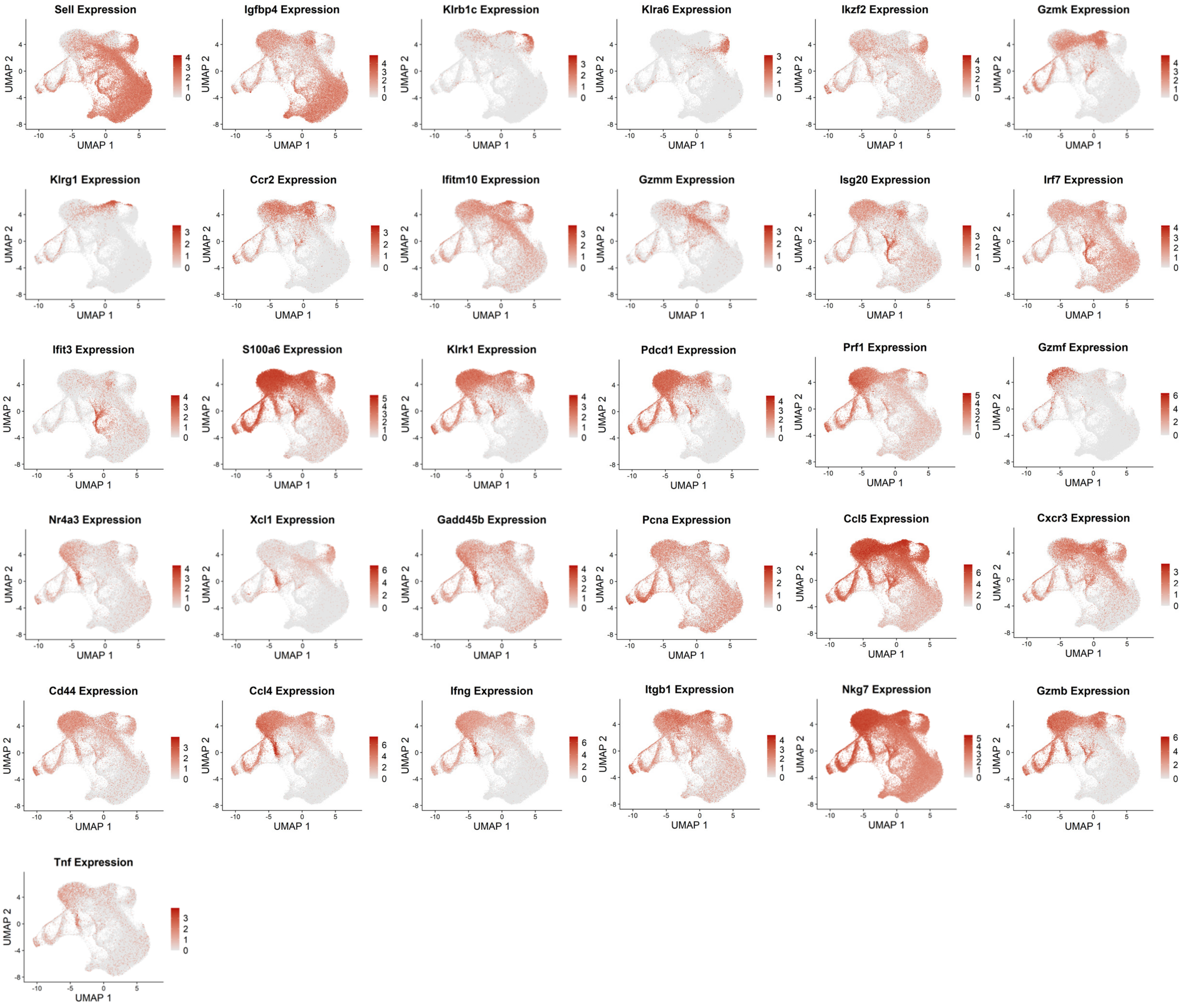

Supplemental Figure 4A

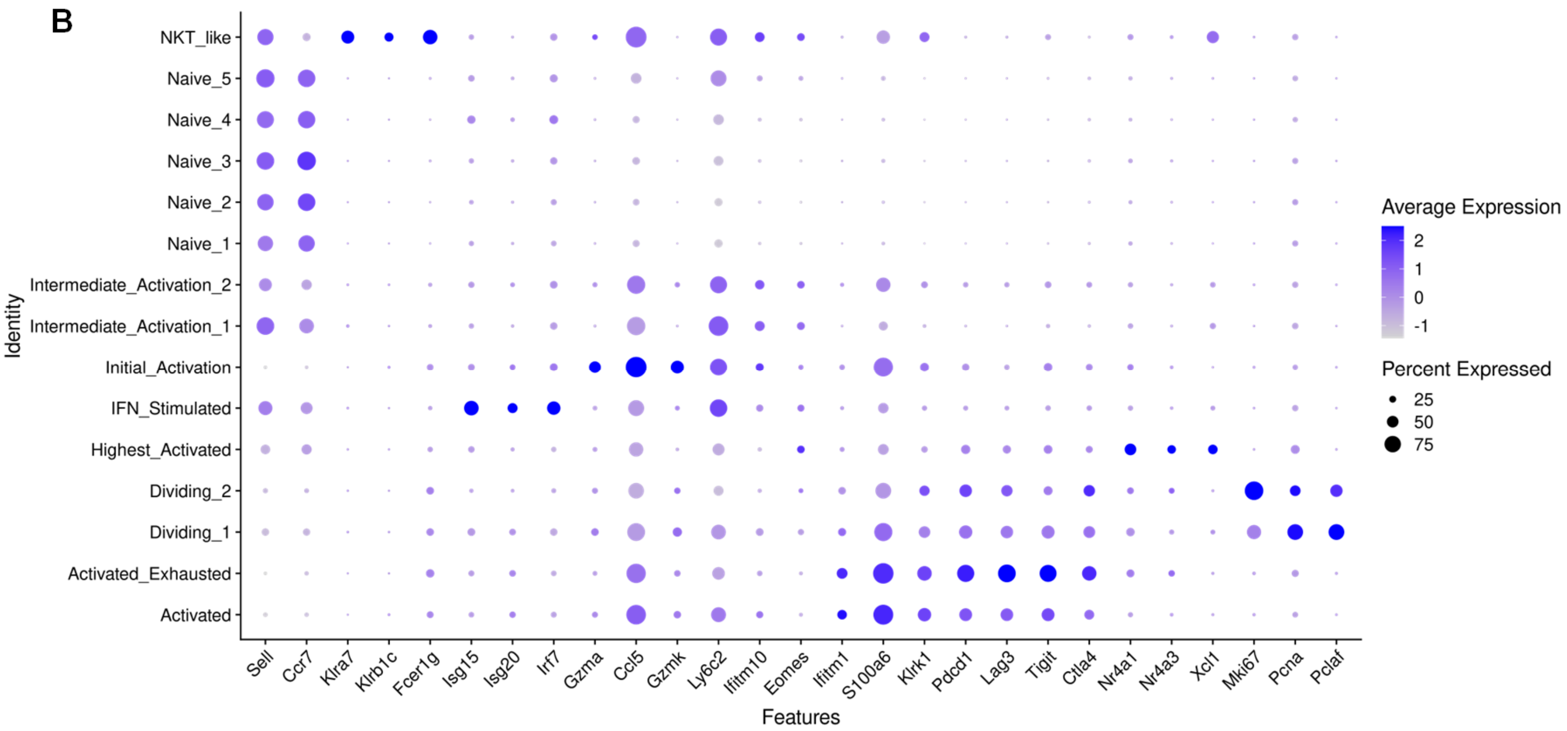

Supplemental Figure 4B

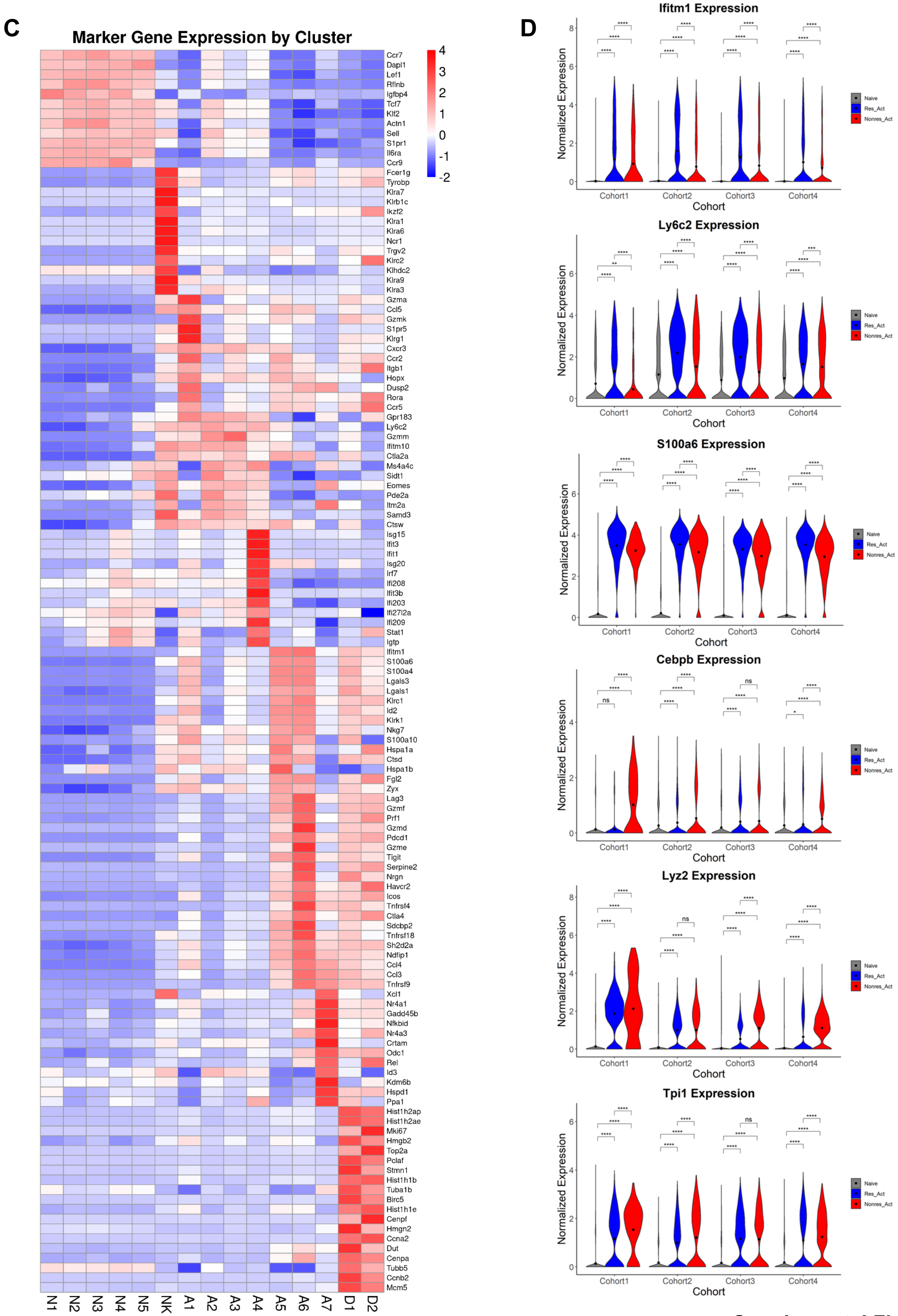

Supplemental Figure 4C,D

Supplemental Figure 4: Both responder (R) and non-responder (NR) CD8 TILs were activated. Gene expression data of 74260 cells from all 16 samples (RSP1-4, NRSP1-4, RTIL1-4, NRTIL1-4) were clustered using UMAP. **(A)** UMAPs of the normalized expression of 30 representative T cell genes in different clusters of the UMAP (gray=little to no expression; red=high expression). **(B)** Dot plot showing the gene expression of representative genes for each cluster of the UMAP. **(C)** Heatmap of the expression of representative genes for each cluster of the UMAP identified using FindMarkers function in Seurat. The average expression of all cells in each cluster was plotted. Color designation is scaled by row. **(D)** Violin plots showing the gene expression in Figure 5G analyzed by separating cells into four respective sequencing cohorts (Cohort 1: R#1 and NR#1; Cohort 2: R#2 and NR#2; Cohort 3: R#3 and NR#3, Cohort 4: R#4 and NR#4). Representative genes upregulated in R or NR activated TILs (residing in one of the seven activated clusters: A1-A7) compared to naïve T cells (residing in N1-N5 clusters). Naïve; naïve clusters in all samples, Res_Act; activated clusters in R TIL samples, Nonres_Act; activated clusters in NR TIL samples. Black dot indicates the mean of each group. Differences were evaluated using two-way ANOVA for Activation group (Res_Act, Nonres_Act, Naïve) in each cohort followed by Tukey's multiple comparison test (*, $P<0.05$; **, $P<0.01$; ***, $P<0.001$; ****, $P<0.0001$).

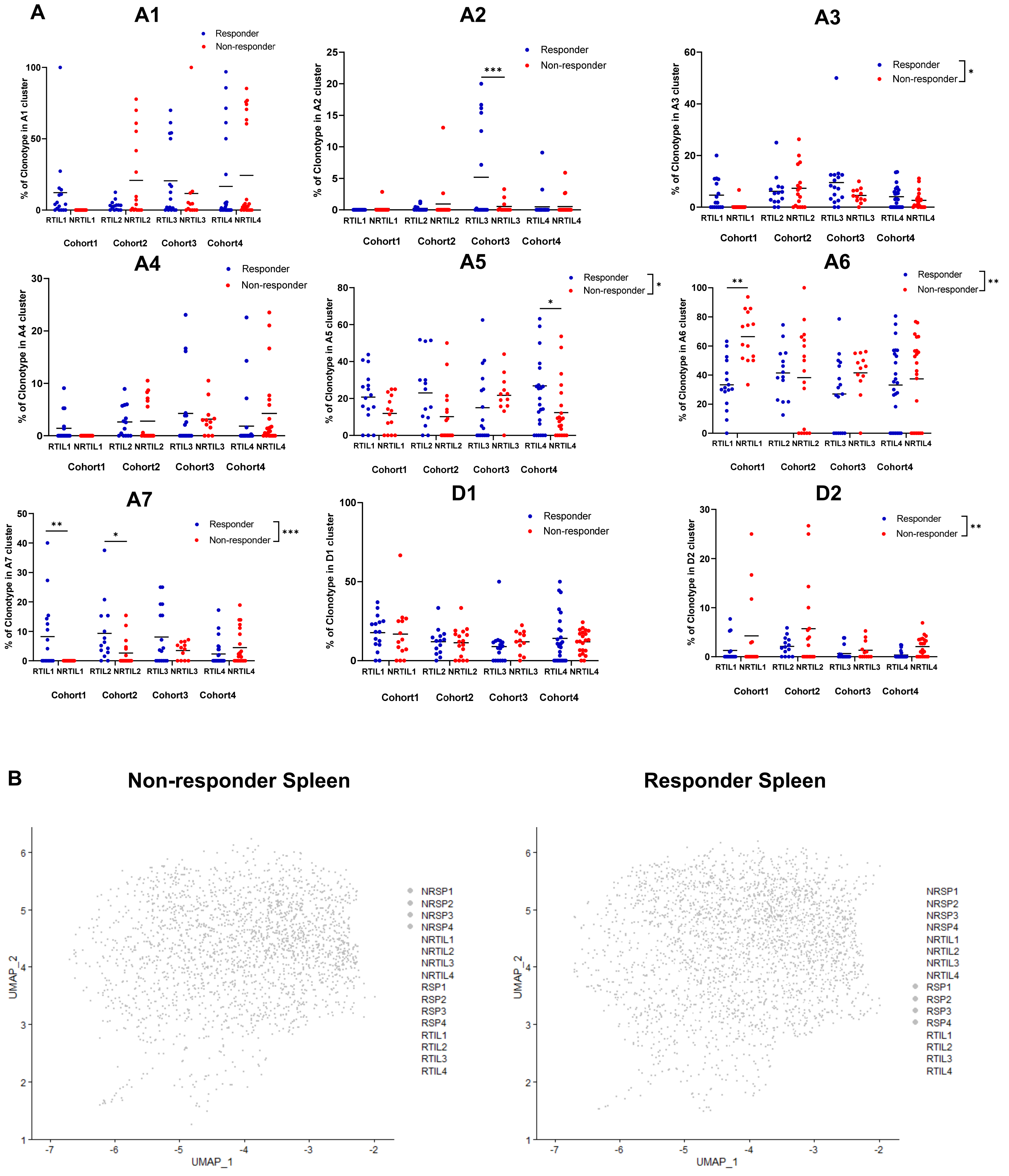

Supplemental Figure 5A,B

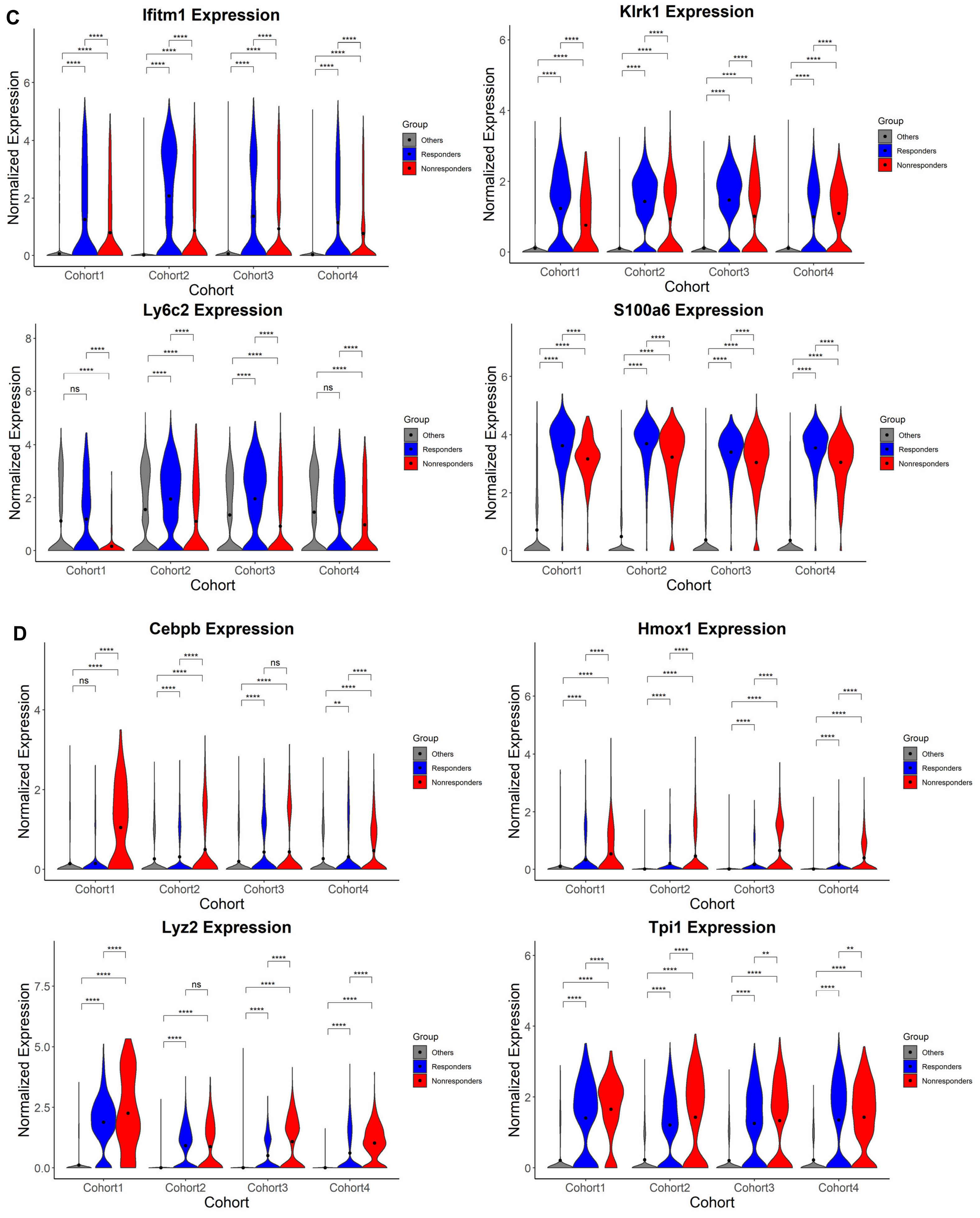

Supplemental Figure 5C, D

Supplemental Figure 5: Top TCR clonotypes of CD8 TILs differentially occupy activation clusters in responder (R) TILs vs. NR TILs. **(A)** Data from figure 6B were analyzed by separating clonotypes into four respective sequencing cohorts (Cohort 1: R#1 and NR#1; Cohort 2: R#2 and NR#2; Cohort 3: R#3 and NR#3, Cohort 4: R#4 and NR#4). Quantification of percent of R (blue) or NR (red) top TCR clonotypes residing in each of activated clusters (A1-A7) or dividing clusters (D1-D2) of the UMAP in each of the sequencing cohort. Each dot represents a clonotype and the black line indicates the mean. Differences were evaluated using two-way ANOVA for progression group (R vs. NR) and for cohort. Statistical significance for the progression group was indicated on the right. Sidak's multiple comparison test was used to compare the samples in each cohort (*, $P<0.05$; **, $P<0.01$; ***, $P<0.001$). **(B)** Cells residing in A5 and A6 clusters from all R spleen (RSP1-4) and all NR spleen (NRSP1-4) samples were extracted and plotted into UMAP (gray). **(C-D)** Violin plots of the expression of representative genes upregulated in R **(C)** or NR **(D)** top clonotypes compared to "Other" clonotypes. Data in Figure 7D and 7E were analyzed by separating cells into four respective sequencing cohorts (Cohort 1-4) as described above. Black dot indicates the mean of each group. Differences were evaluated using two-way ANOVA for different groups (Responders, Nonresponders, Others) in each cohort followed by Tukey's multiple comparisons test (**, $P<0.01$; ****, $P<0.0001$).
