# Supplementary material for: Divergent outcomes of anti-PD-L1 treatment coupled with host-intrinsic differences in TCR repertoire and distinct T cell activation states in responding versus non-responding tumors
Source: Front Immunol. 2022 Oct 18;13:992630. doi: 10.3389/fimmu.2022.992630 (PMC9624473; doi:10.3389/fimmu.2022.992630)
Supplement: Supplemental Table 1 — 16 samples sequenced by Single-cell TCR and Single-cell RNA sequencing. [file Table_1.pdf]

Supplemental Table 1. 16 samples sequenced by Single-cell TCR and Single-cell RNA sequencing					
**Sample**	**Abbrev.**	**Mouse**	**Tissue**	**Chemistry**	**# Cells**
Responder TIL #1	RTIL1	Responder #1	Tumor	VDJ	2810
Non-responder TIL #1	NRTIL1	Non-responder #1	Tumor	VDJ	615
Responder TIL #2	RTIL2	Responder #2	Tumor	VDJ	5175
Non-responder TIL #2	NRTIL2	Non-responder #2	Tumor	VDJ	1248
Responder TIL #3	RTIL3	Responder #3	Tumor	VDJ	1777
Non-responder TIL #3	NRTIL3	Non-responder #3	Tumor	VDJ	1885
Responder TIL #4	RTIL4	Responder #4	Tumor	VDJ	3056
Non-responder TIL #4	NRTIL4	Non-responder #4	Tumor	VDJ	3043
Responder Spleen #1	RSP1	Responder #1	Spleen	VDJ	6802
Non-responder Spleen #1	NRSP1	Non-responder #1	Spleen	VDJ	3004
Responder Spleen #2	RSP2	Responder #2	Spleen	VDJ	6740
Non-responder Spleen #2	NRSP2	Non-responder #2	Spleen	VDJ	6219
Responder Spleen #3	RSP3	Responder #3	Spleen	VDJ	5070
Non-responder Spleen #3	NRSP3	Non-responder #3	Spleen	VDJ	4928
Responder Spleen #4	RSP4	Responder #4	Spleen	VDJ	6303
Non-responder Spleen #4	NRSP4	Non-responder #4	Spleen	VDJ	4536
Responder TIL #1	RTIL1	Responder #1	Tumor	5’	1312
Non-responder TIL #1	NRTIL1	Non-responder #1	Tumor	5’	311
Responder TIL #2	RTIL2	Responder #2	Tumor	5’	7954
Non-responder TIL #2	NRTIL2	Non-responder #2	Tumor	5’	1386
Responder TIL #3	RTIL3	Responder #3	Tumor	5’	1765
Non-responder TIL #3	NRTIL3	Non-responder #3	Tumor	5’	1880
Responder TIL #4	RTIL4	Responder #4	Tumor	5’	3655
Non-responder TIL #4	NRTIL4	Non-responder #4	Tumor	5’	3202
Responder Spleen #1	RSP1	Responder #1	Spleen	5’	8119
Non-responder Spleen #1	NRSP1	Non-responder #1	Spleen	5’	3523
Responder Spleen #2	RSP2	Responder #2	Spleen	5’	8007
Non-responder Spleen #2	NRSP2	Non-responder #2	Spleen	5’	7191
Responder Spleen #3	RSP3	Responder #3	Spleen	5’	7117
Non-responder Spleen #3	NRSP3	Non-responder #3	Spleen	5’	5701
Responder Spleen #4	RSP4	Responder #4	Spleen	5’	8366
Non-responder Spleen #4	NRSP4	Non-responder #4	Spleen	5’	4771

Supplemental Table 1: 16 Samples sequenced by 10X Genomics Single-Cell Sequencing. Tumors and corresponding spleens from 4 responding mice and 4 non-responding mice were removed and tumors were digested. The first cohort of mouse tumors and spleens (Responder #1 and Non-responder #1) were sorted for live $CD8^+$ T cells and subjected to 5’ library prep for RNA expression and TCR VDJ sequencing. The second, third and fourth cohort of mouse tumors and spleens (Responder #2 and Non-responder #2, Responder #3 and Non-responder #3, Responder #4 and Non-responder #4) were subjected to EasySep™ Mouse CD8a Positive Selection Kit II for isolation of $CD8^+$ T cells and subjected to 5’ library prep for RNA expression and TCR VDJ sequencing. # Cells in the last column represents the number of cells used after filtering for CD8 T cells with appropriate RNA expression (see details in Methods).

Supplemental Table 2: Detailed clonotype information for top 10 TCR clones in each sample

Sample	TCRα CDR3	TCRαV	TCRαJ	TCRβ CDR3	TCRβV	TCRβD	TCRβJ	TCRα CDR3 #2	TCRαV #2	TCRαJ #2	% of Sample
RTIL1	CAMREGSSSFSKLVF	TRAV16	TRAJ50	CASSSSGQGAGDTQYF	TRBV29	TRBD1	TRBJ2-5	NA	NA	NA	13.13
	CALSPPMGYKLTF	TRAV6N-6	TRAJ9	CASSDRGPSAETLYF	TRBV13-3	None	TRBJ2-3	NA	NA	NA	8.68
	CAASYTEGADRLTF	TRAV5-4	TRAJ45	CGASRGSEQYF	TRBV20	None	TRBJ2-7	NA	NA	NA	8.65
	CALSEAMGYKLTF	TRAV6N-6	TRAJ9	CASSLSKDWGSSQNTLYF	TRBV16	None	TRBJ2-4	NA	NA	NA	4.77
	CAAAGANYNVLYF	TRAV4D-3	TRAJ21	CASSDTGGQDTQYF	TRBV13-3	None	TRBJ2-5	NA	NA	NA	3.99
	CALGAAANSGTYQRF	TRAV6-6	TRAJ13	CASSLGWGNQDTQYF	TRBV26	None	TRBJ2-5	NA	NA	NA	1.85
	CAASEGAGNTGKLIF	TRAV14N-3	TRAJ37	CASSAHWGGLAEQFF	TRBV10	None	TRBJ2-1	NA	NA	NA	1.60
	CALEGIASSSFSKLVF	TRAV17	TRAJ50	CASSSTGGTNERLFF	TRBV10	None	TRBJ1-4	NA	NA	NA	1.39
	CAAAGAGANTGKLTF	TRAV14-2	TRAJ52	CASSLGTGGYEQYF	TRBV12-1	None	TRBJ2-7	CALSDSGGSNAKLTF	TRAV12-2	TRAJ42	1.00
	CAASGANTNKVVF	TRAV14N-3	TRAJ34	CASSVTGGQDTQYF	TRBV13-3	None	TRBJ2-5	NA	NA	NA	0.96
RTIL2	CAMRANMGYKLTF	TRAV6-3	TRAJ9	CASSYEGNTGQLYF	TRBV10	None	TRBJ2-2	NA	NA	NA	45.28
	CAASLSNYNVLYF	TRAV14-1	TRAJ21	CASRQGNTEVFF	TRBV13-3	None	TRBJ1-1	NA	NA	NA	4.44
	CAASASNYAQGLTF	TRAV14N-3	TRAJ26	CASSLDITANTGQLYF	TRBV16	None	TRBJ2-2	NA	NA	NA	3.63
	CAASEHASSGSWQLIF	TRAV7-4	TRAJ22	CASSLEGTGGYEQYF	TRBV16	None	TRBJ2-7	NA	NA	NA	2.28
	CAVSMANYAQGLTF	TRAV7-5	TRAJ26	CASSPDNSQNTLYF	TRBV29	None	TRBJ2-4	NA	NA	NA	2.20
	CAGTGANTGKLTF	TRAV14D-3-DV8	TRAJ52	CASSIDWGGANTGQLYF	TRBV19	None	TRBJ2-2	NA	NA	NA	1.76
	CAASEHASSGSWQLIF	TRAV7D-4	TRAJ22	CASSLEGTGGYEQYF	TRBV16	None	TRBJ2-7	NA	NA	NA	1.43
	CAASEHASSGSWQLIF	TRAV7D-4	TRAJ22	CASSTEGTGGYEQYF	TRBV16	None	TRBJ2-7	NA	NA	NA	1.43
	CAASEHASSGSWQLIF	TRAV7D-4	TRAJ22	CAWSPRGPNSDYTF	TRBV31	None	TRBJ1-2	NA	NA	NA	1.29
	CAASEHASSGSWQLIF	TRAV7-4	TRAJ22	CASSLEGTGGYEQYF	TRBV16	None	TRBJ2-7	NA	NA	NA	1.28
RTIL3	CAASPGASSGSWQLIF	TRAV14D-3-DV8	TRAJ22	CASSLEPTGGYEQYF	TRBV16	None	TRBJ2-7	NA	NA	NA	26.28
	CALGSNMGYKLTF	TRAV6-6	TRAJ9	CASSGQGNYAEQFF	TRBV13-3	None	TRBJ2-1	NA	NA	NA	25.72
	CALGSNMGYKLTF	TRAV6-6	TRAJ9	CASSGQGNYAEQFF	TRBV13-3	None	TRBJ2-1	NA	NA	NA	9.85
	CAASPHASSGSWQLIF	TRAV14-1	TRAJ22	CASSLEGTGGYEQYF	TRBV16	None	TRBJ2-7	NA	NA	NA	3.43
	CAMERANTNKVVF	TRAV13-4-DV7	TRAJ34	CASSETGGQDTQYF	TRBV13-3	None	TRBJ2-5	NA	NA	NA	2.76
	CAAGSGYNKLTF	TRAV4D-3	TRAJ11	CASSGLAETLYF	TRBV14	None	TRBJ2-3	NA	NA	NA	1.86
	CVLGGANTNKVVF	TRAV6-2	TRAJ34	CTCSADQQGGTGQLYF	TRBV1	None	TRBJ2-2	NA	NA	NA	1.58
	CAVSMSNYNVLYF	TRAV7N-6	TRAJ21	CASSAQQGGQDTQYF	TRBV13-1	None	TRBJ2-5	NA	NA	NA	1.52
	CVLSPSIASSSFSKLVF	TRAV9-2	TRAJ50	CASSSGGAEQFF	TRBV14	None	TRBJ2-1	NA	NA	NA	1.52
	CSAMMDSNYQLIW	TRAV5-1	TRAJ33	CASSQEGDRYEQYF	TRBV2	None	TRBJ2-7	NA	NA	NA	1.35
RTIL4	CAASSRNYAQGLTF	TRAV10	TRAJ26	CASSLDRGDSDYTF	TRBV29	None	TRBJ1-2	NA	NA	NA	14.10
	CAVSATNYNVLYF	TRAV3D-3	TRAJ21	CASRRDNQDTQYF	TRBV29	None	TRBJ2-5	NA	NA	NA	10.08
	CAASGASNNRIFF	TRAV14D-1	TRAJ31	CASSSEWGGQDTQYF	TRBV10	None	TRBJ2-5	NA	NA	NA	9.10
	CAASLSSGSWQLIF	TRAV14D-2	TRAJ22	CASSFLTNTEVFF	TRBV14	None	TRBJ1-1	NA	NA	NA	6.05
	CAASAQGGRALIF	TRAV14D-2	TRAJ15	CASSDWGSSYEQYF	TRBV16	None	TRBJ2-7	NA	NA	NA	5.27
	CATGSSNTNKVVF	TRAV8N-2	TRAJ34	CSSSPDRGQNTLYF	TRBV23	None	TRBJ2-4	NA	NA	NA	4.97
	CAASRTNYNVLYF	TRAV14D-1	TRAJ21	CTCSADQSNERLFF	TRBV1	None	TRBJ1-4	NA	NA	NA	4.25
	CAASEHASSGSWQLIF	TRAV7-4	TRAJ22	CASSLEGTGGYEQYF	TRBV16	None	TRBJ2-7	NA	NA	NA	1.60
	CAADGANTGKLTF	TRAV14-3	TRAJ52	CTCSADRNYEQYF	TRBV1	None	TRBJ2-7	NA	NA	NA	1.51
	CALGDQGNTGKLTF	TRAV6-6	TRAJ27	CASSLELGGLEQYF	TRBV12-1	None	TRBJ2-7	NA	NA	NA	1.41
NRTIL1	CAASSRSNNRIFF	TRAV7D-2	TRAJ31	CATGTGGSDYTF	TRBV13-3	TRBD1	TRBJ1-2	NA	NA	NA	13.66
	CAMERANTNKVVF	TRAV13-4-DV7	TRAJ34	CASSDTGGQDTQYF	TRBV13-3	None	TRBJ2-5	NA	NA	NA	8.78
	CAASGANYNVLYF	TRAV10	TRAJ21	CASSVTGGSYEQYF	TRBV13-3	None	TRBJ2-7	NA	NA	NA	6.18
	CALTRNNYAQGLTF	TRAV6N-6	TRAJ26	CTCSADQQQAPLF	TRBV1	None	TRBJ1-5	NA	NA	NA	6.02
	CAMERANTNKVVF	TRAV13-4-DV7	TRAJ34	CASSGTGGQDTQYF	TRBV13-3	None	TRBJ2-5	NA	NA	NA	5.53
	CATSNMGYKLTF	TRAV14D-2	TRAJ9	CTCSADLAANTGQLYF	TRBV1	None	TRBJ2-2	NA	NA	NA	4.72
	CALQYAGGLSGKLTF	TRAV6-7-DV9	TRAJ2	CASSLEGTGGYEQYF	TRBV16	TRBD1	TRBJ2-7	CALSEGASSGSWQLIF	TRAV6N-6	TRAJ22	4.23
	CAMERANTNKVVF	TRAV13-4-DV7	TRAJ34	CASSGTGGQDTQYF	TRBV13-3	None	TRBJ2-5	CALWELMATGGNNKLTF	TRAV15-1-DV6-1	TRAJ56	3.74
	CAVSATGNYKYVF	TRAV9D-1	TRAJ40	CTCSADGFNYAEQFF	TRBV1	None	TRBJ2-1	NA	NA	NA	3.58
	CALSEGASSGSWQLIF	TRAV6N-6	TRAJ22	CASSLEGTGGYEQYF	TRBV16	TRBD1	TRBJ2-7	CALQYAGGLSGKLTF	TRAV6-7-DV9	TRAJ2	2.28
NRTIL2	CAASMANYNVLYF	TRAV14D-1	TRAJ21	CASSDAQQAPLF	TRBV13-3	None	TRBJ1-5	NA	NA	NA	17.63
	CAVEAPNYNVLYF	TRAV4-2	TRAJ21	CTCSADQQSEVFF	TRBV1	None	TRBJ1-1	NA	NA	NA	7.85
	CAVVSGGSNYKLTF	TRAV3-4	TRAJ53	CASSLEAGGGTEVFF	TRBV16	None	TRBJ1-1	NA	NA	NA	7.61
	CAVGGANYNVLYF	TRAV9D-3	TRAJ21	CASSPGQLSGNTLYF	TRBV29	None	TRBJ1-3	NA	NA	NA	4.97
	CATGGSNNRLTL	TRAV8D-2	TRAJ7	CTCSAGRENYAEQFF	TRBV1	None	TRBJ2-1	NA	NA	NA	4.57
	CALTLDYSNNRLTL	TRAV9N-4	TRAJ7	CASSLSQNTLYF	TRBV16	None	TRBJ2-4	NA	NA	NA	3.77
	CAVSGSNYNVLYF	TRAV9D-3	TRAJ21	CTCSADRGRGDQDTQYF	TRBV1	None	TRBJ2-5	NA	NA	NA	2.56
	CAASPSASSGSWQLIF	TRAV10	TRAJ22	CASSVEGSSYEQYF	TRBV16	None	TRBJ2-7	NA	NA	NA	1.84
	CAMREGGSSGSWQLIF	TRAV16D-DV11	TRAJ22	CASGESGGNERLFF	TRBV12-2+TRBV13-2	None	TRBJ1-4	NA	NA	NA	1.84
	CAMNDTNAYKVIF	TRAV13-4-DV7	TRAJ30	CASSLTKNTEVFF	TRBV10	None	TRBJ1-1	CALNNQGKLIF	TRAV17	TRAJ23	1.76
NRTIL3	CAASGANTNKVVF	TRAV14D-3-DV8	TRAJ34	CASSPDGSNSYNSPLYF	TRBV13-3	None	TRBJ1-6	NA	NA	NA	35.12
	CAVSMNNYAQGLTF	TRAV7D-5	TRAJ26	CASSDGGATGQLYF	TRBV13-1	None	TRBJ2-2	NA	NA	NA	7.32
	CAVSPPANYAQGLTF	TRAV3D-3	TRAJ26	CTCSADQGNQDTQYF	TRBV1	None	TRBJ2-5	NA	NA	NA	6.15
	CAAEAREYNQGKLIF	TRAV4D-4	TRAJ23	CASSRAGGYEQYF	TRBV13-3	None	TRBJ2-7	CAAGGANTNKVVF	TRAV14N-3	TRAJ34	3.87
	CALGSNMGYKLTF	TRAV6-6	TRAJ9	CASSPQGNYAEQFF	TRBV13-3	None	TRBJ2-1	NA	NA	NA	3.34
	CAAGGANTNKVVF	TRAV14N-3	TRAJ34	CASSRAGGYEQYF	TRBV13-3	None	TRBJ2-7	CAAEAREYNQGKLIF	TRAV4D-4	TRAJ23	3.18
	CAASNMGYKLTF	TRAV14D-2	TRAJ9	CTCSADLASNTGQLYF	TRBV1	None	TRBJ2-2	NA	NA	NA	2.65
	CALGDQGNTGKLIF	TRAV6-6	TRAJ37	CASSLELGGLEQYF	TRBV12-1	None	TRBJ2-7	NA	NA	NA	2.65
	CAASATNTGKLTF	TRAV14D-3-DV8	TRAJ27	CASSNDWGGADTLYF	TRBV19	None	TRBJ2-4	NA	NA	NA	2.49
	CAASATSSGQKLVF	TRAV14D-3-DV8	TRAJ16	CASSLPGTEVFF	TRBV19	None	TRBJ1-1	NA	NA	NA	2.12
NRTIL4	CAVSSSGSWQLIF	TRAV14D-2	TRAJ22	CASNKNSQNTLYF	TRBV14	None	TRBJ2-4	NA	NA	NA	7.72
	CALSRNNYAQGLTF	TRAV6N-6	TRAJ26	CASSDAGGAAEQFF	TRBV13-3	None	TRBJ2-1	NA	NA	NA	7.56
	CALSGMSNYNVLYF	TRAV12-2	TRAJ21	CASSDRGASAETLYF	TRBV13-3	None	TRBJ2-3	NA	NA	NA	5.59
	CAMDLNTEGADRLTF	TRAV13-4-DV7	TRAJ45	CASSDNRVYEQYF	TRBV13-1	None	TRBJ2-7	NA	NA	NA	5.16
	CAASGTNTGKLTF	TRAV14N-3	TRAJ27	CASSINWGGNTGQLYF	TRBV19	None	TRBJ2-2	NA	NA	NA	4.40
	CALGSNMGYKLTF	TRAV6-6	TRAJ9	CASSGQGNYAEQFF	TRBV13-3	None	TRBJ2-1	NA	NA	NA	3.06
	CAMERANTNKVVF	TRAV13-4-DV7	TRAJ34	CASSGTGGQDTQYF	TRBV13-3	None	TRBJ2-5	NA	NA	NA	2.76
	CAASESSGSWQLIF	TRAV14N-3	TRAJ22	CASSGGLYNSPLYF	TRBV14	None	TRBJ1-6	NA	NA	NA	2.53
	CAVRTDYSNNRLTL	TRAV9D-3	TRAJ7	CASSQDDTEVFF	TRBV2	None	TRBJ1-1	NA	NA	NA	2.53
	CAAGAGGNNKLTF	TRAV19	TRAJ56	CAITGGYEQYF	TRBV12-2+TRBV13-2	None	TRBJ2-7	NA	NA	NA	2.14

Supplemental Table 2: Detailed clonotype information for top 10 TCR clones in each sample (Continued)

Sample	TCRα CDR3	TCRαV	TCRαJ	TCRβ CDR3	TCRβV	TCRβD	TCRβJ	TCRα CDR3 #2	TCRαV #2	TCRαJ #2	% of Sample
RSP1	CAASGANTNKVVF	TRAV14N-3	TRAJ34	CASSVTGGQDTQYF	TRBV13-3	None	TRBJ2-5	NA	NA	NA	0.40
	CAASYTEGADRLTF	TRAV5-4	TRAJ45	CGASRGSEQYF	TRBV20	None	TRBJ2-7	NA	NA	NA	0.29
	CAVSDTSSGSWQLIF	TRAV3D-3	TRAJ22	CASSRTGDYAEQFF	TRBV13-3	None	TRBJ2-1	NA	NA	NA	0.29
	CALSPPMGYKLTF	TRAV6N-6	TRAJ9	CASSDRGPSAETLYF	TRBV13-3	None	TRBJ2-3	NA	NA	NA	0.24
	CAAAGANYNVLYF	TRAV4D-3	TRAJ21	CASSDTGGQDTQYF	TRBV13-3	None	TRBJ2-5	NA	NA	NA	0.22
	CALGAAANSGTYQRF	TRAV6-6	TRAJ13	CASSLGWGNQDTQYF	TRBV26	None	TRBJ2-5	NA	NA	NA	0.21
	CAMGSSSGSWQLIF	TRAV13N-4	TRAJ22	CASSLGGYEQYF	TRBV19	None	TRBJ2-7	NA	NA	NA	0.15
	CALEGIASSSFSKLVF	TRAV17	TRAJ50	CASSSTGGTNERLFF	TRBV10	None	TRBJ1-4	NA	NA	NA	0.13
	CALSEAMGYKLTF	TRAV6N-6	TRAJ9	CASSLSKDWGSSQNTLYF	TRBV16	None	TRBJ2-4	NA	NA	NA	0.13
	CAVRNSNNRIFF	TRAV7-1	TRAJ31	CASSFFPGGPYEQYF	TRBV14	None	TRBJ2-7	NA	NA	NA	0.13
RSP2	CAASASNYAQGLTF	TRAV14N-3	TRAJ26	CASSLDITANTGQLYF	TRBV16	None	TRBJ2-2	NA	NA	NA	0.62
	CAVSARGAQGLTF	TRAV9-1	TRAJ26	CASSPSTGFGNTLYF	TRBV5	None	TRBJ1-3	NA	NA	NA	0.58
	CAVLMDYANKMIF	TRAV7-5	TRAJ47	CASSPGLGENTLYF	TRBV12-2	None	TRBJ2-4	NA	NA	NA	0.40
	CAGTGANTGKLTF	TRAV14D-3-DV8	TRAJ52	CASSIDWGGANTGQLYF	TRBV19	None	TRBJ2-2	NA	NA	NA	0.27
	CAASLSNYNVLYF	TRAV14-1	TRAJ21	CASRQGNTEVFF	TRBV13-3	None	TRBJ1-1	NA	NA	NA	0.24
	CAASENSAGNKLTF	TRAV7D-4	TRAJ17	CASSQDGHLISNERLFF	TRBV5	None	TRBJ1-4	NA	NA	NA	0.19
	CAMRGDTNAYKVIF	TRAV6-3	TRAJ30	CASRGGANERLFF	TRBV13-1	None	TRBJ1-4	NA	NA	NA	0.13
	CALSPATSSGQKLVF	TRAV6N-6	TRAJ16	CASSQGYAEQFF	TRBV13-3	None	TRBJ2-1	NA	NA	NA	0.10
	CAAIMATGGNNKLTF	TRAV14-3	TRAJ56	CASKTGAYAEQFF	TRBV13-1	None	TRBJ2-1	CILRVNQGGSAKLIF	TRAV21-DV12	TRAJ57	0.09
	CAMRANMGYKLTF	TRAV6-3	TRAJ9	CASSYEGNTGQLYF	TRBV10	None	TRBJ2-2	NA	NA	NA	0.09
RSP3	CALGSNMGYKLTF	TRAV6-6	TRAJ9	CASSGQGNYAEQFF	TRBV13-3	None	TRBJ2-1	NA	NA	NA	1.70
	CAASPHASSGSWQLIF	TRAV14-1	TRAJ22	CASSLEGTGGYEQYF	TRBV16	None	TRBJ2-7	NA	NA	NA	0.75
	CAMERANTNKVVF	TRAV13-4-DV7	TRAJ34	CASSETGGQDTQYF	TRBV13-3	None	TRBJ2-5	NA	NA	NA	0.55
	CAASPGASSGSWQLIF	TRAV14D-3-DV8	TRAJ22	CASSLEPTGGYEQYF	TRBV16	None	TRBJ2-7	NA	NA	NA	0.53
	CAVSMSNYNVLYF	TRAV7N-6	TRAJ21	CASSAQQGGQDTQYF	TRBV13-1	None	TRBJ2-5	NA	NA	NA	0.53
	CAAGSGYNKLTF	TRAV4D-3	TRAJ11	CASSGLAETLYF	TRBV14	None	TRBJ2-3	NA	NA	NA	0.51
	CAASEGTGNYKYVF	TRAV14D-1	TRAJ40	CASSVDRAFSNERLFF	TRBV13-1	None	TRBJ1-4	NA	NA	NA	0.28
	CAMERANTNKVVF	TRAV13-4-DV7	TRAJ34	CASSLTGGQDTQYF	TRBV13-3	None	TRBJ2-5	NA	NA	NA	0.28
	CAIPGANTGKLTF	TRAV13-4-DV7	TRAJ52	CASTTGPEVFF	TRBV29	None	TRBJ1-1	NA	NA	NA	0.22
	CATDNSGTYQRF	TRAV8N-2	TRAJ13	CASSFGTTNSDYTF	TRBV3	None	TRBJ1-2	NA	NA	NA	0.18
RSP4	CAASRTNYNVLYF	TRAV14D-1	TRAJ21	CTCSADQSNERLFF	TRBV1	None	TRBJ1-4	NA	NA	NA	0.83
	CAAPSNTGNYKYVF	TRAV7-6	TRAJ40	CASSINTEVFF	TRBV19	None	TRBJ1-1	NA	NA	NA	0.75
	CAASAQGGRALIF	TRAV14D-2	TRAJ15	CASSDWGSSYEQYF	TRBV16	None	TRBJ2-7	NA	NA	NA	0.56
	CAASGASNNRIFF	TRAV14D-1	TRAJ31	CASSSEWGGQDTQYF	TRBV10	None	TRBJ2-5	NA	NA	NA	0.43
	CAVSATNYNVLYF	TRAV3D-3	TRAJ21	CASRRDNQDTQYF	TRBV29	None	TRBJ2-5	NA	NA	NA	0.35
	CAASSNQGGSAKLIF	TRAV19	TRAJ57	CASSDPGGNTGQLYF	TRBV13-3	None	TRBJ2-2	NA	NA	NA	0.25
	CAVEHNNAGAKLTF	TRAV7-5	TRAJ39	CASSLSNYEQYF	TRBV29	None	TRBJ2-7	NA	NA	NA	0.25
	CAGNRGSALGRLHF	TRAV9D-3	TRAJ18	CASGDEQGTNERLFF	TRBV12-2+TRBV13-2	None	TRBJ1-4	NA	NA	NA	0.22
	CAVSEGKVVF	TRAV9-4	TRAJ34	CASGEWDNYAEQFF	TRBV12-2+TRBV13-2	None	TRBJ2-1	NA	NA	NA	0.21
	CVPGGRSNAKLTF	TRAV7-2	TRAJ42	CTCSADRGWEQYF	TRBV1	None	TRBJ2-7	NA	NA	NA	0.21
NRSP1	CAVSATGNYKYVF	TRAV9D-1	TRAJ40	CTCSADGFNYAEQFF	TRBV1	None	TRBJ2-1	NA	NA	NA	0.53
	CALTRNNYAQGLTF	TRAV6N-6	TRAJ26	CTCSADQQQAPLF	TRBV1	None	TRBJ1-5	NA	NA	NA	0.30
	CATDDQGGRALIF	TRAV8D-2	TRAJ15	CASSLLGQGAEQFF	TRBV26	TRBD1	TRBJ2-1	NA	NA	NA	0.27
	CAVSASGYQNFYF	TRAV9-4	TRAJ49	CASGDRDRLYEQYF	TRBV12-2+TRBV13-2	None	TRBJ2-7	NA	NA	NA	0.27
	CAASSRSNNRIFF	TRAV7D-2	TRAJ31	CATGTGGSDYTF	TRBV13-3	TRBD1	TRBJ1-2	NA	NA	NA	0.23
	CAMERANTNKVVF	TRAV13-4-DV7	TRAJ34	CASSDTGGQDTQYF	TRBV13-3	None	TRBJ2-5	NA	NA	NA	0.20
	CAARARSNYNVLYF	TRAV14D-2	TRAJ21	CASSALGGYEQYF	TRBV2	None	TRBJ2-7	NA	NA	NA	0.17
	CAMERANTNKVVF	TRAV13-4-DV7	TRAJ34	CASSGTGGQDTQYF	TRBV13-3	None	TRBJ2-5	NA	NA	NA	0.17
	CAASEQGGRALIF	TRAV7D-2	TRAJ15	CASSDLQGEEQYF	TRBV13-3	None	TRBJ2-7	NA	NA	NA	0.13
	CAASGANYNVLYF	TRAV10	TRAJ21	CASSVTGGSYEQYF	TRBV13-3	None	TRBJ2-7	NA	NA	NA	0.13
NRSP2	CATGGSNNRLTL	TRAV8D-2	TRAJ7	CTCSAGRENYAEQFF	TRBV1	None	TRBJ2-1	NA	NA	NA	5.80
	CAVKRTASLGKLQF	TRAV7N-6	TRAJ24	CASSQVGTGNTLYF	TRBV5	None	TRBJ1-3	NA	NA	NA	3.47
	CAMRDRNQGGSAKLIF	TRAV16	TRAJ57	CASRGLGAALYF	TRBV29	None	TRBJ2-4	NA	NA	NA	0.39
	CVLSASSGSWQLIF	TRAV9-2	TRAJ22	CASSLQQDTQYF	TRBV29	None	TRBJ2-5	NA	NA	NA	0.21
	CAASGGGANTGKLTF	TRAV14-3	TRAJ52	CASSRGTGGYEQYF	TRBV12-1	None	TRBJ2-7	NA	NA	NA	0.19
	CATGNNRIFF	TRAV8-1	TRAJ31	CTCSAGKGYSDYTF	TRBV1	None	TRBJ1-2	NA	NA	NA	0.18
	CAVSGSNYNVLYF	TRAV9D-3	TRAJ21	CTCSADRGRGDQDTQYF	TRBV1	None	TRBJ2-5	NA	NA	NA	0.18
	CAVVSGGSNYKLTF	TRAV3-4	TRAJ53	CASSLEAGGGTEVFF	TRBV16	None	TRBJ1-1	NA	NA	NA	0.16
	CAMNDTNAYKVIF	TRAV13-4-DV7	TRAJ30	CASSLTKNTEVFF	TRBV10	None	TRBJ1-1	CALNNQGKLIF	TRAV17	TRAJ23	0.14
	CAMREGGSSGSWQLIF	TRAV16D-DV11	TRAJ22	CASGESGGNERLFF	TRBV12-2+TRBV13-2	None	TRBJ1-4	NA	NA	NA	0.13
NRSP3	CAASGANTNKVVF	TRAV14D-3-DV8	TRAJ34	CASSPDGSNSYNSPLYF	TRBV13-3	None	TRBJ1-6	NA	NA	NA	3.81
	CAAGGANTNKVVF	TRAV14N-3	TRAJ34	CASSRAGGYEQYF	TRBV13-3	None	TRBJ2-7	CAAEAREYNQGKLIF	TRAV4D-4	TRAJ23	0.32
	CSAKNYAQGLTF	TRAV5-1	TRAJ26	CASSFGANTEVFF	TRBV29	None	TRBJ1-1	NA	NA	NA	0.26
	CAAEAREYNQGKLIF	TRAV4D-4	TRAJ23	CASSRAGGYEQYF	TRBV13-3	None	TRBJ2-7	CAAGGANTNKVVF	TRAV14N-3	TRAJ34	0.22
	CAAGLNQGGSAKLIF	TRAV19	TRAJ57	CASSDPGGATGQLYF	TRBV13-3	None	TRBJ2-2	NA	NA	NA	0.16
	CAASATNTGKLTF	TRAV14D-3-DV8	TRAJ27	CASSNDWGGADTLYF	TRBV19	None	TRBJ2-4	NA	NA	NA	0.16
	CAASNMGYKLTF	TRAV14D-2	TRAJ9	CTCSADLASNTGQLYF	TRBV1	None	TRBJ2-2	NA	NA	NA	0.16
	CATDSQGGRALIF	TRAV8D-2	TRAJ15	CASSLSGQGGEQYF	TRBV26	None	TRBJ2-7	NA	NA	NA	0.16
	CAASETSSFSKLVF	TRAV5-4	TRAJ50	CASSSPDERLFF	TRBV12-1	None	TRBJ1-4	NA	NA	NA	0.14
	CALDYANKMIF	TRAV12-2	TRAJ47	CASSLGGPDYTF	TRBV12-1	None	TRBJ1-2	NA	NA	NA	0.14
NRSP4	CALSRNNYAQGLTF	TRAV6N-6	TRAJ26	CASSDAGGAAEQFF	TRBV13-3	None	TRBJ2-1	NA	NA	NA	0.68
	CAASGTNTGKLTF	TRAV14N-3	TRAJ27	CASSINWGGNTGQLYF	TRBV19	None	TRBJ2-2	NA	NA	NA	0.57
	CAAGLNQGGSAKLIF	TRAV19	TRAJ57	CASSDAGANTGQLYF	TRBV13-3	None	TRBJ2-2	NA	NA	NA	0.44
	CAVSSSGSWQLIF	TRAV14D-2	TRAJ22	CASNKNSQNTLYF	TRBV14	None	TRBJ2-4	NA	NA	NA	0.37
	CAAIMSNYNVLYF	TRAV14D-1	TRAJ21	CASGERQNTLYF	TRBV12-2+TRBV13-2	None	TRBJ2-4	NA	NA	NA	0.35
	CAGNRGSALGRLHF	TRAV9D-3	TRAJ18	CASGDDLGENTLYF	TRBV12-2+TRBV13-2	None	TRBJ2-4	NA	NA	NA	0.31
	CALSPPMGYKLTF	TRAV6N-6	TRAJ9	CASSLDRKTGSSQNTLYF	TRBV16	None	TRBJ2-4	NA	NA	NA	0.31
	CAAGANQGGSAKLIF	TRAV19	TRAJ57	CASSDPGGVTGQLYF	TRBV13-3	None	TRBJ2-2	NA	NA	NA	0.26
	CALSGMSNYNVLYF	TRAV12-2	TRAJ21	CASSDRGASAETLYF	TRBV13-3	None	TRBJ2-3	NA	NA	NA	0.24
	CAAGANQGGSAKLIF	TRAV19	TRAJ57	CASSDAGSNTGQLYF	TRBV13-3	None	TRBJ2-2	NA	NA	NA	0.22

Supplemental Table 2. Detailed clonotype information for top 10 TCR clones in each sample. CD8 T cells from each sample were grouped into clones by identical nucleotide sequences of the TCRα CDR3 and TCRβ CDR3 chains. The top 10 TCR clones by abundance in each sample are shown, with their corresponding CDR3 amino acid sequences, V, D, J gene usage, and percent (% = the number of cells in each clone / the number of total cells sequenced for a given sample). Occasionally, some clones contain two TCRα chains, which is plausible since allelic exclusion does not operate efficiently for the TCRα chain.

Supplemental Table 3: TCR clonotypes shared between different TIL samples

Clonotype (TCRa_TCRb CDR3)	Shared Clonotype #	RTIL1	RTIL2	RTIL3	RTIL4	NRTIL1	NRTIL2	NRTIL3	NRTIL4	Present in	#Samples
CAASEHASSGSWQLIF_CASSLEGTGGYEQYF	Shared Clonotype 1	0	277	8	49	11	0	1	23	RTIL2_RTIL3_RTIL4_NRTIL1_NRTIL3_NRTIL4	6
CALGSNMGYKLTF_CASSGQGNYAEQFF	Shared Clonotype 2	13	0	634	35	0	18	0	93	RTIL1_RTIL3_RTIL4_NRTIL2_NRTIL4	5
CAASGSGANTGKLTF_CASSPGTGGYEQYF		0	5	5	1	0	27	0	0	RTIL2_RTIL3_RTIL4_NRTIL2	4
CATDNSGTYQRF_CASSLGTTNSDYTF		3	0	1	0	0	0	1	2	RTIL1_RTIL3_NRTIL3_NRTIL4	4
CALSPPMGYKLTF_CASSDRGPSAETLYF		244	0	1	2	0	0	0	0	RTIL1_RTIL3_RTIL4	3
CAASPHASSGSWQLIF_CASSLEGTGGYEQYF		0	4	61	4	0	0	0	0	RTIL2_RTIL3_RTIL4	3
CAASATSSGQKLVF_CASSLPGTEVFF	Shared Clonotype 3	0	0	0	21	0	2	40	0	RTIL4_NRTIL2_NRTIL3	3
CAAGANQGGSAKLIF_CASSDAGANTGQLYF		0	0	7	8	0	0	0	4	RTIL3_RTIL4_NRTIL4	3
CATDYQGGRALIF_CASGGGTGRSNSDYTF		0	0	5	3	0	0	1	0	RTIL3_RTIL4_NRTIL3	3
CAASASNYNVLYF_CTCSADQTGSGNTLYF		0	0	2	1	1	0	0	0	RTIL3_RTIL4_NRTIL1	3
CAASPGASSGSWQLIF_CASSLEPTGGYEQYF		0	0	467	32	0	0	0	0	RTIL3_RTIL4	2
CAASSRNYAQGLTF_CASSLDRGDSDYTF		0	0	1	431	0	0	0	0	RTIL3_RTIL4	2
CAVSATNYNVLYF_CASRRDNQDTQYF		0	0	1	308	0	0	0	0	RTIL3_RTIL4	2
CAASAQGGRALIF_CASSDWGSSYEQYF		0	0	1	161	0	0	0	0	RTIL3_RTIL4	2
CAMERANTNKVVF_CASSGTGGQDTQYF		0	0	0	0	34	0	0	84	NRTIL1_NRTIL4	2
CALGSNMGYKLTF_CASSPQGNYAEQFF		0	0	0	1	0	0	63	0	RTIL4_NRTIL3	2
CAMERANTNKVVF_CASSDTGGQDTQYF		0	0	0	2	54	0	0	0	RTIL4_NRTIL1	2
CAMERANTNKVVF_CASSETGGQDTQYF		0	0	49	1	0	0	0	0	RTIL3_RTIL4	2
CAAEGNYQLIW_CASSPDWASAETLYF		0	0	10	0	0	0	0	34	RTIL3_NRTIL4	2
CAVSPSSGSWQLIF_CASSPQYAEQFF		0	0	2	39	0	0	0	0	RTIL3_RTIL4	2
CAAGLNQGGSAKLIF_CASSDAGANTGQLYF		0	0	0	0	0	0	5	36	NRTIL3_NRTIL4	2
CAVSEGKVVF_CASGEWDNYAEQFF		0	0	1	37	0	0	0	0	RTIL3_RTIL4	2
CAAGSGYNKLTF_CASSGLAETLYF		0	0	33	2	0	0	0	0	RTIL3_RTIL4	2
CVLGGANTNKVVF_CTCSADQQGGTGQLYF		0	0	28	2	0	0	0	0	RTIL3_RTIL4	2
CAVSMSNMGYKLTF_CASSLGLGGAETLYF		0	3	0	0	0	0	0	20	RTIL2_NRTIL4	2
CAAGENQGGSAKLIF_CASSDPGANTGQLYF		0	0	1	22	0	0	0	0	RTIL3_RTIL4	2
CATDAQGGRALIF_CASSLSGQGIEQYF		0	0	2	19	0	0	0	0	RTIL3_RTIL4	2
CATDPQGGRALIF_CASSLEWGPGQLYF		0	0	12	2	0	0	0	0	RTIL3_RTIL4	2
CAVEHNNAGAKLTF_CASSLSNYEQYF		0	0	0	13	0	0	1	0	RTIL4_NRTIL3	2
CAASNMGYKLTF_CASSPDYNYAEQFF		0	0	0	5	0	9	0	0	RTIL4_NRTIL2	2
CALSDRGGSNYKLTF_CASSLWVSYEQYF		0	0	3	0	0	10	0	0	RTIL3_NRTIL2	2
CAASEGTGNYKYVF_CASSVDRAFSNERLFF		0	0	10	1	0	0	0	0	RTIL3_RTIL4	2
CAASATSSGQKLVF_CASSLAHANSDYTF		0	0	0	8	0	0	0	1	RTIL4_NRTIL4	2
CAASATSGSWQLIF_CASGESQNTLYF		1	0	0	7	0	0	0	0	RTIL1_RTIL4	2
CATDPQGGRALIF_CASSLLGQGTEVFF		0	0	6	1	0	0	0	0	RTIL3_RTIL4	2
CAARGANSAGNKLTF_CTCSADRGWGNQDTQYF		0	0	5	0	0	0	1	0	RTIL3_NRTIL3	2
CALSDGGNMGYKLTF_CASSPGTGGYAEQFF		0	0	4	1	0	0	0	0	RTIL3_RTIL4	2
CAMRERTEGADRLTF_CASSFTDTEVFF		0	0	4	1	0	0	0	0	RTIL3_RTIL4	2
CAASADNYAQGLTF_CASSDAGTGYEQYF		0	2	0	1	0	0	0	0	RTIL2_RTIL4	2
CAASGGGANTGKLTF_CASSLGTGGYEQYF		0	2	1	0	0	0	0	0	RTIL2_RTIL3	2
CAASAANYNVLYF_CAVSSGSWQLIF_CASSDPRGAEQFF		0	0	1	2	0	0	0	0	RTIL3_RTIL4	2
CAASDYSNNRLTL_CASSQVQGSAETLYF		0	0	1	2	0	0	0	0	RTIL3_RTIL4	2
CALGDNSAGNKLTF_CASSDGTGYSDYTF		0	0	1	2	0	0	0	0	RTIL3_RTIL4	2
CAASEHASSGSWQLIF_CAMREGNNRIFF_CASSLEGTGGYEQYF		0	0	1	1	0	0	0	0	RTIL3_RTIL4	2
CAASGTGANTGKLTF_CASSSGTGGYEQYF		0	0	1	0	0	0	0	1	RTIL3_NRTIL4	2
CAASSMGYKLTF_CASSLEGSSYEQYF		0	0	1	0	0	0	1	0	RTIL3_NRTIL3	2
CALGDRDSSGQKLVF_CASSLDWGGGDTQYF		0	0	1	1	0	0	0	0	RTIL3_RTIL4	2
CALSSNMGYKLTF_CASGERDGNYAEQFF		0	0	1	1	0	0	0	0	RTIL3_RTIL4	2
CALSYNRIFF_CASARGSDYTF		0	0	1	1	0	0	0	0	RTIL3_RTIL4	2
CATVNNYAQGLTF_CASSRDRGNEQYF		0	0	1	1	0	0	0	0	RTIL3_RTIL4	2
CAASLTGNTGKLIF_CASSINSQNTLYF		0	0	0	0	1	1	0	0	NRTIL1_NRTIL2	2
CAAGLNQGGSAKLIF_CASSDAGGNTGQLYF		0	0	0	0	0	0	1	1	NRTIL3_NRTIL4	2
CALNPNTNKVVF_CASSDDEQYF		0	0	0	0	0	0	1	1	NRTIL3_NRTIL4	2

Supplemental Table 3: TCR clonotypes shared between different TIL samples. Eight TIL samples (RTIL1, RTIL2, RTIL3, RTIL4, NRTIL1, NRTIL2, NRTIL3, NRTIL4) were evaluated for any TCR clonotypes that are shared between samples. The 53 shared clonotypes are shown. For each TCR clonotype, values are shown for the number of cells in each of the 8 TIL samples. 3 of the shared clonotypes were chosen based on the sharing between responder and non-responder TIL samples and on whether there were at least 20 cells of that clonotype in more than one sample.

Supplemental Table 4: TCRβ CDR3 sequences of top 10 groups by GLIPH clustering

Group	Consensus	Sequence	RTIL1	RTIL2	RTIL3	RTIL4	NRTIL1	NRTIL2	NRTIL3	NRTIL4
1	CRG-CASSLERTGGYEQYF	CASSIEGTGGYEQYF	0.036	0	0	0	0	0	0	0
		CASSLEPTGGYEQYF	0	0	26.280	1.047	0	0	0	0
		CASSTEGTGGYEQYF	0	1.913	0	0	0	0	0	0
		CASSLERTGGYEQYF	0	0.058	0	0	0	0	0	0
		CASSLEGTGGYEQYF	0	6.357	4.052	2.062	9.756	0.080	0.053	2.005
		CASSLEGTGAYEQYF	0	0	0	0	0	0	0	0.361
		CASSLEGTGSYEQYF	0	0	0	0	0	0	0	0.329
2	CRG-CASSPQGNYAEQFF	CASSGQGNYAEQFF	0.498	0	35.678	1.211	0	1.442	0	3.056
		CASSPQGAYAEQFF	0	0	0	0	0	0	0	1.347
		CASSPQGNYAEQFF	0	0	0	0.033	0	0	3.342	0
3	CRG-CASSYEGNTGQLYF	CASSYEGNTGQLYF	0	45.894	0	0	0	0	0	0
4	CRG-CASSPDGSNSCNSPLYF	CASSPDGSNSCNSPLYF	0	0	0	0	0	0	0.053	0
		CASSPDGSNSYNSPLYF	0	0	0	0	0	0	35.119	0
5	CRG-CASSTTGGQDTQYF	CASSSTGGQNTLYF	0	0	0	0.033	0	0	0	0
		CASSLTGGQNTLYF	0	0	0	0	0	0	0	0.033
		CASSLLGPQDTQYF	0	0.019	0	0	0	0	0	0
		CASSRLGGQDTQYF	0.036	0	0	0	0	0	0	0
		CASSLTGGQDTQYF	0	0.019	0.169	0	0	0	0	0
		CASSSTGGQDTQYF	0	0	0	0	0	0	0.796	0
		CASSKLGAQDTQYF	0	0	0	0	0	0.240	0	0
		CASSDTGGQDTQYF	3.986	0	0	0.065	8.780	0	0	0
		CASSETGGQDTQYF	0	0.232	2.983	0.033	0	0.240	0	0
		CASSLMGGQNTLYF	0	0	0	0	0	0	0	0.263
		CASSGTGGQDTQYF	0	0	0	0	9.593	0	0.053	2.760
		CASSLLGGQDTQYF	0	0.019	0	0	0	0	0	0
		CASSLTGGADTLYF	0	0	0	0	0	0.080	0	0
		CASSLLGAQDTQYF	0	0	0	0.196	0	0	0	0
		CASSVTGGQDTQYF	0.996	0	0	0	0	0	0	0
		CASAVTGGQDTQYF	0	0	0	0.065	0	0	0	0
		CAWSLRGGQNTLYF	0	0.019	0	0	0	0	0	0
		CASSLAGGQDTQYF	0	0	0	0.033	0	0	0	0
		CASSTTGGQDTQYF	0.107	0	0	0	0	0	0	0
		CASSYLGGQDTQYF	0	0.019	0	0	0	0	0	0
6	CRG-CASSDAQQAPLF	CASSDAQQAPLF	0	0	0	0	0	17.628	0	0
7	CRG-CTCSADQQGGTGQLYF	CTCSADQQSEVFF	0	0	0	0	0	7.853	0	0
		CTCSADQQQAPLF	0	0	0	0	6.179	0	0	0
		CTCSADQQGGTGQLYF	0	0	1.576	0.065	0	0	0	0
		CTCSADQQYEQYF	0	0	0	0.033	0	0	0	0
8	CRG-CASSDRGPSAETLYF	CASSDRGASAETLYF	0	0	0	0	0	0	0	5.587
		CASSDRGPSAETLYF	8.683	0	0.056	0.065	0	0.160	0	0
9	CRG-CASSLDRGDSDYTF	CASSLDRGDSDYTF	0	0	0.056	14.300	0	0	0	0
10	CRG-CASRTGGSDYTF	CASRTGGSDYTF	0.036	0	0	0	0	0	0	0
		CATGTGGSDYTF	0	0	0	0	13.659	0	0	0
		CASGDGGSDYTF	0	0.019	0	0	0	0	0	0

Supplemental Table 4: TCRβ CDR3 sequences of top 10 groups by GLIPH clustering. All TCRβ CDR3 sequences from 8 TIL samples (RTIL1, RTIL2, RTIL3, RTIL4, NRTIL1, NRTIL2, NRTIL3, NRTIL4) were analyzed using the GLIPH algorithm. Specificity groups were ordered based on the sum of the abundance of each sequence in the specificity group in each of the 8 samples, to capture the most expanded clonotypes in the dataset and their related sequences. The 10 specificity groups with the highest sum of sequence abundances are shown. CDR3 sequences within these top 10 GLIPH groups are shown with their percent in each TIL sample (% = the number of a unique CDR3 sequence / the number of total CDR3 sequences in any given sample).

Supplemental Table 5: Differential gene expression between responder TILs and naïve T cells or between non-responder TILs and naïve T cells

Gene	Responder fold change	Responder adjusted p-value	Non-responder fold change	Non-responder adjusted p-value
Ccl5	32.609	0	32.992	6.41E-237
S100a6	27.904	0	20.489	0
Ccl4	26.614	0	20.525	0
Gzmb	16.324	0	13.763	0
Lgals1	11.144	0	9.870	1.29E-193
Ifitm1	12.698	0	7.280	3.18E-304
S100a4	10.484	0	9.095	0
Ccl3	8.332	0	7.519	1.37E-228
Lag3	8.298	0	7.544	0
Anxa2	7.745	0	7.699	0
Pdcd1	7.887	0	7.388	0
Tnfrsf9	7.372	0	7.494	0
Klrc1	7.676	0	6.708	0
Lgals3	7.241	0	6.950	0
Ifng	6.834	0	5.739	0
Ly6a	6.334	1.97E-301	5.915	6.29E-67
Rgs16	6.360	0	5.513	0
Prf1	6.081	0	5.413	4.47E-169
Nkg7	5.920	0	5.511	1.06E-115
AW112010	6.264	0	5.106	2.97E-95
Cxcr6	5.767	0	5.398	0
Ctla2a	5.851	0	5.266	0
Tigit	5.385	0	5.600	0
Sh2d2a	4.885	0	5.410	2.39E-187
Spp1	5.541	0	4.671	0
Gzma	4.396	6.41E-137	5.529	1.12E-107
Id2	4.862	0	4.724	4.83E-171
Bhlhe40	5.164	0	4.393	0
Ifitm2	4.959	0	4.368	0
Lyz2	3.460	0	5.820	1.41E-288
Capg	4.803	0	4.203	4.56E-285
Klrk1	4.792	0	3.949	0
Il2rb	4.572	0	4.107	2.21E-169
Apoe	3.405	2.33E-303	5.154	0
Klrd1	4.574	3.40E-307	3.914	5.61E-130
Icos	4.328	1.21E-237	4.087	8.76E-214
Rgs1	3.999	2.93E-178	4.234	3.72E-160
Vim	4.250	0	3.972	2.11E-116
Tpi1	3.714	0	4.414	4.92E-250
S100a11	4.094	0	3.787	2.85E-122
Gapdh	3.647	0	4.208	2.01E-130
Litaf	4.097	0	3.709	0
Bcl2a1b	4.151	0	3.517	3.37E-297
Ptms	4.012	0	3.556	1.98E-304
Aldoa	3.501	1.34E-242	3.934	5.61E-117
Srgn	3.630	0	3.702	9.78E-109
Ctla4	3.626	0	3.671	5.01E-165
Hif1a	3.565	2.47E-241	3.572	3.55E-128
Sdf4	3.321	6.12E-201	3.755	2.01E-81
Havcr2	3.660	0	3.350	0
Mt1	3.472	0	3.535	0
Fcer1g	3.074	1.05E-258	3.845	1.23E-290
Crabp1	4.385	0	2.489	0
Ndfip1	3.598	2.74E-232	3.271	6.93E-57
Ahnak	3.694	0	3.130	3.41E-110
Ctsb	3.011	1.78E-269	3.737	1.43E-149
Pglyrp1	3.540	6.08E-238	3.174	6.01E-149
Irf8	3.546	0	3.094	2.43E-210
Ifitm3	3.506	0	3.130	2.16E-243

Supplemental Table 5: Differential gene expression between responder TILs and naïve T cells or between non-responder TILs and naïve T cells. Cells in activated clusters of the UMAP (A1-A7) from either responder TIL samples (n = 11615 cells) or non-responder TIL samples (n = 5322 cells) were compared to naïve T cell clusters (N1-N5) from all samples (n = 31215 cells) using Seurat's FindConservedMarkers function to identify differentially expressed genes, controlled for cohort. Compared to naïve T cells, genes most upregulated in responder TILs and in non-responder TILs are shown.

Supplemental Table 6: Differential gene expression between either cells with Responder top clonotypes or cells with Non-responder top clonotypes, compared to Other clonotypes in spleens

Gene	Responder fold change	Responder adjusted p-value	Non-responder fold change	Non-responder adjusted p-value
Gzmf	6.339	6.07E-220	1.000	NA
Ifitm1	10.449	0	5.298	2.01E-142
Ccl4	19.107	0	15.642	1.14E-190
S100a6	11.389	0	8.062	3.22E-125
Tsc22d3	-1.953	1.14E-10	1.000	NA
Ly6c2	1.000	NA	-1.901	6.99E-17
Gm42418	1.000	NA	-1.759	2.45E-37
Ltb	1.000	NA	-1.533	7.83E-26
Jun	-1.469	0.117547678	1.000	NA
Lyz2	3.670	0	6.136	0
Impdh2	-1.436	7.60E-06	1.000	NA
Gzmb	13.959	0	11.783	0
Apoe	3.605	0	5.250	0
Crabp1	4.505	0	2.916	0
Pf4	2.010	1.84E-244	3.321	0
AW112010	5.281	1.37E-256	4.114	6.74E-60
Timp1	2.046	1.01E-242	1.000	NA
C1qb	2.526	9.69E-288	3.550	0
C1qa	2.328	2.56E-257	3.338	0
Rgs16	7.554	0	6.557	0
Camk2n1	1.969	1.27E-155	1.000	NA
Klrk1	3.904	0	2.962	2.48E-137
Gzma	1.000	NA	1.937	1.12E-20
Hmox1	1.389	2.40E-50	2.325	6.08E-79
Cish	1.935	1.01E-35	1.000	NA
Ifng	6.220	8.57E-286	5.291	1.21E-211
Klrc1	6.376	0	5.465	0
Cd68	1.000	NA	1.903	6.00E-40
Ctsb	2.965	6.84E-209	3.825	1.08E-120
Atf3	1.000	NA	1.852	2.67E-160
Cebpb	1.000	NA	1.838	5.01E-11
S100a4	6.493	0	5.672	7.68E-174
Serpinb6b	2.605	5.09E-146	3.397	1.04E-111
Tnfaip3	1.793	0.00462977	1.000	NA
Ctss	1.000	NA	1.784	0.133613862
Ccl6	1.000	NA	1.760	2.71E-155
Bhlhe40	4.696	0	3.940	2.86E-147
Ftl1	1.000	NA	1.755	1.08E-33
C1qc	2.124	4.11E-214	2.877	1.30E-277
Fos	1.000	NA	1.709	0.991727545
Capg	4.448	8.87E-226	3.742	1.13E-137
Ifitm2	4.592	0	3.887	0
Tyrobp	1.814	5.32E-64	2.505	6.55E-71
Nr4a3	1.685	4.01E-57	1.000	NA
Lag3	8.627	0	7.948	0
Klrd1	4.388	1.15E-297	3.732	1.61E-88
Csf1	2.659	2.55E-259	2.002	3.56E-160
Ube2c	1.651	1.52E-42	1.000	NA
Tpi1	4.010	1.87E-287	4.656	6.69E-175
Lgals1	6.012	2.13E-299	5.374	1.27E-103
Cnih2	1.627	9.32E-127	1.000	NA
Irf4	1.619	3.18E-74	1.000	NA

Supplemental Table 6: Differential gene expression between either cells with Responder top clonotypes or cells with Non-responder top clonotypes, compared to Other clonotypes in spleens. Cells were grouped into clonotypes based on the paired amino acid sequences of their CDR3α and CDR3β regions. Clonotypes that made up >0.65% of a responder sample were classified as "Responder top clonotypes" (6858 cells from 58 clonotypes), clonotypes that made up >1% of a non-responder sample were classified as "Non-responder top clonotypes" (3475 cells from 58 clonotypes), and cells that made up <1% of a spleen sample were classified as "Other clonotypes" (33035 cells from 31691 clonotypes). Responder top clonotypes (left) were compared to Other Clonotypes, or Non-responder top clonotypes were compared to Other Clonotypes (right) using Seurat's FindConservedMarkers function (controlling for cohort) to identify differentially expressed genes; the most differentially expressed genes by fold change are shown. R fold change was calculated as the difference in gene expression between cells from R top clonotypes and cells from "Other" clonotypes in spleen and NR fold change was calculated as the difference in gene expression between cells from NR top clonotypes and cells from "Other" clonotypes in spleen.

Supplemental Table 7: Differential gene expression between cells with Responder top TCR clonotypes and cells with Non-responder top TCR clonotypes

Upregulated genes in Responder top clonotypes				
Gene	Adjusted p-value	Fold change	%Expressing in Responders	%Expressing in Non-responders
Ly6c2	5.53E-23	2.356	0.662	0.35225
Ifitm1	0.000427648	1.972	0.5135	0.347
Gm42418	1.39E-05	1.700	1	1
S100a6	1.76E-14	1.413	0.99125	0.96725
Csf1	0.972002535	1.328	0.3645	0.268
Dcn	0.833968973	1.296	0.28925	0.187
Slamf7	0.000244956	1.264	0.36475	0.2805
Ltb	0.237454795	1.263	0.7755	0.72625
Ms4a4b	4.54E-13	1.255	0.977	0.974
Nr4a1	0.001387054	1.247	0.31475	0.26875
Klre1	0.061691422	1.215	0.4015	0.3165
Shisa5	0.000223646	1.215	0.96375	0.94725
Sub1	0.005304161	1.157	0.8775	0.87075
Gadd45b	0.395281353	1.153	0.3135	0.29975
Bcl2a1d	0.076385932	1.149	0.52075	0.49625
Tbx21	0.014314245	1.140	0.41725	0.37725
Gng2	0.87360663	1.137	0.61625	0.5855
Rbpj	0.288648999	1.134	0.51225	0.46425
Btg1	7.36E-07	1.119	0.88225	0.8725
Ccl3	0.486029384	1.068	0.48425	0.503
Havcr2	0.989699097	1.066	0.6095	0.614
Fasl	0.238953281	1.055	0.41975	0.40925
Crip1	0.029915484	1.055	0.92425	0.90325
Gramd3	0.001142449	1.046	0.3455	0.32175
Vgll4	0.623393807	1.040	0.42975	0.41975
Serpine2	0.385665921	1.038	0.28975	0.277
Malat1	0.006159741	1.028	0.98675	0.987
Atf3	0.117730738	1.022	0.217	0.21
Hmga1	0.434162667	1.013	0.23775	0.2665
Ly6e	0.030687987	1.006	0.87975	0.85875

Upregulated genes in Non-responder top clonotypes				
Gene	Adjusted p-value	Fold change	%Expressing in Responders	%Expressing in Non-responders
Hmox1	2.19E-05	1.674	0.1595	0.36025
Lyz2	0.129590777	1.672	0.5585	0.668
Pf4	0.009781024	1.652	0.27275	0.43075
Jun	0.402250018	1.451	0.4915	0.611
Ccl6	0.309115251	1.361	0.1005	0.2295
Ftl1	1.82E-05	1.336	0.929	0.963
Arg1	0.052385233	1.322	0.04775	0.14075
Gzmk	0.076952706	1.317	0.2455	0.3515
Ctss	0.685899371	1.307	0.47575	0.5155
Serpinb6b	4.90E-06	1.304	0.4775	0.6195
Lilrb4a	0.688498733	1.250	0.2185	0.3545
Sat1	0.366084198	1.222	0.5705	0.64825
Lilr4b	0.601308096	1.208	0.15675	0.278
Fth1	0.288307915	1.206	0.99875	0.99975
Ecm1	0.705004124	1.193	0.1825	0.244
Aprt	0.359043766	1.132	0.557	0.61775
Ctsl	0.016529634	1.127	0.46675	0.4545
Gapdh	4.25E-05	1.124	0.96475	0.978
Dap	0.042118789	1.119	0.5245	0.60575
Serpinb9	0.049775932	1.112	0.505	0.588
Mif	0.002212208	1.100	0.85825	0.85175
Nme2	9.71E-05	1.034	0.809	0.80725
Cd226	0.205534248	1.021	0.26425	0.29
Fosb	0.486104836	1.010	0.178	0.1845
Hif1a	0.000994637	1.004	0.76525	0.7805
Dgat1	0.082300673	1.001	0.3065	0.332

Supplemental Table 7: Differential gene expression between cells with Responder top TCR clonotypes and cells with Non-responder top TCR clonotypes. Cells were grouped into clonotypes based on the paired amino acid sequences of their CDR3α and CDR3β regions. Clonotypes that made up >0.65% of a responder sample were classified as “Responder top clonotypes” (6858 cells from 58 clonotypes), clonotypes that made up >1% of a non-responder sample were classified as “Non-responder top clonotypes” (3475 cells from 58 clonotypes), and cells that made up <1% of a spleen sample were classified as “Other clonotypes” (33035 cells from 31691 clonotypes). Responder top clonotypes were compared to Non-responder top clonotypes (left) or the reverse (right) using Seurat’s FindConservedMarkers function (controlling for cohort) to identify differentially expressed genes; the most differentially expressed genes by fold change are shown.

Supplemental Table 8: Flow Cytometry Antibodies used in the study

Antigen	Fluorophore	Company	Product #	Clone	Dilution
Mouse CD3	PE	Biolegend	100307	145-2C11	1:200
Mouse CD4	BV421	BioLegend	100563	RM4-5	1:200
Mouse CD4	BV650	Biolegend	100555	RM4-5	1:200
Mouse CD8a	BV605	BioLegend	100743	53-6.7	1:200
Mouse CD8a	BV711	Biolegend	100747	53-6.7	1:200
Mouse CD8a	BV785	BioLegend	100749	53-6.7	1:200
Mouse CD279 (PD-1)	BV711	BioLegend	135231	29F.1A12	1:200
Mouse CD223 (LAG3)	PE	Biolegend	125207	C9B7W	1:200
Mouse CD366 (Tim-3)	APC	BioLegend	134007	B8.2C12	1:200
Mouse CD44	PerCP-Cy5.5	BioLegend	103032	IM7	1:200
Mouse CD62L	APC/Cy7	BioLegend	104427	MEL-14	1:200
Mouse Granzyme B	FITC	Invitrogen	11-8898-82	NGZB	1:100
Mouse TNFalpha	APC	Biolegend	506308	MP6-XT2L	1:250
Mouse IFNγ	PE	eBioscience	12-7311-41	XMG1.2	1:250
Mouse TCR beta	BV605	BioLegend	109241	H57-597	1:200
Mouse Ly6C	BV421	BioLegend	128031	HK1.4	1:200
Mouse CD278 (ICOS)	FITC	BioLegend	313506	C398.4A	1:200
Mouse Ly6A/E (Sca-1)	PE/Cy7	Biolegend	108114	D7	1:200
Mouse Nur77 (Nr4a1)	PerCP-eFluor710	eBioscience	46-5965-82	12.14	1:100
Mouse EOMES	Alexa 488	eBioscience	53-4875-80	Dan11 MAG	1:100
Mouse Tbet	PE/Cy7	Biolegend	644823	4B10	1:100
Mouse Ki-67	PE/Dazzle 594	Biolegend	652428	16A8	1:100
Mouse NKG2D	PE	Biolegend	115705	C7	1:200
Mouse CD122	PerCP-Cy5.5	Biolegend	123211	TM-β1	1:200
Mouse CD127	FITC	ebioscience	11-1271-81	A7R34	1:100
Mouse CD244	BV605	BD Bioscience	740345	2B4	1:200
Mouse CD25	APC/Cy7	Biolegend	102025	PC61	1:200
Mouse CD49d	PE/Cy7	Biolegend	103618	R1-2	1:200
Mouse CD69	PE/Cy5	BioLegend	104509	H1.2F3	1:200
Mouse KLRG1	PE	Biolegend	138408	2F1/KLRG1	1:200
Mouse CD45	BUV395	BD Bioscience	564279	30-F11	1:200
Mouse CD45	APC/Cy7	Biolegend	103115	30-F11	1:200
Mouse TCRβ	APC	Biolegend	109211	H57-597	1:200
Live/Dead	Green (488)	Invitrogen	L23101	--	1:1000
Live/Dead	Violet (405)	Invitrogen	L34966	--	1:1000
